# Key Factors in Epidemiological Exposure and Insights for Environmental Management: Evidence from Meta-analysis

**DOI:** 10.1016/j.envpol.2024.124991

**Published:** 2024-12

**Authors:** Yongyue Wang, Jie Chang, Piaopiao Hu, Chun Deng, Zhenyu Luo, Junchao Zhao, Zhining Zhang, Wen Yi, Guanlin Zhu, Guangjie Zheng, Shuxiao Wang, Kebin He, Jing Liu, Huan Liu

**Affiliations:** 1 State Environmental Protection Key Laboratory of Sources and Control of Air Pollution Complex, State Key Joint Laboratory of Environmental Simulation and Pollution Control, School of Environment, Tsinghua University, Beijing 100084, China; 2 National Center for Neurological Disorders, Xuanwu Hospital, Capital Medical University, Beijing 100084, China; 3 Centre for Clinical and Epidemiologic Research, Beijing An Zhen Hospital, Capital Medical University, Beijing Institute of Heart, Lung and Blood Vessel Diseases, Beijing 100029, China

**Keywords:** air pollution, cause-specific mortality, Chinese population, long- and short-term epidemiological studies, exposure assessment, exposure level

## Abstract

In recent years, the precision of exposure assessment methods has been rapidly improved and more widely adopted in epidemiological studies. However, such methodological advancement has introduced additional heterogeneity among studies. The precision of exposure assessment has become a potential confounding factors in meta-analyses, whose impacts on effect calculation remain unclear. To explore, we conducted a meta-analysis to integrate the long- and short-term exposure effects of PM_2.5_, NO_2_, and O_3_ on all-cause, cardiovascular, and respiratory mortality in the Chinese population. Literature was identified through Web of Science, PubMed, Scopus, and China National Knowledge Infrastructure before August 28, 2023. Sub-group analyses were performed to quantify the impact of exposure assessment precisions and pollution levels on the estimated risk. Studies achieving merely city-level resolution and population exposure are classified as using traditional assessment methods, while those achieving sub-kilometer simulations and individual exposure are considered finer assessment methods. Using finer assessment methods, the RR (under 10 μg/m^3^ increment, with 95% confidence intervals) for long-term NO_2_ exposure to all-cause mortality was 1.13 (1.05-1.23), significantly higher (p-value=0.01) than the traditional assessment result of 1.02 (1.00-1.03). Similar trends were observed for long-term PM_2.5_ and short-term NO_2_ exposure. A decrease in short-term PM_2.5_ levels led to an increase in the RR for all-cause and cardiovascular mortality, from 1.0035 (1.0016-1.0053) and 1.0051 (1.0021-1.0081) to 1.0055 (1.0035-1.0075) and 1.0086 (1.0061-1.0111), with weak between-group significance (p-value=0.13 and 0.09), respectively. Based on the quantitative analysis and literature information, we summarized four key factors influencing exposure assessment precision under a conceptualized framework: pollution simulation resolution, subject granularity, micro-environment classification, and pollution levels. Our meta-analysis highlighted the urgency to improve pollution simulation resolution, and we provide insights for researchers, policy-makers and the public. By integrating the most up-to-date epidemiological research, our study has the potential to provide systematic evidence and motivation for environmental management.

## 1 Introduction

Air pollution has become the fourth leading cause of premature death worldwide (WHO, 2022). In 2019, the total air pollution resulted in approximately 6.67 million premature mortality globally, with over 1.85 million in China (Stapleton et al., 2008). The six principal air pollutants have received widespread attention in China, among which fine particulate matter (PM_2.5_), ozone (O_3_) and nitrogen dioxide (NO_2_) have been identified as the most critical air pollutants. PM_2.5_ has significant and severe health impacts (Chen and Hoek, 2020; Li et al., 2019b; Orellano et al., 2020). O_3_ emerging as the primary pollutant in summer (Xiao et al., 2021; Yang et al., 2018b). The NO_2_ pollution is not as severe as PM_2.5_ and O_3_, but was identified as the core air pollution precursor of O_3_ and nitrate component of PM_2.5_ (He et al., 2020). In comparison, the health effects of the other three air pollutants, coarse particulate matter (PM_10_), sulfur dioxide (SO_2_) and carbon monoxide (CO), are less important. Exposure effects of CO and SO_2_ remained unclear (WHO, 2021a), and the most considerable effect particle size of PM_10_ is PM_2.5_ (Lin et al., 2016b), so we do not prioritize their attention. Over the past decade, China implemented robust policies to reduce air pollution and significantly improved air quality (Luo et al., 2024; Luo et al., 2022; Shi et al., 2022). The impact of drastic changes in air pollution levels on exposure effects have seen evidence in other world-wide nations (Burnett et al., 2014), however, have not yet undergone systematic analysis on the Chinese population.

Recently, WHO updated its AQG in 2021 based on up-to-date health evidence(WHO, 2021b)(WHO, 2021b). In 2022, key cities in China achieved a 62.8% compliance rate for environmental air quality compared to the Chinese air quality standards. The annual average concentrations of PM_2.5_ and O_3_ at 29 μg/m^3^ and 145 μg/m^3^, respectively. Approximately 1/4 of the cities exceeded the standard level throughout the year (Yang et al., 2018b). Compared with WHO AQG 2021, China has not yet sufficiently mitigated the health risks posed by air pollutants exposure, necessitating further tightening of air quality standards. The United States Environmental Protection Agency (U.S. EPA) has established a comprehensive revision process for air quality standards, requiring consideration of updates based on air quality criteria (Wang et al., 2022). However, the air quality criteria in China are still in their early stages. WHO AQG 2021 may be a potential reference, but its representativeness is questionable due to the inclusion of limited evidence from the Chinese population (6 from mainland China, 3 from Taiwan, 1 from Hong Kong) (WHO, 2021b)(WHO, 2021b). Other developed integrated models may face similar challenges (Zou et al., 2019). Most existing meta-analyses focus on one pollutant, health outcomes, or exposure period. Few studies covered major pollutants and health outcomes; however, they often aggregated worldwide population effect relationships (Orellano et al., 2020). There has yet to be a comprehensive integration of the exposure effects of major air pollutants in China to support the formation of health-based air quality criteria. Systematic evidence synthesis could lead to new insights into population exposure risks, further motivating the policy revisions and air pollution reduction.

Research on the short-term effects of air pollution exposure in China can be traced back to the beginning of this century (Kan and Chen, 2003). Many solely relied on exposure level assessments derived from monitoring station observations (Dong et al., 2012; Zhang et al., 2011). However, variations in pollution levels exist among city regions, and solely adopting city-level averages would indeed introduce biases for analysis (Sun et al., 2023). Since the Chinese government has taken action to reduce air pollution, background levels have gradually decreased. However, near-source pollution effects (where pollution levels increase near the emission source) have become more prominent at the street scale, typically within or around tens of meters (Lv et al., 2022; Wang et al., 2023b). Such localized differences in pollution levels are challenging for traditional medium-scale resolution models or observational data, which typically cover several to tens of kilometers, to accurately represent and account for. In recent years, various small-scale models have been developed to estimate pollution distribution at the street scale (Wang et al., 2023b), and some epidemiological studies have already introduced these finer models into exposure assessments (Wong et al., 2015). Exposure level precision significantly impacts the resultant effects, highlighting the need for clear articulation of these distinctions, which is a limitation often seen in current reviews and meta-analyses (Luo et al., 2023; Yang et al., 2019). These finer methods can help identify exposure hotspots and focus on high-exposure activities (Al-sareji et al., 2022), such as pinpointing highly polluted sections of street environments (Munir et al., 2022). It would allow policy-makers to concentrate their focus from overall exposure to a certain chain, enabling more targeted regulation (Roy et al., 2024). Furthermore, the government will be able to issue specific travel recommendations to the public, enhancing the temporal and spatial flexibility of activity patterns to reduce individual and population exposure risks (Song et al., 2021).

In this study, we investigated whether variations in exposure assessment precision and pollution levels would lead to differences in the calculation of exposure effects. We conducted a meta-analysis to examine the associations between exposure to PM_2.5_, NO_2_, and O_3_ and all-cause, cardiovascular, and respiratory mortality in the Chinese population. This analysis included sub-group evaluations based on exposure assessment precision and pollution levels. From the quantitative analysis and the included literature, we qualitatively identified key factors in exposure assessment relevant to effect calculation. Ultimately, we highlighted the shortcomings of current epidemiological exposure assessments and provided insights for improving environmental management.

## 2 Methods

### 2.1 Literature identification strategies

This report was conducted using the preferred reporting items for systematic reviews and meta-analyses (PRISMA) guidelines. The databases used for identification encompassed Web of Science, PubMed, Scopus, and China National Knowledge Infrastructure (CNKI). The conclusive date for literature identification was August 28, 2023. Relevant literature published before this date was considered the potential candidates, with no specific starting point set. The targeted primary air pollutants were PM_2.5_, NO_2_, and O_3_, and the health outcomes were all-cause, cardiovascular, and respiratory mortality. The study exclusively focused on health evidence from the Chinese population. All literature included in our analysis was conducted on human subjects. We developed a literature identification strategy for each database, combining free text and medical subject headings (MeSH) terms, e.g., target pollutants, health outcomes, study types, and study region, referring to the published meta-analyses with similar topics (Orellano et al., 2020; Yang et al., 2019). The identification logic remained consistent across different databases, listed in Appendix 1. A secondary refinement was conducted for the cited literature in the identification results, serving as an additional relevant source.

Our study was not registered on Cochrane nor PROSPERO. To ensure novelty, we also an identification strategy for existing systematic reviews and meta-analyses, primarily focusing on literature conducted in China. A summary of our findings can be found in Appendix 1. The results have demonstrated that the motivation behind this study is appropriate and necessary. Compared to previous studies, we have made further progress in assessing the impact of 1) employing finer pollution simulation methods and 2) utilizing individual exposure levels instead of group levels on the calculation of the exposure effects.

### 2.2 Eligibility criteria

Our study focuses on both long- and short-term exposure effects of PM_2.5_, NO_2_, and O_3_. Only cohort studies were included for long-term effects, while time-series and case-crossover studies were included for short-term effects. Literature involving the general public (ordinary people) was included to ensure the targeted population’s representativeness. In contrast, literature on occupational air pollution exposure (e.g. firefighters), patients, or populations with particular sensitivity (e.g. children and pregnant women) or high-risk conditions (e.g. smokers) was excluded, because these populations have unique exposure characteristics, including high-intensity and long-duration exposure or sensitive physiological conditions, which fail to represent the general population and thus do not align with the scopes of this study (Chen and Hoek, 2020; Luo et al., 2023; Orellano et al., 2020). Literature that passed the title/abstract screening and met our target were all included in the systematic review, irrespective of whether they were assessed to have a high risk of bias (Lu et al., 2015a). The three major categories of health outcomes of interest were classified using the 10^th^ edition of the International Classification of Diseases (ICD-10), including all-cause non-accidental mortality (ICD-10: A00-R99), cardiovascular disease mortality (ICD-10: I01-I99), and respiratory mortality (ICD-10: J00-J99). Literature that did not provide numeric exposure effects or 95% confidence intervals was excluded. The overall eligibility criteria were summarized in terms of population, intervention/exposure, comparison, outcomes, and type of study (PICOT), shown in Table 1.

For long-term effects, if there were multiple literature analyzing the same cohort, the one with a larger sample size was included to avoid duplicative sampling and ensure the analysis was based on a more reliable statistical analyze (Ghosh et al., 2021). For short-term effects, considering representativeness at national, provincial, municipal, and county scales, priority was given to studies with broader spatiotemporal coverage (Luo et al., 2023). In all cases, multi-city studies were always preferred over single-city studies; for two or more literature on the same study period, study region, and population, with the same pollutants, health outcomes, and data sources, the most recently published one was included (Lu et al., 2015b). On this basis, we further focused on the diversity of exposure assessment methods. Even when considering the same spatiotemporal scope and target population, we included literature that employed different exposure assessment methods to evaluate the potential influence on the estimated exposure effects attributed to its precision. After selecting the more informative literature, the rest were all excluded from the meta-analysis. Additionally, the literature eligible for inclusion in the meta-analysis required a systematic assessment of bias risk. Literature with a high risk of bias were excluded, with detailed descriptions provided in the following part of the Methods section. Following title/abstract screening, bias risk assessment, and consideration of spatiotemporal representativeness, and those passed were finally included in the meta-analysis.

### 2.3 Data extraction

The data extraction process for the chosen literature involved independent extraction by two reviewers, then cross-checking and consolidating the results. Microsoft Excel^®^ was utilized for efficient data extraction. Essential details were meticulously recorded, including the geographical scope, time duration, data source, subject population, sampling size, exposure assessment method, exposure level, health outcome, and more. Association measures such as hazard ratio (HR), relative risk (RR), odds ratio (OR), and excess rate (ER) of the mortality were extracted. Any literature that did not provide detailed effects or had missing effect data was excluded.

For long-term effects, there was often the case that several adjusted models with different confounders were provided in one cohort study. To eliminate the differences in confounding factor adjustment and choose the most suitable model for integration, we compiled the maximum set of confounding factors from the literature included in our research. Then, we made a directed acyclic graph for the associations between air pollution exposures and mortality (shown in Appendix 2). A minimal collection of confounders was developed based on this directed acyclic graph. The selected adjustment model must have the maximum intersection with this minimal set and should preferably not include unnecessary adjustment factors to eliminate the interference of these confounding factors on the study results. For short-term effects, if only a single lag effect for a specific time was provided, that lag effect was selected (Atkinson et al., 2012; Orellano et al., 2020). If multiple lag effects were presented, the selection followed the framework by previous studies (Yang et al., 2019): i) priority was given to lag effects of particular concern to the authors or the most statistically significant effects; ii) non-cumulative lag effects were prioritized, with cumulative lag effects chosen in their absence. Such a framework was devised to choose lag estimates for inclusion in the review without introducing bias (Atkinson et al., 2014).

All included literature should provide at least one effect estimate for each exposure-outcome pair. There was literature assessed multiple air pollutants within the scope of our research and provide evidence for two or more exposure-outcome pairs. These effects from different exposure-outcome pairs were included in corresponding meta-analyses separately (Orellano et al., 2020; Yang et al., 2019).

### 2.4 Risk of bias (ROB) assessment

A full-text review was conducted for all literature identified through title and abstract screening, and relevant information was extracted while simultaneously performing a ROB analysis to assess potential biases. The ROBINS-E risk assessment framework is a state-of-the-art tool for evaluating bias risk in non-randomized exposure studies, and the detailed criteria can be found elsewhere (Higgins et al., 2024). It encompasses seven bias domains: bias due to confounding, bias arising from measurement of the exposure, bias in selection of participants into the study, bias due to post-exposure interventions, bias due to missing data, bias in measurement of the outcome, and bias in selection of the reported result. Each domain is addressed through a series of signaling questions to gather crucial information about the study and the analysis under assessment. Ultimately, the ROBINS-E framework yields three judgments: the existence of bias risk, the potential direction of bias, and whether the bias risk is sufficiently high to threaten the accuracy of the results. The ROBINS-E method was applied to all the literature included in the systematic review. All literature with a high risk of bias was excluded from the meta-analysis.

### 2.5 Data analysis

The exposure-effect relationship may be characterized using HR, RR, and OR in the cohorts, time-series, and case-crossover studies included in this research. Although there exist differences in definitions, these three indicators may not differ significantly in numerical value within the scope of this study. The existing meta-analyses did not further distinguish between them (Southerland et al., 2022). Therefore, we uniformly used the ER to represent effect observations. The HR, RR, and OR were generally taken as RR, and the conversion relationship between ER and RR was as follows (Southerland et al., 2022):

(*Eq*. 1) $$\text{ER}=\frac{\text{RR}-1}{\text{RR}}$$

The standardized increment in pollutant levels associated with the effect is commonly set at a 10 μg/m^3^ change in pollutant levels. RR values based on this specific increment were considered standardized. However, some literature utilized alternative increments in pollutant levels, such as the interquartile range (IQR). For them, the original RR reported in the study is converted to a standardized RR using the following formula (Li et al., 2017; Orellano et al., 2020):

(*Eq*. 2) $${\text{RR}}_{\text{std }}=\text{exp}\left(\frac{10\times \ln\left({\text{RR}}_{\text{org }}\right)}{{\text{Inc}}_{\text{org }}}\right)$$

where: RR_std_ refers to the standardized RR (with a pollutant level increment of 10 μg/m^3^); RR_org_ refers to the RR reported in the literature with a pollutant level increment different from 10 μg/m^3^; Inc_org_ refers to the non-standardized pollutant level increment provided in the literature. Specifically, for O_3_, the concentrations and increments provided in non-mass units (e.g., ppb) in some literature will first be converted to μg/m^3^ before proceeding with further analysis. The p-value was checked for the integrated effects to ensure certainty of significance. A p-value smaller than 0.05 means that the adverse impact of exposure on specific outcomes is significant.

Sub-group analyses were conducted based on gender, age (take 65 as the dividing point), exposure assessment method precisions, and pollution levels:

(1) Age and gender: For literature that provides exposure effects for age and gender sub-groups, we extracted the corresponding sub-group effects for further analysis and comparison. Physiological gender sub-groups include male and female. Age sub-groups were divided into young (<65) and elderly (>=65). It should be noted that some literature had different age divisions, such as using 75 as a dividing point or having more sub-group divisions (e.g., <45, 45-64, >65), which will be manually matched accordingly.
(2) Exposure assessment method precisions: Due to the inclusion of information on population exposure assessment methods (pollutant simulation resolution, subject granularity, etc.), we also conducted sub-group analyses based on the precisions of exposure assessment methods. The population exposure assessments were commonly confined to the urban level. Therefore, literature employing monitoring station observations or pollutant datasets at a comparable scale (e.g., 10 km or coarser resolution grid pollution data) were categorized as lower resolution. Meanwhile, literature utilizing more sophisticated pollution datasets, simulation models were classified as higher resolution (such as adopting land use regression, machine learning algorithms, multi-source fusing data, etc.). As for subject granularity, studies that assess individual exposure levels, such as those using residential addresses for exposure allocation, are considered to have a higher subject granularity in their exposure assessment methodology. Conversely, studies that rely on city-wide mortality data and average pollution levels are deemed to have lower subject granularity. It is important to note that due to the limited number of studies available, we were only able to conduct a subgroup analysis based on subject granularity for the long-term effects of PM_2.5_ on all-cause mortality. The number of studies for the long-term effects of NO_2_ and O_3_ was insufficient. Additionally, almost all studies on short-term effects did not reached individual level, which further restricted the possibility of subgroup analysis.
(3) Pollution levels: Implementing the Action Plan on Air Pollution Prevention and Control after 2013 has significantly contributed to improving air pollution levels. Therefore, taking 2013 as the dividing point, a sub-group analysis was conducted on the included short-term effects to explore the differences in the short-term exposure effects under different pollutant levels. Due to a lack of epidemiological studies, it cannot perform the same analyses for long-term effects. This sub-group analysis did not include the literature that spanned pre- and post-2013 periods. The significance of differences between sub-groups was checked using the interaction analysis and given as the p-value.

After this phase, we employed an integrated conceptualization process based on meta-analysis and subgroup analysis, combining qualitative analysis, prior knowledge, and systematic review together, to identify the key factors influencing the accuracy of exposure assessment methods. The specific steps included: i) Extract information closely related to the research objectives, primarily focusing on the indicators associated with the accuracy of exposure assessment methods and pollution levels. ii) Evaluate the stratification capability among studies of certain exposure-outcome pairs, and determine the comprehensiveness of the included studies. iii) Conduct subgroup analyses for factors that could be stratified. For factors with significant variability (e.g., single layer or too many layers), evaluating their potential impact through literature review and expert judgment, instead of using an over-stratified subgroup analysis. iv) Systematically organize and structure the identified factors based on prior knowledge and reference literature, a process somewhat akin to constructing a directed acyclic graph based on prior knowledge (though not entirely the same). It is important to note that relying solely on qualitative methods may not fully address the heterogeneity among studies and the complexity of detail distribution, potentially leading to bias or the omission of critical factors in statistical analysis. Therefore, we adopted a hybrid approach that combines quantitative analysis with expert judgment based on prior knowledge to enhance the accurate understanding and comprehensive grasp of complex research.

We performed tests to validate heterogeneity among literature to select the most appropriate model. The Cochran’s Q test based on I^2^ and Tau^2^ was taken to examine the heterogeneity of the exposure effects in the included literature and between the sub-groups (Yang et al., 2019). All integrations in this study displayed high heterogeneity (I^2^>40% and p<0.05). Thus, the random-effects model was employed for integration instead of the fixed-effect model.

It is vital to assess the publication bias to check for potential influence during the manuscript formation and publication. Funnel plots were used to evaluate the publication bias in the included meta-literature. Additional algorithms, such as Begg’s and Egger’s regression, were applied to examine the asymmetry of funnel plots quantitatively (Begg and Mazumdar, 1994; Egger et al., 1997). Begg’s test statistic is based on rank correlation algorithms to explore the distribution differences among literature, with the test statistic following a standard normal distribution. Egger’s test statistic is based on weighted linear regression of effect sizes against standard errors, with the test statistic data following a t-distribution. A p-value>0.05 in those regression models would indicate that statistically significant publication bias might exist in the literature. Since prior research recommended conducting asymmetry tests only when the number of included literature in the meta-analysis is greater than or equal to 10 (Sterne et al., 2011), bias tests for publication were performed only for the short-term effects with a sufficient number of included literature.

The widely adopted meta-regression analysis was employed to investigate the sources of heterogeneity. In this study, essential sources of heterogeneity included study region (nation-wide, regional, county-or city-level), duration of study period, study population (the whole adult population, middle-aged or elderly population, elderly population), sampling size (mortality or total population size), exposure assessment method, pollution simulation resolution, exposure level, and study design. Each variable was individually subjected to meta-regression. We assessed the degree of influence of various factors on heterogeneity based on core indicators of meta-regression: the R^2^ statistic (representing the proportion of between-study variance explained by covariates) and ΔI^2^ (a measure of the remaining percentage of variance attributable to between-study heterogeneity after adjustment for predictor variables).

All data analysis and graphical visualization were performed using the statistical software R version 4.3.0 (https://www.r-project.org/) with the “meta” and “metafor” packages. The meta-analysis was performed using the ‘metagen’ function. The symmetry assessment of the funnel plot was performed using the ‘metabias’ function with Begg’s and Egger’s options, respectively. The meta-regression analysis was performed using the ‘metareg’ function.

Visualization was made with the Origin lab and Microsoft PowerPoint^®^. The directed acyclic graph for the associations between air pollution exposures and mortality was created with the help of DAGitty.net (www.dagitty.net, accessed on March 27, 2024).

### 2.6 Sensitivity analysis

In terms of sensitivity analysis, this study employed the leave-one-out method to assess the robustness of the meta-integrated effects. The leave-one-out method involves cyclically omitting one study to conduct a new meta-analysis and comparing the results obtained with including all literature. A p-value>0.05 indicates that omitting that study significantly affects the integrated effects.

Given the differences in study details, particularly in the selection and combination of confounding factors within the effect models, it is crucial to assess the impact of these variations on the final results through sensitivity analysis. While some meta-analyses rely on effect estimates that authors of epidemiological studies tend to report preferentially (Lu et al., 2015a), our study used a minimal set of confounding factors to ensure consistency. We performed separate screenings and meta-analyses using both approaches and compared the results.

Due to the existence of the literature with the study period overlapping with the COVID-19 pandemic (from 2020), a sub-group analysis was performed additionally in the sensitivity analysis to see the potential influence of the inclusion of COVID-19 pandemic period on the mortality risk among the Chinese population.

The aforementioned meta-analysis was solely based on single-pollutant models from the literature. However, in some studies, after adjusting for multiple pollutants, there have been instances where the RR values shift or the significance of the associations changes. Therefore, we performed a sensitivity analysis to observe the impact of considering the multi-pollutant interactions on the final results. The principle for including multi-pollutant models is as follows: For studies that provided multi-pollutant models, we replaced the relevant RR values originally included in the meta-analysis. During the selection process, the lag model or the confounder-adjusted model was kept consistent with the primary analysis to ensure methodological comparability within the meta-analysis. In cases where multiple multi-pollutant models were available, we prioritized: i) the model adjusting for the highest number of pollutants (e.g., a four-pollutant model is prioritized over a two-pollutant model) and ii) the most significant pollutant-adjusted model.

### 2.7 Certainty of evidence

To ascertain the certainty of evidence (CoE) for each combination of exposure and health outcomes, we employed the Grading of Recommendations, Assessment, Development, and Evaluation (GRADE) approach (Orellano et al., 2020). The CoE assessment encompasses eight domains: five for downgrading (limitations in studies, indirectness, inconsistency, imprecision, and publication bias) and three for upgrading (large effect size, confounding, and concentration-response gradient). The overall assessment procedure was as follows: 1) for a specific exposure-health outcome pairing, evidence from multiple articles was initially deemed to have a moderate CoE, and subsequent evaluations occur in both 2) downgrading and 3) upgrading domains. Following the initial analysis, assessments were made for the remaining three domains, allowing for the potential upgrading of CoE. The final results were categorized into four potential overall certainties: high (further research was unlikely to alter confidence in the integrated effects), moderate (additional studies might significantly impact confidence in the integrated effects), low (further research was likely to have a significant effect on confidence in the integrated effects), or very low (high level of uncertainty in estimating the integrated effects). Detailed criteria for evidence certainty assessment are listed in Appendix 3.

## 3 Results

### 3.1 Meta-analysis for long- and short-term exposure effects

**Figure 1:**
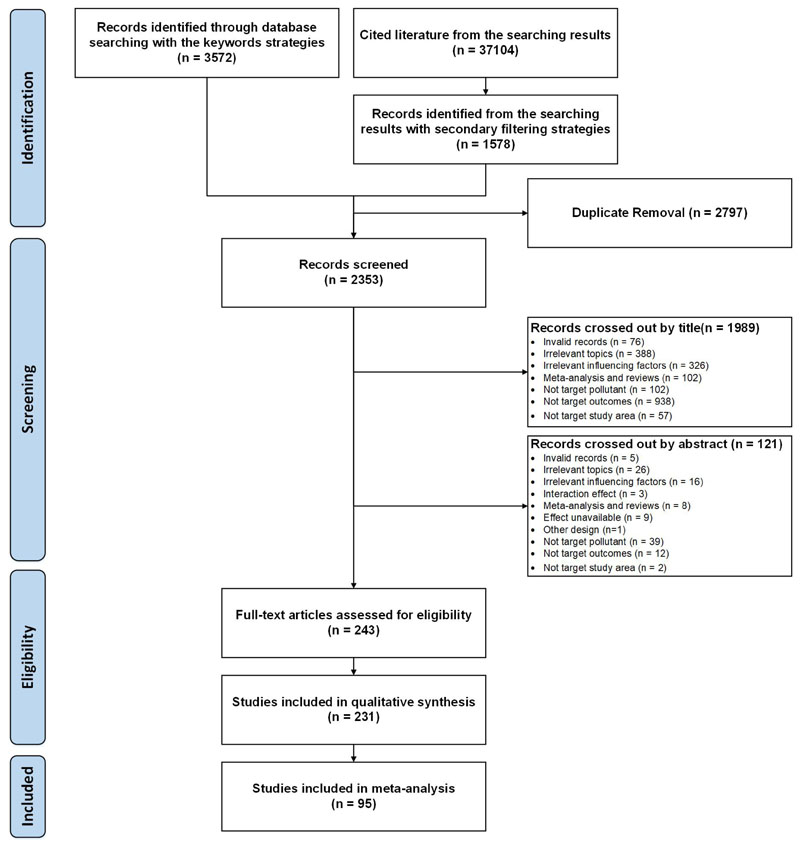
PRISMA flowchart: systematic literature identification and the in-and-exclusion criteria for epidemiological studies in this study.

Among the identified 37104 records from the database, there were 2353 records remained to be screened. Totally 231 records were included in the qualitative analysis, and only 95 studies were included in the final meta-analysis: 21 articles for the long-term effects (Cao et al., 2011; Chen et al., 2021b; Giani et al., 2020; Guo et al., 2022; Huang et al., 2023b; Ji et al., 2022; Liang et al., 2022; Liu et al., 2022a; Liu et al., 2022b; Niu et al., 2022; Wang et al., 2023a; Wang et al., 2023c; Wang et al., 2023d; Xia et al., 2023; Yang et al., 2020; Yang et al., 2018a; Yin et al., 2017a; Yuan et al., 2023; Zhang et al., 2022b; Zhang et al., 2023a; Zhang et al., 2023b) and 74 articles for the short-term effects (Cao et al., 2012; Chen et al., 2023a; Chen et al., 2022a; Chen et al., 2019; Chen et al., 2022b; Chen et al., 2021a; Chen et al., 2022c; Chen et al., 2012; Chen et al., 2017; Chen et al., 2018; Chen et al., 2021c; Chen et al., 2022d; Dai et al., 2015; Deng et al., 2021; Dong et al., 2020; Duan et al., 2019; Gao et al., 2023; Gong et al., 2019; Guan et al., 2022; He, 2022; Hu et al., 2021; Ji et al., 2020; Kan et al., 2010; Kan et al., 2007; Li et al., 2015; Li et al., 2013; Li et al., 2018a; Li et al., 2019a; Lin et al., 2022; Lin et al., 2016a; Liu et al., 2018; Liu et al., 2020b; Liu et al., 2023; Liu et al., 2022c; Liu et al., 2021a; Liu et al., 2021b; Liu et al., 2019b; Lu et al., 2015c; Lu et al., 2023; Ma et al., 2023; Ma et al., 2011; Mo et al., 2023; Peng, 2023; Pu et al., 2023; Qian et al., 2015; Qian et al., 2010; Qiu et al., 2018; Qu et al., 2018; Shao et al., 2021; Song et al., 2022; Sun et al., 2022; Tao et al., 2012; Tian et al., 2020; Wong et al., 2001; Wong et al., 2002; Wu et al., 2022; Wu et al., 2019; Wu et al., 2018; Xu et al., 2020a; Xu et al., 2020b; Xu et al., 2022; Yan et al., 2021; Yang et al., 2012; Yang et al., 2013; Yin et al., 2017b; Yu et al., 2019; Zhai et al., 2023; Zhang et al., 2016; Zhang et al., 2017; Zhang, 2019; Zhang, 2023; Zhang et al., 2020; Zhong et al., 2018; Zhou et al., 2022). Reasons of crossing out identified literature during the whole process were listed in Appendix 4. Lists of the included literature were detailed in Appendix 5 and 6. The study selection process is presented in detail in Figure 1. To ensure the representativeness and coverage of the pollution windows, we summarized the pollution levels among literature for each exposure-outcome pairs in Appendix 7. The substantial variability in pollutant ranges confirms the validity of the integrated effects and underscores the rationale and significance of conducting this work.

For short-term exposure, each 10 μg/m^3^ increase in PM_2.5_ exposure corresponds to RR values (95% CI) of 1.0050 (1.0037-1.0064), 1.0079 (1.0061-1.0097), and 1.0078 (1.0059-1.0097) for all-cause, cardiovascular, and respiratory mortality, respectively. Correspondingly, with the same increment in other pollutants, the RR values of mortality associated with NO_2_ exposure are 1.0149 (1.0108-1.0191), 1.0208 (1.0172-1.0244), and 1.0162 (1.0119-1.0205), and for O_3_, the RR values are 1.0058 (1.0029-1.0087), 1.0064 (1.0036-1.0092), and 1.0066 (1.0038-1.0095). For long-term exposure, each 10 μg/m^3^ increase in PM_2.5_ exposure corresponds to RR values (95% CI) of 1.12 (1.06-1.19), 1.16 (1.09-1.25), and 1.17 (1.01-1.37) for all-cause, cardiovascular, and respiratory mortality, respectively. For the same increment in other pollutants, the increase in NO_2_ exposure is associated with an RR value of 1.11 (1.03-1.20) for all-cause mortality. The increase in O_3_ exposure is associated with an RR value of 1.10 (1.01-1.19) and 1.15 (1.03-1.28) for all-cause and cardiovascular mortality, respectively. The forest maps of the meta-analysis result are shown in Appendix 8.

In our results, we found there was no evidence of long-term O_3_ exposure effects on respiratory mortality. We attempt to explain it from the following three aspects. First, the impact of O_3_ on respiratory diseases remains uncertain. Even at the international level, as reflected in the WHO AQG 2021 (WHO, 2021a), which compiles the most advanced global health evidence, the association between O_3_ and respiratory disease mortality is still classified as “low”. The lack of related evidence in Chinese studies may be possibly because no statistically meaningful results have been obtained. Second, the availability of cohort databases suitable for O_3_ analysis in China is still limited. According to our research findings, only the CLHLS and CHARLS databases have been utilized in studies on all-cause mortality; and the CHERRY and CCDRFS databases on cardiovascular disease mortality. The limited availability and richness of information provided by these databases may restrict the scope of related research. Third, in existing studies on the effects of O_3_ exposure, the research periods span from 2005 to 2018. Despite the use of some advanced pollution simulation methods, various factors may have contributed to significant inaccuracies in the simulation results. These factors include lower early pollution concentrations (Zhang et al., 2022a), a lack of observational data (since China did not incorporate O_3_ into ordinary monitoring until 2013), inaccurate characterization of precursors (Chen et al., 2023b), the complexity of formation mechanisms (Xiong et al., 2023), and significant contributions from cross-regional transport (Liu et al., 2020a). These issues may contribute to the lack of significant associations observed in the results.

The meta-analysis differences in exposure effect among age and gender sub-groups are shown in Appendix 9. Briefly, there exist exposure effect differences among age sub-groups between elderly (≥65) and young (<65), but no statistically significant differences were found between males and females. The results of the ROB assessment (including the ROBINS-E and the funnel symmetry test), the meta-regression analysis, the sensitivity analysis (including: i) the leave-one out validation; ii) confounder difference comparison; iii) the impact analysis of COVID-19 pandemic; iv) the impact of taking multi-pollutant models into consideration), and the certainty analysis can be found in Appendix 10-14. All results showed good robustness and reliability to our results.

### 3.2 The impact of exposure assessment precisions and pollution levels

**Figure 3:**
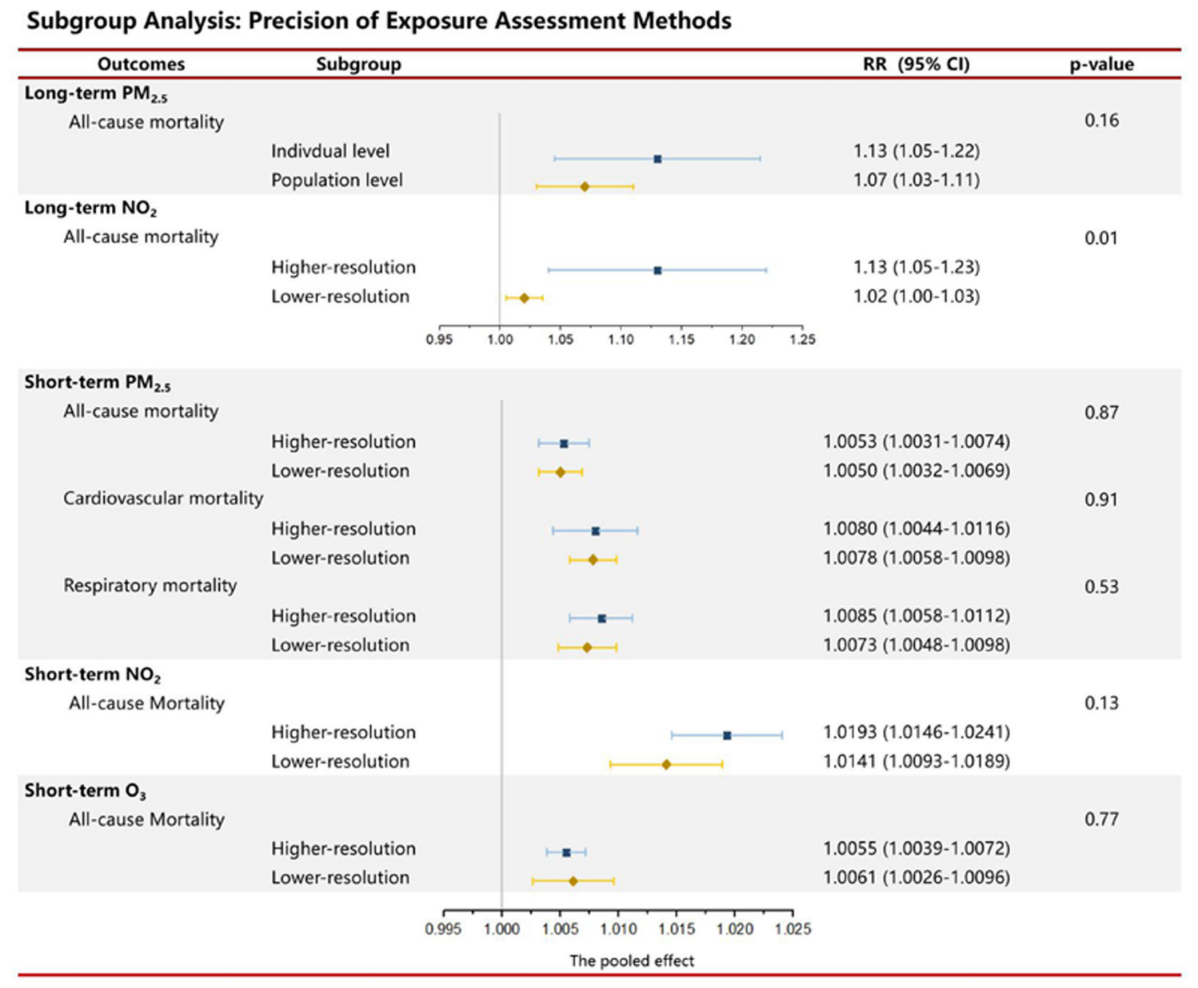
Exposure effects of PM_2.5_, NO_2_, and O_3_ under different method precisions (including subject granularity and pollution simulation resolution) in population exposure assessment on all-cause, cardiovascular, and respiratory mortality

For various exposure assessment methods, sub-group analyses reveal a potential influence of the exposure assessment method precisions on the quantitative estimation of exposure effects, shown in Figure 3. Only the long-term effects of PM_2.5_ on all-cause mortality supported subgroup analysis by subject granularity. Increasing subject granularity to individual exposure level showed a slightly significant impact on effect estimation (with a p-value approaching the 0.1 level). The short-term effects of PM_2.5_, the long- and short-term effects of NO_2_, and the short-term effects of O_3_ supported subgroup analyses based on the resolution of pollution simulations. The refinement of pollution simulation resolution shows a statistically significant difference at the 0.05 level for the long-term effect of NO_2_ on all-cause mortality, and also approaching 0.1 level for short-term NO2 exposure. All these mentioned sub-group analyses for NO_2_ and PM_2.5_ suggest that applying more precise exposure assessment methods tends to yield higher exposure effect values, though with slight significance.

Since the implementation of China’s Action Plan on Air Pollution Prevention and Control in 2013, there have been significant changes in pollution levels. Implementing the Action Plan on Air Pollution Prevention and Control has led to a substantial decrease in PM_2.5_ and NO_2_, while an increase in O_3_. Among the short-term effects of PM_2.5_, there was no significant difference in the effects on respiratory mortality at different pollution levels (p-value=0.63). However, cardiovascular mortality showed a significant difference at the 0.1 level (p-value=0.09) and as similar for all-cause mortality (p-value=0.13). No significant differences were found in short-term exposure effects of NO_2_ and O_3_ at different pollution levels. Despite the insignificance, the short-term exposure effects on all mortality showed a potential tendency to increase as the pollution levels decreased.

### 3.3 Key factors in exposure assessment influencing effect calculation

Sub-group meta-analyses have indicated that the exposure assessment precisions can potentially influence effect calculation. However, the sources of limitations in exposure assessment precisions are multifaceted, necessitating identification of the key factors. Here, we identified four major key factors: pollution simulation resolution, subject granularity, micro-environments classification, and pollution levels.

Pollution simulation resolution is a core factor influencing the exposure assessment precisions, and fundamentally determining the subject granularity, the ability to differentiate micro-environments, and the evaluation of pollution levels. For example, assessments using in-situ monitoring data or pollution datasets with kilometer-scale resolution can only evaluate population exposure at the city or town level, unable to differentiate of micro-environments, and with relatively low accuracy in pollution representativeness. Notably, such limitations depend on the distribution nature of the pollutants. The more a pollutant tends to have the near-source characteristics, the greater the impact of pollution simulation accuracy on exposure assessment precisions (Lal et al., 2020). This is supported by the sub-group analysis. NO_2_, a highly traffic-related pollutant, shows the most significant response to changes in pollution simulation accuracy for both short- and long-term exposures (Xiang et al., 2022). In contrast, PM_2.5_, due to its complex sources and weaker near-source characteristics compared to NO_2_, shows a less significant response to changes in pollution simulation accuracy (Karner et al., 2010). Nevertheless, with the improved capability to identify local hotspots, there is a consistent trend where finer precision leads to higher effect.

Subject granularity (population vs. individual) is another crucial factor. Earlier studies, lacking detailed individual-level data, could only use entire populations as the evaluation target. However, due to the physiological and occupational diversity among individuals, exposure characteristics vary significantly among the population (Gerharz et al., 2009; Shimada and Matsuoka, 2011). It is highlighted by our meta-analyses of long-term PM_2.5_ exposure. Even with limited literature, the use of more precise pollution inputs allowed the sub-group analysis to further quantify the effect differences between individual and population research. The difference in long-term PM_2.5_ exposure effects indicates increasing subject granularity typically enhances the significance of exposure effects (Guo et al., 2020; Shafran-Nathan et al., 2018). Individual studies can more accurately distinguish exposure differences and adjust for potential confounding factors, leading to less biased quantitative results, while the population-level assessment overlooks the exposure differences brought about by the characteristics divergence.

When the subject granularity is refined to the individual level, the importance of differentiating micro-environments (e.g., streets and indoors) becomes apparent. Most of the time, people are exposed in indoor environments, making the adoption of ambient levels as an exposure proxy problematic (Hu et al., 2022; Koistinen et al., 2001; Morawska et al., 2024). Factors like pollutant penetration and filtration (Adams et al., 2001), gaseous processes (Yang et al., 2023), as well as the diversity in source distribution and intensity (Cai et al., 2021; Miao and Ding, 2020; Shimada and Matsuoka, 2011) all contribute to the significant exposure differences among the micro-environments, which is unlikely to be depicted by using ambient levels alone. Among the literature included in the meta-analysis, only one considered the differences in individual NO_2_ exposure across indoor and outdoor micro-environments, deriving a relatively higher quantitative effect (Hu et al., 2021). Such differentiation would be crucial for understanding individual cumulative exposure doses and their health impacts.

The sub-group analyses have shown that lower ambient pollution levels tend to yield higher risk values. However, it is noticeable that pollution simulation precision, subject granularity, and micro-environment differentiation ultimately influence the assessed pollution levels. The close interplay among these influencing factors can result in varying trends in the final results. Further research is needed to disentangle the extent of impact these key factors have on the exposure assessment.

### 3.4 Insights for environmental management

From our study, we identified some limitations in the localization evidence of the main air pollutant exposure to the Chinese population. Firstly, the majority of literature included in the meta-analysis focuses on the long-term effects of PM_2.5_, while NO_2_ and O_3_ remain rare. The cohort studies on the exposure effects of air pollutants in China have only been reported in recent years, and the literature included in this study is still limited, especially for NO_2_ and O_3_. In particular, about half of the cohort studies focus on elderly or middle-aged populations, and there is still a lack of evidence on the long-term effects of air pollution on young people. It could introduce a considerable bias in estimating integrated effect values. Moreover, there is no evidence of long-term O_3_ exposure effects on respiratory mortality in the Chinese population, and the significance of the impact of long-term NO_2_ exposure on cardiovascular and respiratory mortality remained with high divergence. Besides, this study found some publication bias, consistent with the results of other analyses, including meta-analyses conducted within China (Luo et al., 2023) and internationally (Orellano et al., 2020). This bias primarily exists on the right side of the funnel, where some effects are concentrated in the high-value area. Therefore, it cannot be ruled out that RR values may be overestimated in existing studies (Orellano et al., 2020). Also, during the literature identification and exclusion process, we found a lack of research on the air pollution exposure risks of specific occupations in China. Conducting such studies could provide evidence under high-exposure levels and offer potential research opportunities to further explore pollutant exposure mechanisms. Last but not the least, for short-term studies, the exposure is mostly unique for the population, while some has refined to individuals for long-terms studies. Our results have shown the difference and highlight the necessity of the refinement.

To further reduce the risk of population exposure and achieve the grand vision of a “Healthy China” and a “Beautiful China,” based on the limitations of existing studies and key factors affecting the accuracy of exposure assessments, we offer the following insights for environmental researchers, policy-makers and for the public. Researchers form scientific basis for management, policy-makers make direct decisions, and the public serves as the largest audience and practitioners of these efforts.

For researchers: i) continue enriching studies on the exposure effects of air pollutants, especially the long-term exposure effects of NO_2_ and O_3_; 2) identify the independent effect of crucial air pollutant components and explore their interactive effects to lead a deeper understanding of exposure mechanism; iii) further refine the exposure assessment methods, shifting focus from populations to individuals, and distinguishing important exposure micro-environment; iv) make mechanistic improvements to exposure proxies (such as considering activity levels and physiological differences) to reflect exposure differences, e.g., transitioning from concentration assessment to internal exposure assessment. Furthermore, we additionally suggest recommendations for pollutant simulation methods. For NO_2_, due to its regional sources and primary emission from traffic, its temporal and spatial variability is highly significant and pronounced. Therefore, it is recommended to use medium-to small-scale coupled models for detailed simulations at the neighborhood scale (Lv et al., 2022). For O_3_, the choice of simulation model needs to be flexible (Li et al., 2023a), as the formation of O_3_ is closely related to the complex emissions of volatile organic compounds (VOCs) as well as NO_2_. Considered the balance between computational efficiency and representativeness of the reactions (Ren et al., 2022), models that simulate NO_2_ distribution using a basic photochemical reaction mechanism (NO_2_-NO-O_3_) are the most commonly employed. For PM_2.5_ with quite complex sources, detailed modeling of local sources’ impact is needed. A convenient approach is to use the output of medium-scale models, applying machine learning methods for ultra-high-resolution assimilation (Wang et al., 2023b), or to use medium-to small-scale coupled models similar to those used for NO_2_ simulation (Kim et al., 2022).

For policy-makers: i) advocate for the widespread adoption of green technologies and clean energy, particularly in sectors like coal combustion, smelting, and construction, and eventually transit into atmospheric pollution to ultra-low levels to reduce public health risks; ii) imply stricter vehicle emission standards and control non-tailpipe emissions from vehicles, reducing high exposure caused by high emissions in street environments, and similar for other specific micro-environments; iii) enhance atmospheric pollution forecasting and inversion capabilities by integrating multi-source data, machine learning algorithms, and small-scale modeling for the identification of emission and exposure hotspots and ensuring the effective implementation of regulatory measures.

And for the public: i) increase awareness of air pollution and its health impacts, and take appropriate exposure interventions to reduce health risks if necessary; ii) advocate for green lifestyles and reduce individual pollution emissions, especially during periods of heavy pollution; iii) involve in air pollution supervision and made feedback to ensure policy implementation, e.g., report pollution hotspots under no supervision; etc.

## 4 Discussions

Through meta-analysis, we comprehensively synthesized epidemiological evidence regarding the impact of major air pollutants on long- and short-term mortality in the Chinese population. Sufficient evidence from certain exposure-outcome pairs supported sub-group analyses based on exposure assessment precisions and pollution levels. We found that more refined assessment methods and lower pollution levels potentially yield higher exposure effects. This variability drove us to investigate the sources of heterogeneity, ultimately identifying four key factors influencing the impact of exposure assessment precisions on effect calculation, with pollution simulation precisions being the core factor. Based on these analyses, some forward-looking insights were proposed for environmental management in China. We believe our study offers some advantages over single epidemiological studies: i) we integrated a broader range of the most updated evidence, enhancing the accuracy of exposure effects and reducing biases; ii) by reanalyzing a large volume of samples from the literature, we systematically addressed areas that biased studies may not be able to reach; and iii) meta-analysis allows for a systematic evaluation of the complexity and precision of methodologies and their impacts, something that cannot be achieved through individual studies alone.

We exclusively focused on exposure effect evidence within the native Chinese population, revealing potential differences from those in developed countries. There are many debates and discrepancies regarding the effects of exposure in China and globally. The RR (and its 95% CI) for long-term exposure to all-cause mortality in WHO AQG 2021 are reported as 1.08 (1.06-1.09) for PM_2.5_, 1.02 (1.01-1.04) for NO_2_, and 1.01 (1.00-1.02) for O_3_ (WHO, 2021b)(WHO, 2021b). In contrast, the integrated RR in this study is 1.12 (1.06-1.19) for PM_2.5_, 1.11 (1.03-1.20) for NO_2_, and 1.12 (1.01-1.24) for O_3_, significantly higher than the effect from WHO AQG 2021. Similar trends are also reflected in the effects of long-term PM_2.5_ exposure on cardiovascular and respiratory mortality: 1.11 (1.09-1.14) and 1.10 (1.03-1.18) in WHO AQG vs. 1.16 (1.09-1.25) and 1.17 (1.01-1.37) in this study, respectively. The reasons for the higher effects on the Chinese population are complex. Some studies owe it partly to the relatively higher levels of air pollution exposure, the presence of potential differences in sources, and chemical compositions of air pollution in China with unclear interactions among different pollutants and components that may contribute to the differences in exposure effects (Huang et al., 2023a). However, existing explanations are not yet sufficient. Some studies suggest that exposure effects among the Chinese population may be lower than in developed countries, attributing this to potential adaptive responses at higher exposure levels leading to lower impacts (Liu et al., 2019a). The inconsistency in these results requires further in-depth investigation in the future. In comparison, the RR of short-term exposure in this study is comparable to previous research, e.g., 1.0092 (95% CI: 1.0061–1.0123) for cardiovascular mortality, and 1.0073 (95% CI: 1.0029–1.0116) for non-malignant respiratory mortality reported by the WHO AQG 2021 (WHO, 2021b)(WHO, 2021b).

It shows a tendency that the short-term effects of NO_2_ on mortality are greater than those of PM_2.5_ and O_3_, whereas the opposite is observed in the long term. This may reflect potential differences in the exposure mechanisms of different pollutants. NO_2_, as a strong oxidant, may react with water to produce nitrate in the short term, potentially causing more severe oxidative reactions and epithelial cell damage, leading to fatal acute diseases (Huang and Tucker, 2020). Moreover, the long-term effects of NO_2_ exposure on cardiovascular and respiratory mortality in the Chinese population were insignificant. This contrasts with some recent evidence from other regions, e.g., from the ESCAPE project (Beelen et al., 2014; Dimakopoulou et al., 2014). However, in a European cohort study from the ELAPSE project (Brunekreef et al., 2021), long-term NO_2_ exposure showed significant associations with cardiovascular disease (1.089, 1.060-1.120) and respiratory disease (1.101, 1.038-1.168) mortality risks. A study from New Zealand (Hales et al., 2021) presented similar findings. The various scenarios presented in the literature demonstrate inconsistencies in the strength and significance of effects across studies worldwide, not limited to studies conducted in the Chinese region. The literature we included in our study had exposure levels ranging from 40-100 μg/m^3^, while studies from Europe and the United States had lower NO_2_ levels, around 20 μg/m^3^ or even lower. We speculate that one of the reasons for this lack of significance may be the presence of sublinear exposure effects under high pollution levels (Zhang et al., 2021), although this is inconclusive. Additionally, studies have suggested the possibility of exposure misclassification, which may pose a minor threat to the validity of the conclusions (Yorifuji and Kashima, 2020). Moreover, there may be potential uncontrolled confounders that result in substantial changes in correlation between models after a series of adjustments (Yorifuji and Kashima, 2020). Further research is needed to enrich the relevant studies.

The impact of the exposure assessment method precision on calculation of the exposure effect has been proved by the state-of-art epidemiological studies. In a cohort study involving 60 million people in the United States (Di et al., 2017), the exposure effects derived from multisource integrated pollutant data were significantly higher than those from using pollution levels within a 50 km radius area from monitoring stations, regardless of PM_2.5_ or O_3_. This indicates that characterizing exposure at the city level using only the average values from sparse monitoring stations is challenging and may not accurately capture the real differences among populations. Furthermore, sub-group analyses of the long-term exposure effects of PM_2.5_ suggest that refining the assessment from the urban population to individuals can also have an impact. Studies have compared the exposure assessed at the individual level using stochastic exposure simulation methods with those from the traditional average level and found the exposure effect estimated from individual exposure was higher (Chang et al., 2012). This indicates a negative bias in exposure-response response when ambient levels are used as proxies for exposure. Such negative bias may also be introduced by exposure misclassification (Kloog et al., 2013). Most studies allocate exposure levels based on residential addresses, while actual exposure may occur in any corner of the city. Moreover, crude exposure assessment methods may not account for various factors influencing individual exposure and the health effects associated with environmental pollutants, such as higher pollution levels near local emission sources, indoor and outdoor exposure differences, population mobility patterns (Özkaynak et al., 2013), etc. The uncertainty introduced by the methodology may reduce comparability between studies and should not be overlooked (Yang et al., 2019).

The phenomenon that a decrease in PM_2.5_ levels can lead to an increase in RR has been supported by many published studies. As early as the years when the integrated exposure-response curve was developed, it was proposed that the concentration-response function exhibits a sub-linear relationship at higher concentrations (Burnett et al., 2014). Although this argument was revised with the adoption of the global exposure mortality model, it is still evident that at higher concentrations, the slope of the curve shows a slight decline after the cutting point near ~40 μg/m^3^ or even higher (Burnett et al., 2018). Within a wide range of PM_2.5_ levels, a breakpoint has been observed at higher exposure levels. A study involving 1.9 million adults aged 35 to 75 (Li et al., 2023b) found a breakpoint, and other research has identified it near 60.9 μg/m3 in the all-cause mortality curve (Li et al., 2018b), above which the curve became sub-linear and gradually flattens. A few studies have explored the reasons. For example, research published in 2011 attempted to explain it through the adaptability of residents living in areas with severe air pollution (Ma et al., 2011). This argument has been supported by subsequent studies (Cao et al., 2012; Xu et al., 2020b). Additionally, some research suggests that this phenomenon might be due to survivor bias, as susceptible individuals may have passed away before the pollution levels reached such severe levels, leading to a certain degree of bias in the sample (Wong et al., 2008). Finally, the component characteristics of high PM_2.5_ pollution could be significantly different from those of low PM_2.5_ pollution (Zhu et al., 2018), as emission policy scenarios are likely to be totally different, which accounts for the observed differences (Cao et al., 2012). More research is needed to better explain the relationship between pollution levels and RR values and to accumulate additional evidence.

We consider the existing studies included in our research to be the most scientifically rigorous and advanced in terms of methodology, strictly adhering to relevant protocols in the research process. However, regarding the risk of bias, we still observe a high degree of heterogeneity among existing studies, with varying levels of bias or tendency. We raise this for discussion and concern because such biases may not necessarily reflect the actual situation of the research. We observed variability in research details within literature, which may be the potential contributors of the heterogeneity. Factors such as population sampling and structure, as well as inconsistencies in confounding factors, are sometimes limited by the available databases and the specific research regions, leading to systematic biases due to methodological or data structure inconsistencies. However, based on multiple sensitivity analyses, we believe that these differences do not compromise the robustness of our findings. Given the China’s large population and vast geographical coverage, along with the complex array of factors influencing exposure effects, these systemic biases may be objectively present and difficult to avoid. Instead, there might be the possibility that with further research, such biases might potentially serve as representatives of the diversity of exposure effects.

Comparison of pollution control strategies between different countries can highlight the importance of improving the accuracy of monitoring pollution levels. According to the U.S. EPA Air Quality System, approximately 5,000 air quality monitoring sites are operational across the United States. The European Union member states have collectively established around 4,000 air quality monitoring stations. In China, the National Urban Air Quality Monitoring Network comprises nearly 1,800 stations covering 367 key cities. Comparatively, China’s urban air quality monitoring network is on par with those in Western countries, though it is primarily concentrated in central and eastern regions, with relatively lower coverage in suburban areas and cities with lower population density. Many countries have deployed low-cost sensors widely to provide high-resolution real-time data (Feinberg et al., 2019; Jiao et al., 2016), aiding in the identification of pollution sources and hotspot areas. China has also begun deploying low-cost air pollution sensors in major cities and industrial areas (Chao et al., 2021), but there is still room for improvement in the coverage and density of technology and data applications. In addition, some countries have imposed restrictions on population exposure levels. The European Union released the revised Environmental Air Quality Directive in 2022, setting targets to reduce population average exposure levels by 2030. In contrast, China is still focusing on the ambient pollution levels, leaving constraints on population exposure levels in the future plan. The aforementioned differences highlight urgency for more accurate exposure assessment methods and assessment indices in China.

## 5 Limitations

Still, this study has some limitations. The current study was not registered, but we still followed the systematic review procedures rigorously. For long-term effects, due to the limited number of included studies, there may exist estimation deviations to the true effect values, and we could not assess the publication bias of the included studies and exclude the probability of potential publication bias. Although this is acceptable (Yang et al., 2019), further assessment is needed as the evidence evolves. Moreover, some studies have suggested that pollutant exposure may interact with other factors, such as high temperatures, climate, and greenness, and that the effects of various pollutant exposures are not independent. Last but not least, although the independent effects of PM_2.5_ components have received attention, the lack of extensive observational methods and the inherent biases in pollution data introduce considerable uncertainty into the independent effects of various components (Yang et al., 2019). Limited by the existing evidence, this study did not further analyze and discuss these independent effects.

## 6 Conclusions

Our present meta-analysis reveals distinct associations between long- and short-term exposure to three primary air pollutants, PM_2.5_, NO_2_, and O_3_, and an elevated risk of all-cause, cardiovascular, and respiratory mortality in the Chinese population. Finer exposure assessment methods potentially increase the quantified outcomes of exposure effects. There is a tendency for the short-term exposure risk change of pollutants to become larger with the decrease in pollutant levels. Four key factors influencing effect calculation in exposure assessment were identified: pollution simulation resolution, subject granularity, micro-environment classification, and pollution levels. The literature identification and the systematic review highlight the urgent need for more research, improvement in exposure assessment precisions, and pinpoint evaluation for risk management of individual exposure in China. And finally, based on the up-to-date exposure evidence, we provide relevant recommendations for researchers, policy-makers, and the public to further advance exposure assessment methods, promote environmental pollution control, and enhance awareness of environmental health.

## Supplementary Material

Supplementary Material

## Figures and Tables

**Figure 2:**
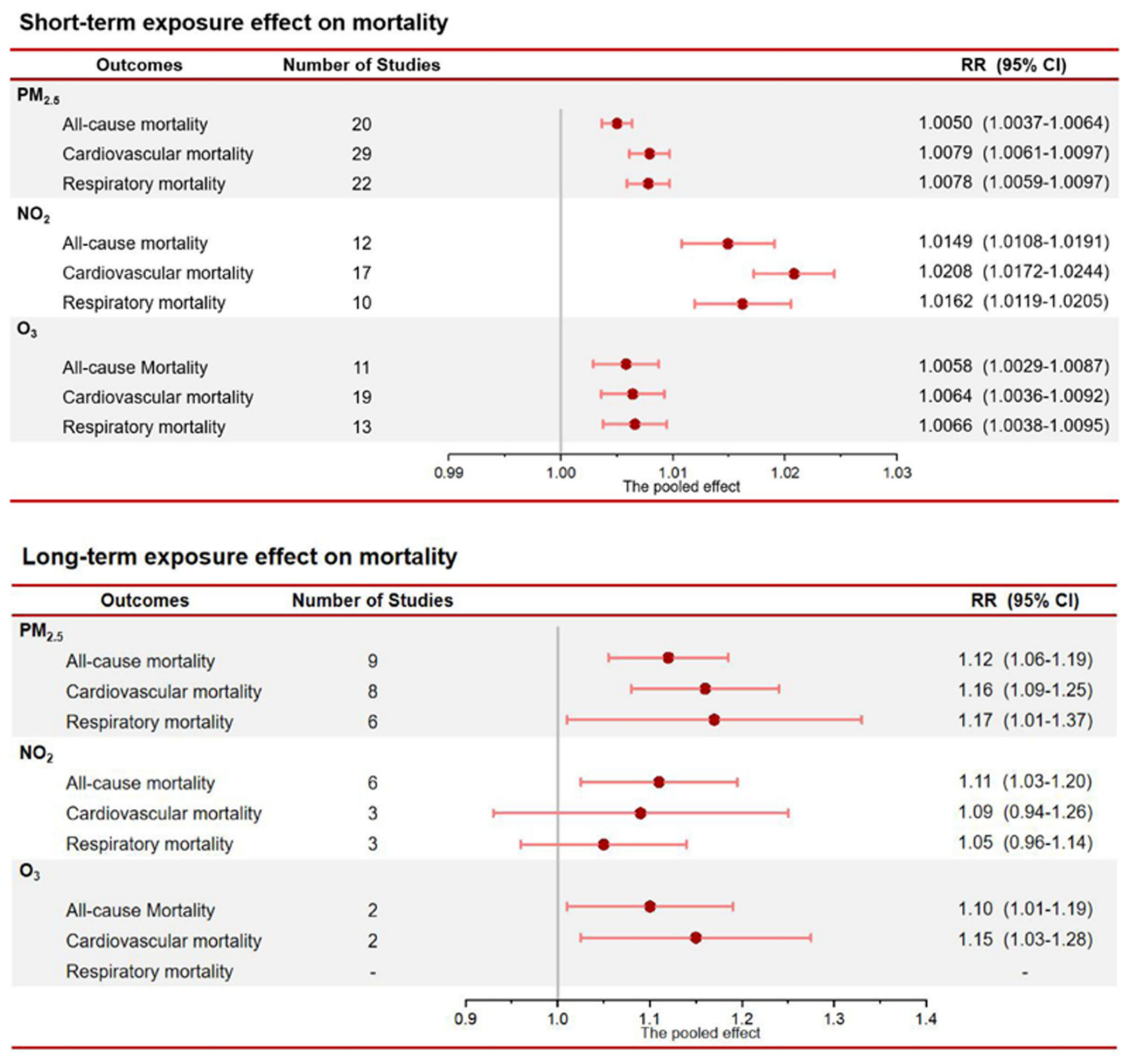
Short- and long-term exposure effects of PM_2.5_, NO_2_, and O_3_ on all-cause, cardiovascular, and respiratory mortality

**Figure 4:**
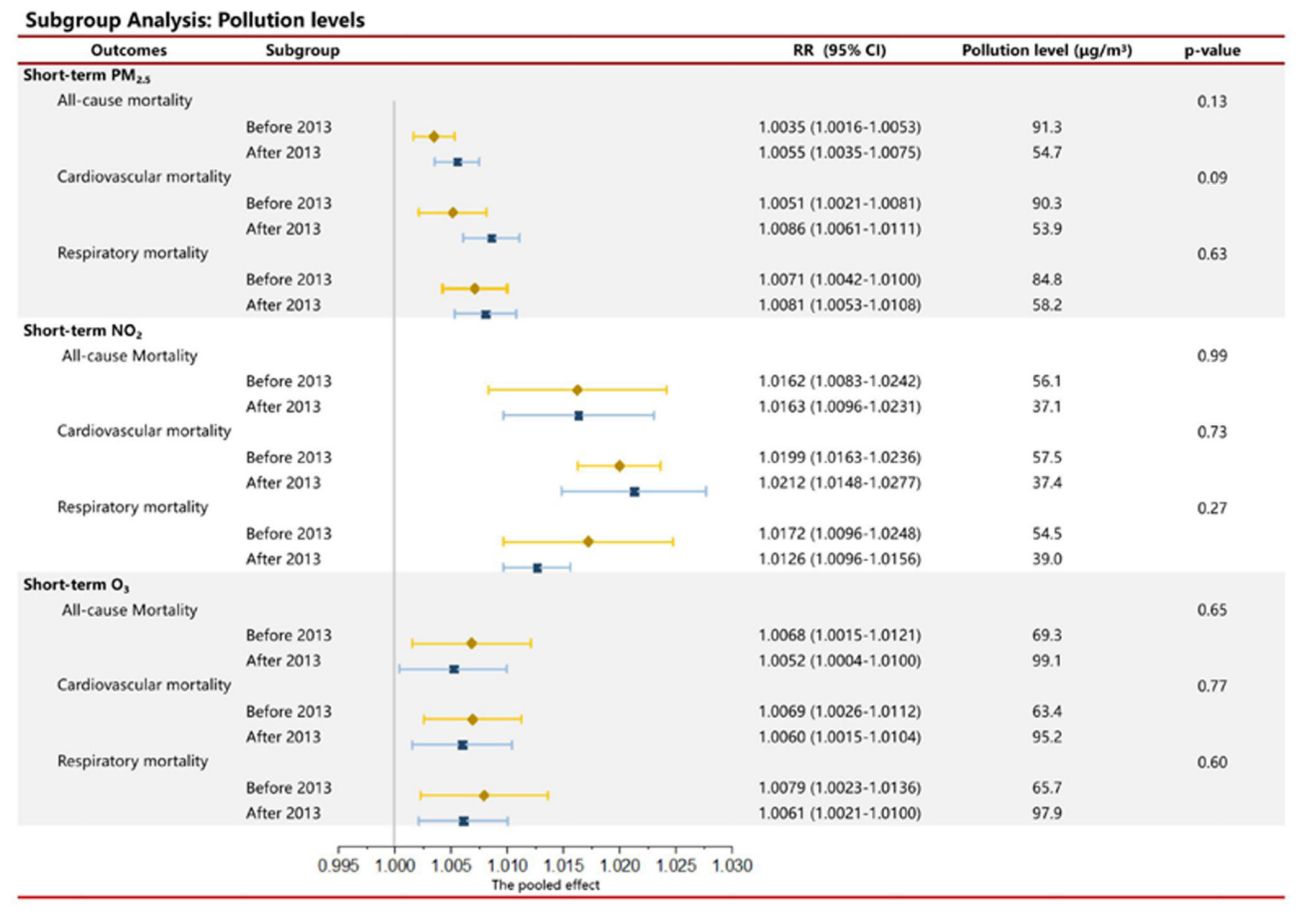
Exposure effects of PM_2.5_, NO_2_, and O_3_ under different pollution levels before and after 2013 on all-cause, cardiovascular, and respiratory mortality

**Table 1:** Table of population, intervention/exposure, comparison, outcomes, and type of study (PICOT)

Terms	Targets
Population	Ordinary Chinese adult population (e.g., not patients, children, or other specific or sensitive populations)
Intervention/Exposure	Ambient (not indoor) PM_2.5_, NO_2_, and O_3_ pollution; exposure derived from all kinds of air pollution observations or simulation models and exposure assessment methods
Comparison	Population exposed under 10.0 μg/m^3^ lower level, or other increments of the mass concentration of air pollutants, or a certain percentage of the data distribution (e.g., IQR)
Outcomes	All-cause, cardiovascular, and respiratory mortality
Type of Study	Cohort studies for long-term exposure; time-series studies and case-crossover studies for short-term exposure

## Highlights

- The meta-analysis integrated local air pollutant exposure evidence for China.
- Finer exposure assessment and lower level tend to derive higher exposure effect.
- Exposure effects of NO_2_ response significant to exposure assessment precisions.
- We identified four key factors in exposure assessment.
- Refined exposure assessment is urgent for precise environmental management.

## Data sharing statement

As this study is a systematic review and meta-analysis of published studies, all data are publicly available in the specified references.

This work is also supported by the Pathways to Equitable Healthy Cities grant from the Wellcome Trust (No. 209376/Z/17/Z). For the purpose of Open Access, the author has applied a CC BY public copyright license to any Author Accepted Manuscript version arising from this submission.

## References

[R1] Adams H, Nieuwenhuijsen M, Colvile R, McMullen M, Khandelwal P (2001). Fine particle (PM_2.5_) personal exposure levels in transport microenvironments, London, UK. Science of The Total Environment.

[R2] Al-sareji OJ, Grmasha RA, Hashim KS, Salman JM, Al-Juboori RA (2022). Personal exposure and inhalation doses to PM_1_ and PM_2.5_ pollution in Iraq: An examination of four transport modes. Building and Environment.

[R3] Atkinson R, Cohen A, Mehta S, Anderson H (2012). Systematic review and meta-analysis of epidemiological time-series studies on outdoor air pollution and health in Asia. Air Quality, Atmosphere & Health.

[R4] Atkinson RW, Kang S, Anderson HR, Mills IC, Walton HA (2014). Epidemiological time series studies of PM_2.5_ and daily mortality and hospital admissions: a systematic review and meta-analysis. Thorax.

[R5] Beelen R, Raaschou-Nielsen O, Stafoggia M, Andersen ZJ, Weinmayr G, Hoffmann B, Wolf K, Samoli E, Fischer P, Nieuwenhuijsen M (2014). Effects of long-term exposure to air pollution on natural-cause mortality: an analysis of 22 European cohorts within the multicentre ESCAPE project. The Lancet.

[R6] Begg CB, Mazumdar M (1994). Operating characteristics of a rank correlation test for publication bias. Biometrics.

[R7] Brunekreef B, Strak M, Chen J, Andersen ZJ, Atkinson R, Bauwelinck M, Bellander T, Boutron M-C, Brandt J, Carey I (2021). Mortality and morbidity effects of long-term exposure to low-level PM_2.5_, bc, NO_2_, and O_3_: an analysis of European cohorts in the ELAPSE project. Research Reports: Health Effects Institute 2021.

[R8] Burnett R, Chen H, Szyszkowicz M, Fann N, Hubbell B, Pope CA, Apte JS, Brauer M, Cohen A, Weichenthal S (2018). Global estimates of mortality associated with long-term exposure to outdoor fine particulate matter. Proceedings of the National Academy of Sciences.

[R9] Burnett RT, Pope CA, Ezzati M, Olives C, Lim SS, Mehta S, Shin HH, Singh G, Hubbell B, Brauer M (2014). An integrated risk function for estimating the global burden of disease attributable to ambient fine particulate matter exposure. Environmental Health Perspectives.

[R10] Cai C, Sun Z, Weschler LB, Li T, Xu W, Zhang Y (2021). Indoor air quality in schools in Beijing: Field tests, problems and recommendations. Building and Environment.

[R11] Cao J, Xu H, Xu Q, Chen B, Kan H (2012). Fine Particulate Matter Constituents and Cardiopulmonary Mortality in a Heavily Polluted Chinese City. Environmental Health Perspectives.

[R12] Cao J, Yang C, Li J, Chen R, Chen B, Gu D, Kan H (2011). Association between long-term exposure to outdoor air pollution and mortality in China: A cohort study. Journal of Hazardous Materials.

[R13] Chang HH, Fuentes M, Frey HC (2012). Time series analysis of personal exposure to ambient air pollution and mortality using an exposure simulator. Journal of Exposure Science & Environmental Epidemiology.

[R14] Chao C-Y, Zhang H, Hammer M, Zhan Y, Kenney D, Martin RV, Biswas P (2021). Integrating fixed monitoring systems with low-cost sensors to create high-resolution air quality maps for the Northern China Plain Region. ACS Earth and Space Chemistry.

[R15] Chen C, Li T, Sun Q, Shi W, He MZ, Wang J, Liu J, Zhang M, Jiang Q, Wang M, Shi X (2023a). Short-term exposure to ozone and cause-specific mortality risks and thresholds in China: Evidence from nationally representative data, 2013-2018. Environment International.

[R16] Chen H, Li X, Su X, Han R, Dong L (2022a). Time-series analysis on health effects of atmospheric ozone exposure on non-accidental deaths in Kunming during 2017-2019. Journal of Environmental and Occupational Medicine.

[R17] Chen J, Hoek G (2020). Long-term exposure to PM and all-cause and cause-specific mortality: A systematic review and meta-analysis. Environment International.

[R18] Chen J, Zeng J, Shi C, Liu R, Lu R, Mao S, Zhang L (2019). Associations between short-term exposure to gaseous pollutants and pulmonary heart disease-related mortality among elderly people in Chengdu, China. ENVIRONMENTAL HEALTH.

[R19] Chen Q, Chen Q, Wang Q, Xu R, Liu T, Liu Y, Ding Z, Sun H (2022b). Particulate matter and ozone might trigger deaths from chronic ischemic heart disease. Ecotoxicology and Environmental Safety.

[R20] Chen Q, Wang Q, Xu B, Xu Y, Ding Z, Sun H (2021a). Air pollution and cardiovascular mortality in Nanjing, China: Evidence highlighting the roles of cumulative exposure and mortality displacement. Chemosphere.

[R21] Chen Q, Wang Q, Xu B, Xu Y, Ding Z, Zhou J, Sun H (2022c). Cumulative effects of ambient particulate matter pollution on deaths: A multicity analysis of mortality displacement. Chemosphere.

[R22] Chen R, Samoli E, Wong CM, Huang W, Wang Z, Chen B, Kan H, Group CC (2012). Associations between short-term exposure to nitrogen dioxide and mortality in 17 Chinese cities: the China Air Pollution and Health Effects Study (CAPES). Environment International.

[R23] Chen R, Yin P, Meng X, Liu C, Wang L, Xu X, Ross JA, Tse LA, Zhao Z, Kan H (2017). Fine particulate air pollution and daily mortality. A nationwide analysis in 272 Chinese cities. American Journal of Respiratory and Critical Care Medicine.

[R24] Chen R, Yin P, Meng X, Wang L, Liu C, Niu Y, Lin Z, Liu Y, Liu J, Qi J (2018). Associations between ambient nitrogen dioxide and daily cause-specific mortality: evidence from 272 Chinese cities. Epidemiology.

[R25] Chen X, Wang M, He TL, Jiang Z, Zhang Y, Zhou L, Liu J, Liao H, Worden H, Jones D (2023b). Data-and Model-Based Urban O_3_ Responses to NO*_x_* Changes in China and the United States. Journal of Geophysical Research: Atmospheres.

[R26] Chen Y, Chen R, Chen Y, Dong X, Zhu J, Liu C, van Donkelaar A, Martin RV, Li H, Kan H, Jiang Q (2021b). The prospective effects of long-term exposure to ambient PM_2.5_ and constituents on mortality in rural East China. Chemosphere.

[R27] Chen Y, Jiao Z, Chen P, Fan L, Zhou X, Pu Y, Du W, Yin L (2021c). Short-term effect of fine particulate matter and ozone on non-accidental mortality and respiratory mortality in Lishui district, China. BMC PUBLIC HEALTH.

[R28] Chen Z, Cui X, Wang B, Hu Y, Dai L, Cao X, Wang C, Shi T (2022d). A time series study on the effect of low air pollution level of NO_2_ on the death of cardiovascular and cerebrovascular diseases in residents. Journal of Public Health and Preventive Medicine.

[R29] Dai J, Chen R, Meng X, Yang C, Zhao Z, Kan H (2015). Ambient air pollution, temperature and out-of-hospital coronary deaths in Shanghai, China. Environmental Pollution.

[R30] Deng J, Hu X, Xiao C, Pan F (2021). The association between gaseous pollutants and non-accidental mortality: a time series study. Environmental Geochemistry and Health.

[R31] Di Q, Wang Y, Zanobetti A, Wang Y, Koutrakis P, Choirat C, Dominici F, Schwartz JD (2017). Air Pollution and Mortality in the Medicare Population. N Engl J Med.

[R32] Dimakopoulou K, Samoli E, Beelen R, Stafoggia M, Andersen ZJ, Hoffmann B, Fischer P, Nieuwenhuijsen M, Vineis P, Xun W (2014). Air pollution and nonmalignant respiratory mortality in 16 cohorts within the ESCAPE project. American Journal of Respiratory and Critical Care Medicine.

[R33] Dong G-H, Zhang P, Sun B, Zhang L, Chen X, Ma N, Yu F, Guo H, Huang H, Lee YL (2012). Long-term exposure to ambient air pollution and respiratory disease mortality in Shenyang, China: a 12-year population-based retrospective cohort study. Respiration.

[R34] Dong Z, Wang H, Yin P, Wang L, Chen R, Fan W, Xu Y, Zhou M (2020). Time-weighted average of fine particulate matter exposure and cause-specific mortality in China: a nationwide analysis. The Lancet Planetary Health.

[R35] Duan Y, Liao Y, Li H, Yan S, Zhao Z, Yu S, Fu Y, Wang Z, Yin P, Cheng J (2019). Effect of changes in season and temperature on cardiovascular mortality associated with nitrogen dioxide air pollution in Shenzhen, China. Science of The Total Environment.

[R36] Egger M, Smith GD, Schneider M, Minder C (1997). Bias in meta-analysis detected by a simple, graphical test. BMJ.

[R37] Feinberg SN, Williams R, Hagler G, Low J, Smith L, Brown R, Garver D, Davis M, Morton M, Schaefer J (2019). Examining spatiotemporal variability of urban particulate matter and application of high-time resolution data from a network of low-cost air pollution sensors. Atmospheric Environment.

[R38] Gao P, Wu Y, He L, Wang L, Fu Y, Chen J, Zhang F, Krafft T, Martens P (2023). Adverse short-term effects of ozone on cardiovascular mortalities modified by season and temperature: a time-series study. FRONTIERS IN PUBLIC HEALTH.

[R39] Gerharz LE, Krüger A, Klemm O (2009). Applying indoor and outdoor modeling techniques to estimate individual exposure to PM_2.5_ from personal GPS profiles and diaries: a pilot study. Science of The Total Environment.

[R40] Ghosh R, Causey K, Burkart K, Wozniak S, Cohen A, Brauer M (2021). Ambient and household PM_2.5_ pollution and adverse perinatal outcomes: A meta-regression and analysis of attributable global burden for 204 countries and territories. PLoS Med.

[R41] Giani P, Castruccio S, Anav A, Howard D, Hu W, Crippa P (2020). Short-term and long-term health impacts of air pollution reductions from COVID-19 lockdowns in China and Europe: a modelling study. LANCET PLANETARY HEALTH.

[R42] Gong T, Sun Z, Zhang X, Wang S (2019). Natural and social factor as modifiers of the effects of PM_2.5_ on daily cardiovascular mortality in Beijing, China. China Environ Sci.

[R43] Guan M, Sun C, Tang D, Kang H, Chen F (2022). A Time-Series Analysis on the Association Between Fine Particulate Matter and Daily Mortality—Shijiazhuang City, Hebei Province, China, 2015–2020. China CDC Weekly.

[R44] Guo C, Yu T, Lin C, Chang L-Y, Bo Y, Wong MC, Tam T, Lau AK, Lao XQ (2022). Habitual exercise, air pollution, and pneumonia mortality: a longitudinal cohort study of approximately 0.4 million adults. American Journal of Epidemiology.

[R45] Guo H, Zhan Q, Ho HC, Yao F, Zhou X, Wu J, Li W (2020). Coupling mobile phone data with machine learning: How misclassification errors in ambient PM_2.5_ exposure estimates are produced?. Science of The Total Environment.

[R46] Hales S, Atkinson J, Metcalfe J, Kuschel G, Woodward A (2021). Long term exposure to air pollution, mortality and morbidity in New Zealand: cohort study. Science of The Total Environment.

[R47] He MZ, Kinney PL, Li T, Chen C, Sun Q, Ban J, Wang J, Liu S, Goldsmith J, Kioumourtzoglou M-A (2020). Short-and intermediate-term exposure to NO_2_ and mortality: a multi-county analysis in China. Environmental Pollution.

[R48] He ZH, Shasha (2022). Time series analysis of effects of ozone exposure on human mortality in Nantong,China,2017 to 2020. Journal of Environmental Hygiene.

[R49] Higgins JPT, Morgan RL, Rooney AA, Taylor KW, Thayer KA, Silva RA, Lemeris C, Akl EA, Bateson TF, Berkman ND, Glenn BS (2024). A tool to assess risk of bias in non-randomized follow-up studies of exposure effects (ROBINS-E). Environment International.

[R50] Hu Y, Ji JS, Zhao B (2022). Deaths attributable to indoor PM_2.5_ in urban China when outdoor air meets 2021 WHO air quality guidelines. Environmental Science & Technology.

[R51] Hu Y, Liu C, Chen R, Kan H, Zhou M, Zhao B (2021). Associations between total mortality and personal exposure to outdoor-originated NO_2_ in 271 Chinese cities. Atmospheric Environment.

[R52] Huang W, Zhou Y, Chen X, Zeng X, Knibbs LD, Zhang Y, Jalaludin B, Dharmage SC, Morawska L, Guo Y (2023a). Individual and joint associations of long-term exposure to air pollutants and cardiopulmonary mortality: a 22-year cohort study in Northern China. The Lancet Regional Health–Western Pacific.

[R53] Huang W, Zhou Y, Chen X, Zeng X, Knibbs LD, Zhang Y, Jalaludin B, Dharmage SC, Morawska L, Guo Y, Yang X (2023b). Individual and joint associations of long-term exposure to air pollutants and cardiopulmonary mortality: a 22-year cohort study in Northern China. The Lancet Regional Health - Western Pacific.

[R54] Huang Y, Tucker J (2020). A handbook of environmental toxicology: human disorders and ecotoxicology.

[R55] Ji JS, Liu L, Zhang J, Kan H, Zhao B, Burkart KG, Zeng Y (2022). NO_2_ and PM_2.5_ air pollution co-exposure and temperature effect modification on pre-mature mortality in advanced age: a longitudinal cohort study in China. ENVIRONMENTAL HEALTH.

[R56] Ji S, Zhou Q, Jiang Y, He C, Chen Y, Wu C, Liu B (2020). The interactive effects between particulate matter and heat waves on circulatory mortality in Fuzhou, China. International Journal of Environmental Research and Public Health.

[R57] Jiao W, Hagler G, Williams R, Sharpe R, Brown R, Garver D, Judge R, Caudill M, Rickard J, Davis M (2016). Community Air Sensor Network (CAIRSENSE) project: evaluation of low-cost sensor performance in a suburban environment in the southeastern United States. Atmospheric measurement techniques.

[R58] Kan H, Chen B (2003). A case-crossover analysis of air pollution and daily mortality in Shanghai. Journal of occupational health.

[R59] Kan H, Chen B, Zhao N, London SJ, Song G, Chen G, Zhang Y, Jiang L (2010). Part 1. A time-series study of ambient air pollution and daily mortality in Shanghai, China. Research report (Health Effects Institute).

[R60] Kan H, London SJ, Chen G, Zhang Y, Song G, Zhao N, Jiang L, Chen B (2007). Differentiating the effects of fine and coarse particles on daily mortality in Shanghai, China. Environment International.

[R61] Karner AA, Eisinger DS, Niemeier DA (2010). Near-roadway air quality: synthesizing the findings from real-world data. Environmental Science & Technology.

[R62] Kim Y, Lugon L, Maison A, Sarica T, Roustan Y, Valari M, Zhang Y, André M, Sartelet K (2022). MUNICH v2.0: a street-network model coupled with SSH-aerosol (v1.2) for multi-pollutant modelling. Geoscientific Model Development Discussions.

[R63] Kloog I, Ridgway B, Koutrakis P, Coull BA, Schwartz JD (2013). Long-and short-term exposure to PM_2.5_ and mortality: using novel exposure models. Epidemiology.

[R64] Koistinen KJ, Hänninen O, Rotko T, Edwards RD, Moschandreas D, Jantunen MJ (2001). Behavioral and environmental determinants of personal exposures to PM_2.5_ in EXPOLIS–Helsinki, Finland. Atmospheric Environment.

[R65] Lal RM, Ramaswami A, Russell AG (2020). Assessment of the Near-Road (monitoring) Network including comparison with nearby monitors within US cities. Environmental Research Letters.

[R66] Li G, Chen Q, Zhu Y, Sun W, Guo W, Zhang R, Zhu Y, She J (2023a). Effects of chemical boundary conditions on simulated O_3_ concentrations in China and their chemical mechanisms. Science of The Total Environment.

[R67] Li J, Woodward A, Hou X-Y, Zhu T, Zhang J, Brown H, Yang J, Qin R, Gao J, Gu S, Li J (2017). Modification of the effects of air pollutants on mortality by temperature: A systematic review and meta-analysis. Science of The Total Environment.

[R68] Li P, Xin J, Wang Y, Li G, Pan X, Wang S, Cheng M, Wen T, Wang G, Liu Z (2015). Association between particulate matter and its chemical constituents of urban air pollution and daily mortality or morbidity in Beijing City. Environmental Science and Pollution Research.

[R69] Li P, Xin J, Wang Y, Wang S, Li G, Pan X, Liu Z, Wang L (2013). The acute effects of fine particles on respiratory mortality and morbidity in Beijing, 2004–2009. Environmental Science and Pollution Research.

[R70] Li T, Yan M, Sun Q, Anderson GB (2018a). Mortality risks from a spectrum of causes associated with wide-ranging exposure to fine particulate matter: A case-crossover study in Beijing, China. Environment International.

[R71] Li T, Zhang Y, Wang J, Xu D, Yin Z, Chen H, Lv Y, Luo J, Zeng Y, Liu Y, Kinney PL (2018b). All-cause mortality risk associated with long-term exposure to ambient PM_2.5_ in China: a cohort study. The Lancet Public Health.

[R72] Li W, Pei L, Li A, Luo K, Cao Y, Li R, Xu Q (2019a). Spatial variation in the effects of air pollution on cardiovascular mortality in Beijing, China. Environmental Science and Pollution Research.

[R73] Li W, Tian A, Shi Y, Chen B, Ji R, Ge J, Su X, Pu B, Lei L, Ma R (2023b). Associations of long-term fine particulate matter exposure with all-cause and cause-specific mortality: results from the ChinaHEART project. The Lancet Regional Health–Western Pacific.

[R74] Li X, Jin L, Kan H (2019b). Air pollution: a global problem needs local fixes. Nature.

[R75] Liang R, Chen R, Yin P, van Donkelaar A, Martin RV, Burnett R, Cohen AJ, Brauer M, Liu C, Wang W, Lei J (2022). Associations of long-term exposure to fine particulate matter and its constituents with cardiovascular mortality: A prospective cohort study in China. Environment International.

[R76] Lin C, Ma Y, Liu R, Shao Y, Ma Z, Zhou L, Jing Y, Bell ML, Chen K (2022). Associations between short-term ambient ozone exposure and cause-specific mortality in rural and urban areas of Jiangsu, China. ENVIRONMENTAL RESEARCH.

[R77] Lin H, Ma W, Qiu H, Vaughn MG, Nelson EJ, Qian Z, Tian L (2016a). Is standard deviation of daily PM_2.5_ concentration associated with respiratory mortality?. Environmental Pollution.

[R78] Lin H, Tao J, Du Y, Liu T, Qian Z, Tian L, Di Q, Rutherford S, Guo L, Zeng W, Xiao J (2016b). Particle size and chemical constituents of ambient particulate pollution associated with cardiovascular mortality in Guangzhou, China. Environmental Pollution.

[R79] Liu C, Chen R, Sera F, Vicedo-Cabrera AM, Guo Y, Tong S, Coelho MS, Saldiva PH, Lavigne E, Matus P (2019a). Ambient particulate air pollution and daily mortality in 652 cities. New England Journal of Medicine.

[R80] Liu H, Meng Z, Shang Y, Lv Z, Jin X, Fu M, He K (2018). Shipping emission forecasts and cost-benefit analysis of China ports and key regions’ control. Environ Pollut.

[R81] Liu H, Zhang M, Han X (2020a). A review of surface ozone source apportionment in China. ATMOSPHERIC AND OCEANIC SCIENCE LETTERS.

[R82] Liu J, Ruan Y, Wu Q, Ma Y, He X, Li L, Li S, Niu J, Luo B (2020b). Has the mortality risk declined after the improvement of air quality in an ex-heavily polluted Chinese city-Lanzhou?. Chemosphere.

[R83] Liu L, Luo S, Zhang Y, Yang Z, Zhou P, Mo S, Zhang Y (2022a). Longitudinal impacts of PM_2.5_ constituents on adult mortality in China. Environmental Science & Technology.

[R84] Liu S, Zhang Y, Ma R, Liu X, Liang J, Lin H, Shen P, Zhang J, Lu P, Tang X, Li T (2022b). Long-term exposure to ozone and cardiovascular mortality in a large Chinese cohort. Environment International.

[R85] Liu T, Gong W, Zhou C, Bai G, Meng R, Huang B, Zhang H, Xu Y, Hu R, Hou Z (2023). Mortality burden based on the associations of ambient PM_2.5_ with cause-specific mortality in China: Evidence from a death-spectrum wide association study (DWAS). Ecotoxicology and Environmental Safety.

[R86] Liu W, Wei J, Cai M, Qian Z, Long Z, Wang L, Vaughn MG, Aaron HE, Tong X, Li Y, Yin P (2022c). Particulate matter pollution and asthma mortality in China: A nationwide time-stratified case-crossover study from 2015 to 2020. Chemosphere.

[R87] Liu X, Wen X, Sun X, Hong Q, Wang Q, Kang Z, Xia S, Yang C, Zhu S (2021a). The short-term effects of ambient air pollutants are associated with daily mortality in northeast China from 2014 to 2018: a time series analysis. Journal of Occupational and Environmental Medicine.

[R88] Liu Y, Pan J, Fan C, Xu R, Wang Y, Xu C, Xie S, Zhang H, Cui X, Peng Z (2021b). Short-term exposure to ambient air pollution and mortality from myocardial infarction. Journal of the American College of Cardiology.

[R89] Liu Y, Pan J, Zhang H, Shi C, Li G, Peng Z, Ma J, Zhou Y, Zhang L (2019b). Short-term exposure to ambient air pollution and asthma mortality. American Journal of Respiratory and Critical Care Medicine.

[R90] Lu F, Xu D, Cheng Y, Dong S, Guo C, Jiang X, Zheng X (2015a). Systematic review and meta-analysis of the adverse health effects of ambient PM_2.5_ and PM_10_ pollution in the Chinese population. ENVIRONMENTAL RESEARCH.

[R91] Lu F, Xu D, Cheng Y, Dong S, Guo C, Jiang X, Zheng X (2015b). Systematic review and meta-analysis of the adverse health effects of ambient PM2.5 and PM10 pollution in the Chinese population. Environ Res.

[R92] Lu F, Zhou L, Xu Y, Zheng T, Guo Y, Wellenius GA, Bassig BA, Chen X, Wang H, Zheng X (2015c). Short-term effects of air pollution on daily mortality and years of life lost in Nanjing, China. Science of The Total Environment.

[R93] Lu Y, Liu X, Liu Y, Chen N, Gao H, Jin Y, Yan Y (2023). The effects of short-term exposure to air pollution on mortality in Baotou, China, during 2015–2019. Environmental Geochemistry and Health.

[R94] Luo H, Zhang Q, Niu Y, Kan H, Chen R (2023). Fine particulate matter and cardiorespiratory health in China: a systematic review and meta-analysis of epidemiological studies. Journal of Environmental Sciences.

[R95] Luo Z, Lv Z, Zhao J, Sun H, He T, Yi W, Zhang Z, He K, Liu H (2024). Shipping-related pollution decreased but mortality increased in Chinese port cities. Nature Cities.

[R96] Luo Z, Wang Y, Lv Z, He T, Zhao J, Wang Y, Gao F, Zhang Z, Liu H (2022). Impacts of vehicle emission on air quality and human health in China. Science of The Total Environment.

[R97] Lv Z, Luo Z, Deng F, Wang X, Zhao J, Xu L, He T, Zhang Y, Liu H, He K (2022). Development and application of a multi-scale modeling framework for urban high-resolution NO_2_ pollution mapping. Atmospheric Chemistry and Physics.

[R98] Ma X, Duan H, Zhang H, Liu X, Sun X, Wei J, Zhao M, Xi B (2023). Short-term effects of PM_1_, PM_2.5_, and PM_2.5_ constituents on myocardial infarction mortality in qingdao, China: A time-stratified case-crossover analysis. Atmospheric Environment.

[R99] Ma Y, Chen R, Pan G, Xu X, Song W, Chen B, Kan H (2011). Fine particulate air pollution and daily mortality in Shenyang, China. Science of The Total Environment.

[R100] Miao Y, Ding Y (2020). Indoor environmental quality in existing public buildings in China: Measurement results and retrofitting priorities. Building and Environment.

[R101] Mo S, Hu J, Yu C, Bao J, Shi Z, Zhou P, Yang Z, Luo S, Yin Z, Zhang Y (2023). Short-term effects of fine particulate matter constituents on myocardial infarction death. Journal of Environmental Sciences.

[R102] Morawska L, Allen J, Bahnfleth W, Bennett B, Bluyssen PM, Boerstra A, Buonanno G, Cao J, Dancer SJ, Floto A (2024). Mandating indoor air quality for public buildings. Science.

[R103] Munir MM, Adrian M, Saputra C, Lestari P (2022). Utilizing low-cost mobile monitoring to estimate the PM_2.5_ inhaled dose in urban environment. Aerosol and Air Quality Research.

[R104] Niu Y, Zhou Y, Chen R, Yin P, Meng X, Wang W, Liu C, Ji JS, Qiu Y, Kan H, Zhou M (2022). Long-term exposure to ozone and cardiovascular mortality in China: a nationwide cohort study. The Lancet Planetary Health.

[R105] Orellano P, Reynoso J, Quaranta N, Bardach A, Ciapponi A (2020). Short-term exposure to particulate matter (PM_10_ and PM_2.5_), nitrogen dioxide (NO_2_), and ozone (O_3_) and all-cause and cause-specific mortality: Systematic review and meta-analysis. Environment International.

[R106] Özkaynak H, Baxter LK, Dionisio KL, Burke J (2013). Air pollution exposure prediction approaches used in air pollution epidemiology studies. Journal of Exposure Science & Environmental Epidemiology.

[R107] Peng XW, Yanding, Zhang Xinmin, Tan Haiping, Guo Shu (2023). Associations of ambient PM_2.5_ and O_3_ with human mortality: A time-series study in a city of central China. Journal of Environmental and Occupational Medicine.

[R108] Pu A, Guo Y, Wu C, Ma R, Li R, Li Y, Xiang H, Yan Y (2023). Short-term association between air pollution and hypertension mortality in Wuhan residents. Air Quality, Atmosphere & Health.

[R109] Qian Y, Han M, Jin W, Cai R, Fang B, Yu H, Qian N, Wang C (2015). Association between ambient air pollution and COPD mortality in Shanghai. Journal of Environmental and Occupational Medicine.

[R110] Qian Z, He Q, Lin H-M, Kong L, Zhou D, Liang S, Zhu Z, Liao D, Liu W, Bentley CM (2010). Part 2. Association of daily mortality with ambient air pollution, and effect modification by extremely high temperature in Wuhan, China. Research report (Health Effects Institute).

[R111] Qiu H, Pun VC, Tian L (2018). Short-term effects of fine and coarse particles on deaths in Hong Kong elderly population: An analysis of mortality displacement. Environmental Pollution.

[R112] Qu Y, Pan Y, Niu H, He Y, Li M, Li L, Liu J, Li B (2018). Short-term effects of fine particulate matter on non-accidental and circulatory diseases mortality: a time series study among the elder in Changchun. PLOS ONE.

[R113] Ren X, Mi ZY, Cai T, Nolte CG, Georgopoulos PG (2022). Flexible Bayesian Ensemble Machine Learning Framework for Predicting Local Ozone Concentrations. Environmental Science & Technology.

[R114] Roy A, Mandal M, Das S, Kumar M, Popek R, Awasthi A, Giri BS, Mondal KC, Sarkar A (2024). Non-exhaust particulate pollution in Asian countries: A comprehensive review of sources, composition, and health effects. Environmental Engineering Research.

[R115] Shafran-Nathan R, Yuval Broday DM (2018). Impacts of personal mobility and diurnal concentration variability on exposure misclassification to ambient pollutants. Environmental Science & Technology.

[R116] Shao M, Yu L, Xiao C, Deng J, Yang H, Xu W, Chen Y, Liu X, Ni J, Pan F (2021). Short-term effects of ambient temperature and pollutants on the mortality of respiratory diseases: A time-series analysis in Hefei, China. Ecotoxicology and Environmental Safety.

[R117] Shi Q, Zheng B, Zheng Y, Tong D, Liu Y, Ma H, Hong C, Geng G, Guan D, He K (2022). Co-benefits of CO_2_ emission reduction from China’s clean air actions between 2013-2020. NATURE COMMUNICATIONS.

[R118] Shimada Y, Matsuoka Y (2011). Analysis of indoor PM_2.5_ exposure in Asian countries using time use survey. Science of The Total Environment.

[R119] Song J, Ding Z, Zheng H, Xu Z, Cheng J, Pan R, Yi W, Wei J, Su H (2022). Short-term PM_1_ and PM_2.5_ exposure and asthma mortality in Jiangsu Province, China: What’s the role of neighborhood characteristics?. Ecotoxicology and Environmental Safety.

[R120] Song J, Zhou S, Xu J, Su L (2021). From PM_2.5_ exposure to PM_2.5_ risks of inhaled dose in daily activities: Empirical evidence during workdays from Guangzhou, China. Atmospheric Environment.

[R121] Southerland VA, Brauer M, Mohegh A, Hammer MS, Van Donkelaar A, Martin RV, Apte JS, Anenberg SC (2022). Global urban temporal trends in fine particulate matter (PM_2.5_) and attributable health burdens: estimates from global datasets. The Lancet Planetary Health.

[R122] Stapleton HM, Allen JG, Kelly SM, Konstantinov A, Klosterhaus S, Watkins D, McClean MD, Webster TF (2008). Alternate and new brominated flame retardants detected in U.S. house dust. Environ Sci Technol.

[R123] Sterne JA, Sutton AJ, Ioannidis JP, Terrin N, Jones DR, Lau J, Carpenter J, Rücker G, Harbord RM, Schmid CH (2011). Recommendations for examining and interpreting funnel plot asymmetry in meta-analyses of randomised controlled trials. BMJ.

[R124] Sun HZ, Zhao J, Liu X, Qiu M, Shen H, Guillas S, Giorio C, Staniaszek Z, Yu P, Wan MWL, Chim MM (2023). Antagonism between ambient ozone increase and urbanization-oriented population migration on Chinese cardiopulmonary mortality. THE INNOVATION.

[R125] Sun Y, Zhang Y, Chen C, Sun Q, Wang Y, Du H, Wang J, Zhong Y, Shi W, Li T (2022). Impact of Heavy PM_2.5_ Pollution Events on Mortality in 250 Chinese Counties. Environmental Science & Technology.

[R126] Tao Y, Huang W, Huang X, Zhong L, Lu S-E, Li Y, Dai L, Zhang Y, Zhu T (2012). Estimated acute effects of ambient ozone and nitrogen dioxide on mortality in the Pearl River Delta of southern China. Environmental Health Perspectives.

[R127] Tian Q, Li M, Montgomery S, Fang B, Wang C, Xia T, Cao Y (2020). Short-term associations of fine particulate matter and synoptic weather types with cardiovascular mortality: an ecological time-series study in Shanghai, China. International Journal of Environmental Research and Public Health.

[R128] Wang Y, Du Z, Zhang Y, Chen S, Lin S, Hopke PK, Rich DQ, Zhang K, Romeiko XX, Deng X, Qu Y (2023a). Long-term exposure to particulate matter and COPD mortality: Insights from causal inference methods based on a large population cohort in southern China. Science of The Total Environment.

[R129] Wang Y, Li Q, Luo Z, Zhao J, Lv Z, Deng Q, Liu J, Ezzati M, Baumgartner J, Liu H (2023b). Ultra-high-resolution mapping of ambient fine particulate matter to estimate human exposure in Beijing. Communications Earth & Environment.

[R130] Wang Y, Luo S, Wei J, Yang Z, Hu K, Yao Y, Zhang Y (2023c). Ambient NO_2_ exposure hinders long-term survival of Chinese middle-aged and older adults. Science of The Total Environment.

[R131] Wang Y, Wei J, Zhang Y, Guo T, Chen S, Wu W, Chen S, Li Z, Qu Y, Xiao J, Deng X (2023d). Estimating causal links of long-term exposure to particulate matters with all-cause mortality in South China. Environment International.

[R132] Wang Y, Zhang Z, Luo Z, He T, Liu H, Duan L, Lu K, Liu C, Li X, Wu F, Zhang Y (2022). Ambient air quality baseline and standard formulation methods and their enlightenment for China. Chinese Science Bulletin.

[R133] WHO (2021a). WHO global air quality guidelines.

[R134] WHO (2021b). WHO global air quality guidelines. Particulate matter (PM_25_ and PM_10_), ozone, nitrogen dioxide, sulfur dioxide and carbon monoxide.

[R135] WHO (2022). Fact sheets for ambient (outdoor) air pollution.

[R136] Wong C-M, Ma S, Hedley AJ, Lam T-H (2001). Effect of air pollution on daily mortality in Hong Kong. Environmental Health Perspectives.

[R137] Wong C-M, Vichit-Vadakan N, Kan H, Qian Z (2008). Public Health and Air Pollution in Asia (PAPA): a multicity study of short-term effects of air pollution on mortality. Environmental Health Perspectives.

[R138] Wong CM, Lai HK, Tsang H, Thach TQ, Thomas GN, Lam KBH, Chan KP, Yang L, Lau AK, Ayres JG (2015). Satellite-based estimates of long-term exposure to fine particles and association with mortality in elderly Hong Kong residents. Environmental Health Perspectives.

[R139] Wong TW, Tam W, Yu T, Wong A (2002). Associations between daily mortalities from respiratory and cardiovascular diseases and air pollution in Hong Kong, China. Occupational and environmental medicine.

[R140] Wu H, Lu K, Fu J (2022). A time-series study for effects of ozone on respiratory mortality and cardiovascular mortality in Nanchang, Jiangxi Province, China. FRONTIERS IN PUBLIC HEALTH.

[R141] Wu R, Song X, Chen D, Zhong L, Huang X, Bai Y, Hu W, Ye S, Xu H, Feng B, Wang T (2019). Health benefit of air quality improvement in Guangzhou, China: Results from a long time-series analysis (2006–2016). Environment International.

[R142] Wu R, Zhong L, Huang X, Xu H, Liu S, Feng B, Wang T, Song X, Bai Y, Wu F, Wang X (2018). Temporal variations in ambient particulate matter reduction associated short-term mortality risks in Guangzhou, China: A time-series analysis (2006–2016). Science of The Total Environment.

[R143] Xia Y, Liu Z, Hu B, Rangarajan S, Ah Tse L, Li Y, Wang J, Hu L, Wang Y, Xiang Q, Lin Y (2023). Associations of outdoor fine particulate air pollution and cardiovascular disease: Results from the Prospective Urban and Rural Epidemiology Study in China (PURE-China). Environment International.

[R144] Xiang S, Zhang S, Wang H, Yu YT, Wallington TJ, Shen W, Kirchner U, Deng Y, Tan Q, Zhou Z (2022). Variability of NO_2_/NO*_x_* Ratios in Multiple Microenvironments from On-Road and Near-Roadway Measurements. ACS ES&T Engineering.

[R145] Xiao Q, Geng G, Xue T, Liu S, Cai C, He K, Zhang Q (2021). Tracking PM_2.5_ and O_3_ pollution and the related health burden in China 2013–2020. Environmental Science & Technology.

[R146] Xiong K, Xie X, Mao J, Wang K, Huang L, Li J, Hu J (2023). Improving the accuracy of O_3_ prediction from a chemical transport model with a random forest model in the Yangtze River Delta region, China. Environmental Pollution.

[R147] Xu J, Geng W, Geng X, Cui L, Ding T, Xiao C, Zhang J, Tang J, Zhai J (2020a). Study on the association between ambient air pollution and daily cardiovascular death in Hefei, China. Environmental Science and Pollution Research.

[R148] Xu M, Sbihi H, Pan X, Brauer M (2020b). Modifiers of the effect of short-term variation in PM_2.5_ on mortality in Beijing, China. ENVIRONMENTAL RESEARCH.

[R149] Xu R, Wang Q, Wei J, Lu W, Wang R, Liu T, Wang Y, Fan Z, Li Y, Xu L (2022). Association of short-term exposure to ambient air pollution with mortality from ischemic and hemorrhagic stroke. European Journal of Neurology.

[R150] Yan Y, She L, Guo Y, Zhao Y, Zhang P, Xiang B, Zeng J, Yang M, Wang L (2021). Association between ambient air pollution and mortality from chronic obstructive pulmonary disease in Wuhan, China: a population-based time-series study. Environmental Science and Pollution Research.

[R151] Yang C, Yang H, Guo S, Wang Z, Xu X, Duan X, Kan H (2012). Alternative ozone metrics and daily mortality in Suzhou: The China Air Pollution and Health Effects Study (CAPES). Science of The Total Environment.

[R152] Yang J, Fan X, Zhang H, Zheng W, Ye T (2023). A review on characteristics and mitigation strategies of indoor air quality in underground subway stations. Science of The Total Environment.

[R153] Yang X, Liang F, Li J, Chen J, Liu F, Huang K, Cao J, Chen S, Xiao Q, Liu X (2020). Associations of long-term exposure to ambient PM_2.5_ with mortality in Chinese adults: A pooled analysis of cohorts in the China-PAR project. Environment International.

[R154] Yang Y, Li R, Li W, Wang M, Cao Y, Wu Z, Xu Q (2013). The association between ambient air pollution and daily mortality in Beijing after the 2008 olympics: a time series study. PLOS ONE.

[R155] Yang Y, Ruan Z, Wang X, Yang Y, Mason TG, Lin H, Tian L (2019). Short-term and long-term exposures to fine particulate matter constituents and health: a systematic review and meta-analysis. Environmental Pollution.

[R156] Yang Y, Tang R, Qiu H, Lai P-C, Wong P, Thach T-Q, Allen R, Brauer M, Tian L, Barratt B (2018a). Long term exposure to air pollution and mortality in an elderly cohort in Hong Kong. Environment International.

[R157] Yang Z, Lin T, Cheng H, Wang Z, Cheng Z, Lyu X, Zhan L, Zhang G (2018b). Concentration and Seasonal Variation of Halogenated Flame Retardants at a CAWNET Background Site in Central China. Aerosol and Air Quality Research.

[R158] Yin P, Brauer M, Cohen A, Burnett RT, Liu J, Liu Y, Liang R, Wang W, Qi J, Wang L (2017a). Long-term fine particulate matter exposure and nonaccidental and cause-specific mortality in a large national cohort of Chinese men. Environmental Health Perspectives.

[R159] Yin P, Chen R, Wang L, Meng X, Liu C, Niu Y, Lin Z, Liu Y, Liu J, Qi J (2017b). Ambient ozone pollution and daily mortality: a nationwide study in 272 Chinese cities. Environmental Health Perspectives.

[R160] Yorifuji T, Kashima S (2020). Long-term exposure to nitrogen dioxide and natural-cause and cause-specific mortality in Japan. Science of The Total Environment.

[R161] Yu Y, Yao S, Dong H, Wang L, Wang C, Ji X, Ji M, Yao X, Zhang Z (2019). Association between short-term exposure to particulate matter air pollution and cause-specific mortality in Changzhou, China. ENVIRONMENTAL RESEARCH.

[R162] Yuan Y, Wang K, Sun HZ, Zhan Y, Yang Z, Hu K, Zhang Y (2023). Excess mortality associated with high ozone exposure: A national cohort study in China. Environmental Science and Ecotechnology.

[R163] Zhai S, Zeng J, Zhang Y, Huang J, Li X, Wang W, Zhang T, Deng Y, Yin F, Ma Y (2023). Combined health effects of PM_2.5_ components on respiratory mortality in short-term exposure using BKMR: A case study in Sichuan, China. Science of The Total Environment.

[R164] Zhang F, Liu X, Zhou L, Yu Y, Wang L, Lu J, Wang W, Krafft T (2016). Spatiotemporal patterns of particulate matter (PM) and associations between PM and mortality in Shenzhen, China. BMC PUBLIC HEALTH.

[R165] Zhang J, Liu Y, Cui L-l, Liu S-q, Yin X-x, Li H-c (2017). Ambient air pollution, smog episodes and mortality in Jinan, China. Scientific Reports.

[R166] Zhang KJ, Wu, Yao Qingbin, Han Xiaoliang, Li Xiaoqin, Meng E (2019). Time series analysis of the relationship between air fine particulate matter and resident deaths in Yangzhou City. Journal of Nantong University (Medical Edition).

[R167] Zhang P, Dong G, Sun B, Zhang L, Chen X, Ma N, Yu F, Guo H, Huang H, Lee YL (2011). Long-term exposure to ambient air pollution and mortality due to cardiovascular disease and cerebrovascular disease in Shenyang, China. PLOS ONE.

[R168] Zhang WL, Lijian (2023). Time series analysis on the effect between atmospheric PM_2.5_ pollution and deaths from cardiovascular diseases among residents in Yangquan City, 2019-2020. Practical Preventive Medicine.

[R169] Zhang X, Xu W, Zhang G, Lin W, Zhao H, Ren S, Zhou G, Chen J, Xu X (2022a). Discrepancies in ozone levels and temporal variations between urban and rural North China Plain: Possible implications for agricultural impact assessment across China. Elem Sci Anth.

[R170] Zhang Y, Li Z, Wei J, Zhan Y, Liu L, Yang Z, Zhang Y, Liu R, Ma Z (2022b). Long-term exposure to ambient NO_2_ and adult mortality: A nationwide cohort study in China. Journal of Advanced Research.

[R171] Zhang Y, Wang Y, Du Z, Chen S, Qu Y, Hao C, Ju X, Lin Z, Wu W, Xiao J, Chen X (2023a). Potential causal links between long-term ambient particulate matter exposure and cardiovascular mortality: New evidence from a large community-based cohort in South China. Ecotoxicology and Environmental Safety.

[R172] Zhang Y, Xiang Q, Yu C, Yang Z (2020). Asthma mortality is triggered by short-term exposures to ambient air pollutants: Evidence from a Chinese urban population. Atmospheric Environment.

[R173] Zhang Y, Yin Z, Li S, Zhang JJ, Sun HZ, Liu K, Shirai K, Hu K, Qiu C, Liu X (2023b). Ambient PM_2.5_, ozone and mortality in Chinese older adults: A nationwide cohort analysis (2005–2018). Journal of Hazardous Materials.

[R174] Zhang Z, Wang J, Kwong JC, Burnett RT, van Donkelaar A, Hystad P, Martin RV, Bai L, McLaughlin J, Chen H (2021). Long-term exposure to air pollution and mortality in a prospective cohort: the Ontario Health Study. Environment International.

[R175] Zhong P, Huang S, Zhang X, Wu S, Zhu Y, Li Y, Ma L (2018). Individual-level modifiers of the acute effects of air pollution on mortality in Wuhan, China. Global health research and policy.

[R176] Zhou P, Hu J, Yu C, Bao J, Luo S, Shi Z, Yuan Y, Mo S, Yin Z, Zhang Y (2022). Short-term exposure to fine particulate matter constituents and mortality: case-crossover evidence from 32 counties in China. Science China Life Sciences.

[R177] Zhu Y, Huang L, Li J, Ying Q, Zhang H, Liu X, Liao H, Li N, Liu Z, Mao Y (2018). Sources of particulate matter in China: Insights from source apportionment studies published in 1987–2017. Environment International.

[R178] Zou B, You J, Lin Y, Duan X, Zhao X, Fang X, Campen MJ, Li S (2019). Air pollution intervention and life-saving effect in China. Environment International.

