# Supplementary material for: Key Factors in Epidemiological Exposure and Insights for Environmental Management: Evidence from Meta-analysis
Source: Environ Pollut. Author manuscript; Available in PMC 2024 Dec 1. (PMC7616677; doi:10.1016/j.envpol.2024.124991)
Supplement: Supplementary Material [file EMS198683-supplement-Supplementary_Material.zip › 1-s2.0-S0269749124017068-mmc1.docx]

**Appendix for Key Factors in Epidemiological Exposure and Insights for Environmental Management: Evidence from Meta-analysis**

*Yongyue Wang^1^, Jie Chang^2,3^, Piaopiao Hu^3^, Chun Deng^1^, Zhenyu Luo^1^, Junchao Zhao^1^, Zhining Zhang^1^, Wen Yi^1^, Guanlin Zhu^1^, Guangjie Zheng^1^, Shuxiao Wang^1^, Kebin He^1^, Jing Liu^3^, Huan Liu^*,1^*

^1^ State Environmental Protection Key Laboratory of Sources and Control of Air Pollution Complex, State Key Joint Laboratory of Environmental Simulation and Pollution Control, School of Environment, Tsinghua University, Beijing 100084, China;

^2^ National Center for Neurological Disorders, Xuanwu Hospital, Capital Medical University, Beijing 100084, China;

^3^ Centre for Clinical and Epidemiologic Research, Beijing An Zhen Hospital, Capital Medical University, Beijing Institute of Heart, Lung and Blood Vessel Diseases, Beijing 100029, China.

**Correspondence to:* Huan Liu (liu_env@tsinghua.edu.cn)

**Contents**

Appendix 1: Searching strategies for literature identification

Appendix 2: Confounder selection with directed acyclic graph

Appendix 3: Details of certainty assessment of evidence

Appendix 4: Literature excluded in the eligibility process

Appendix 5: Lists of studies included in long-term exposure effect meta-analysis

Appendix 6: Lists of studies included in short-term exposure effect meta-analysis

Appendix 7: Summary of pollutant level distribution among the included literature

Appendix 8: Details of the meta-analysis of the long- and short-term exposure effect of PM_2.5_, NO_2_ and O_3_ on cause-specific mortality

Appendix 9: Short- and long-term exposure effects of PM_2.5_, NO_2_, and O_3_ on cause-specific mortality in age and gender sub-groups

Appendix 10: Details of the ROB assessment

Appendix 11: Results of the symmetry assessment of the funnel plot

Appendix 12: Results of the meta-regression analysis

Appendix 13: Sensitivity Analysis

Appendix 14: Results of the certainty assessment

**Appendix 1: Searching strategies for literature identification**

1. **Identification of the epidemiological studies**

Web of Science:

TS = (((air pollution) OR (air pollutant) OR (PM2.5) OR (fine particle) OR (fine particulate) OR (particulate matter) OR (particle matter) OR (respirable particles) OR (suspended particles) OR (nitrogen dioxide) OR (NO2) OR (NOx) OR (O3) OR (ozone)) AND ((mortality) OR (death) OR (dying) OR (fatal) OR (fatality) OR (lethal)) AND ((cohort) OR (time series) OR (time-series) OR (case crossover) OR (case cross over) OR (cross-over) OR (longitudinal)) AND ((China) OR (Chinese)))

PubMed:

("air pollution"[Title/Abstract] OR "air pollutant"[Title/Abstract] OR "PM2.5"[Title/Abstract] OR "fine particle"[Title/Abstract] OR "fine particulate"[Title/Abstract] OR "particulate matter"[Title/Abstract] OR "particle matter"[Title/Abstract] OR "respirable particles"[Title/Abstract] OR "suspended particles"[Title/Abstract] OR "nitrogen dioxide"[Title/Abstract] OR "NO2"[Title/Abstract] OR "NOx"[Title/Abstract] OR "O3"[Title/Abstract] OR "ozone"[Title/Abstract]) AND ("mortality"[Title/Abstract] OR "death"[Title/Abstract] OR "dying"[Title/Abstract] OR "fatal"[Title/Abstract] OR "fatality"[Title/Abstract] OR "lethal"[Title/Abstract]) AND ("cohort"[Title/Abstract] OR "time-series"[Title/Abstract] OR "time-series"[Title/Abstract] OR "case crossover"[Title/Abstract] OR "case cross over"[Title/Abstract] OR "cross-over"[Title/Abstract] OR "longitudinal"[Title/Abstract]) AND ("China"[Title/Abstract] OR "Chinese"[Title/Abstract])

Scopus:

TITLE-ABS-KEY(((air pollution) OR (air pollutant) OR (PM2.5) OR (fine) OR (particulate) OR (particle) OR (NO2) OR (O3)) AND ((cohort) OR (time series) OR (cross over)) AND ((mortality) OR (death) OR (fatal)) AND ((China) OR (Chinese)))

CNKI (searching in Chinese):

SU = ((‘大气污染’+’空气污染’+’PM2.5’+’颗粒物’+’O3’+臭氧+‘NO2’+‘二氧化氮’)*((‘呼吸’*’死亡’)+’死亡’)*(‘队列’+‘时间序列’+‘交叉’))

1. **Identification and scope summary of the existing systematic reviews and meta-analyses**

Web of science:

TS = (((review) OR (meta)) AND ((air pollution) OR (air pollutant) OR (nitrogen dioxide) OR (NO2) OR (NOx) OR (O3) OR (ozone)) AND ((mortality) OR (death) OR (dying) OR (fatal) OR (fatality) OR (lethal)) AND ((cohort) OR (time series) OR (time-series) OR (case crossover) OR (case cross over) OR (cross-over) OR (longitudinal)) AND ((China) OR (Chinese)))

PubMed:

("air pollution"[Title/Abstract] OR "air pollutant"[Title/Abstract] OR "PM2.5"[Title/Abstract] OR "fine particle"[Title/Abstract] OR "fine particulate"[Title/Abstract] OR "particulate matter"[Title/Abstract] OR "particle matter"[Title/Abstract] OR "respirable particles"[Title/Abstract] OR "suspended particles"[Title/Abstract] OR "nitrogen dioxide"[Title/Abstract] OR "NO2"[Title/Abstract] OR "NOx"[Title/Abstract] OR "O3"[Title/Abstract] OR "ozone"[Title/Abstract]) AND ("mortality"[Title/Abstract] OR "death"[Title/Abstract] OR "dying"[Title/Abstract] OR "fatal"[Title/Abstract] OR "fatality"[Title/Abstract] OR "lethal"[Title/Abstract]) AND ("cohort"[Title/Abstract] OR "time-series"[Title/Abstract] OR "time-series"[Title/Abstract] OR "case crossover"[Title/Abstract] OR "case cross over"[Title/Abstract] OR "cross-over"[Title/Abstract] OR "longitudinal"[Title/Abstract]) AND ("China"[Title/Abstract] OR "Chinese"[Title/Abstract]) AND ("review"[Title/Abstract] OR "meta"[Title/Abstract])

**Summary for the identified meta-analysis studies:**

The identification showed the same result both in Web of science and the PubMed. A total of 65 meta-analyses and systematic reviews were retrieved, with 26 specifically focusing on China. Through the retrieval of long-term and short-term epidemiological studies in our study, a notable trend emerged indicating a rapid increase in evidence of exposure in China since 2017, marking it as a pivotal time point. Literature published before this timeframe may lack adequate representation of the latest evidence. Among the newly published literature, 14 papers emerged post-2017, with 13 in the last three years. However, only 3 papers delved into long-term exposure, covering topics such as the impact of PM_2.5_ on the mortality risk of LC and COPD, the impact of PM_2.5_ and ozone exposure on preterm birth, and the impact of PM_2.5_ on cause-specific mortality and morbidity effects. The remaining literature predominantly focused on short-term exposure. Regarding pollutants, the primary focus was on the exposure effects of PM_2.5_ and O_3_ (with 1 study involving NO_2_ and SO_2_, and 1 study involving CO). Subgroup analyses mainly concentrated on age, gender, different regions, and temperature. Notably, only 1 study, which analyzed the short-term effects of ozone on mortality, examined the differences in effects before and after the implementation of pollution policies in 2013. Intriguingly, no literature critically examined the accuracy of pollution exposure assessment methods and their impact on the quantification of results. This comprehensive meta-analysis retrieval underscores that this paper, building upon existing studies, enriches the quantitative summary by considering pollution exposure duration, types of pollutants, pollution levels, and the accuracy of exposure assessment methods. All in all, the research objectives and scope of this study do not overlap with existing research.

**Appendix 2: Confounders selection with directed acyclic graph**

The directed acyclic graph


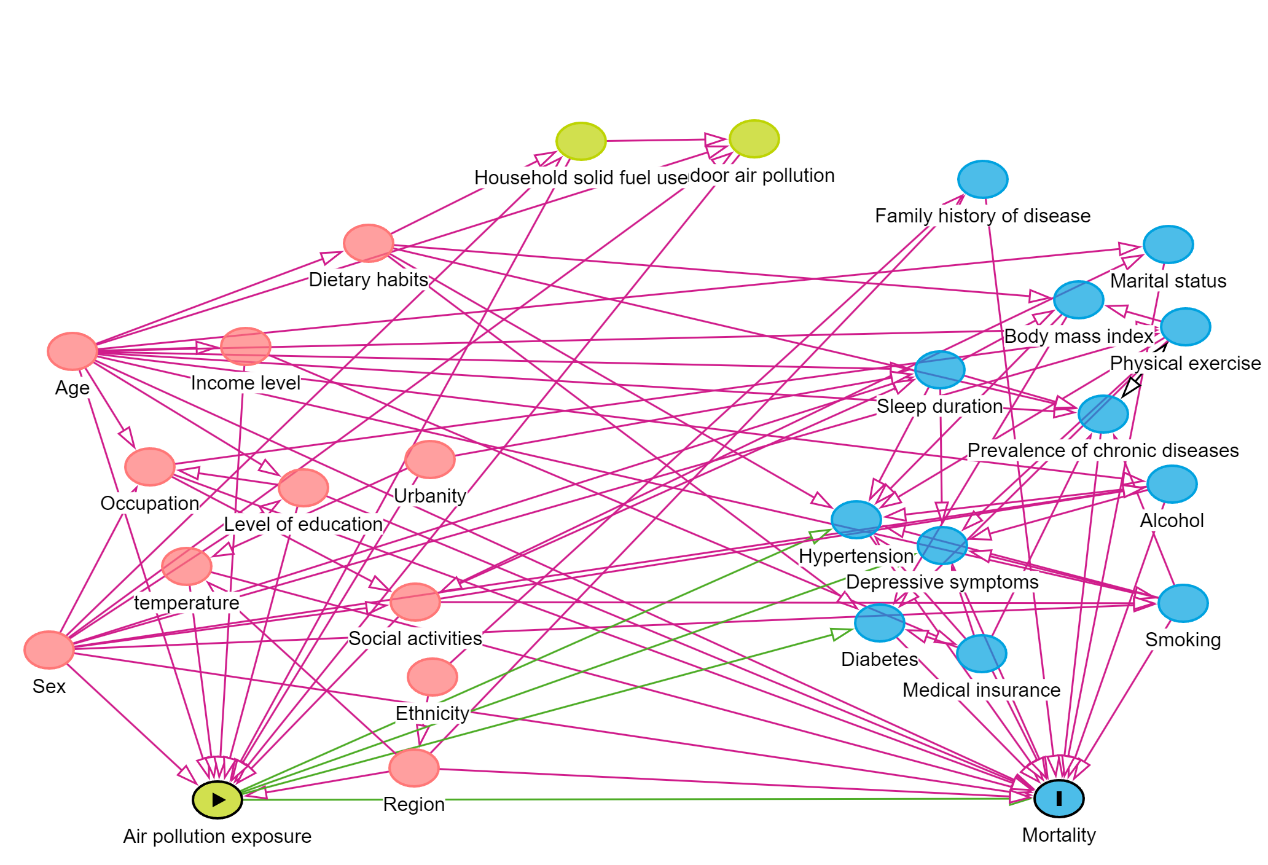


The directed acyclic graph indicates that the appropriate selection is to minimally include age, gender, occupation, income level, temperature, level of education, dietary habits, urbanity, social activities, ethnicity, and region in the confounding factor adjustment model.

**Appendix 3: Details of certainty assessment of evidence** (2020)

- - - 1. **Limitations in studies:**

Downgrade if the sensitivity analysis reveals a statistically significant difference between studies with high RoB and those with moderate or low RoB.

- - - 1. **Indirectness:**

Downgrade if the research questions in the included studies do not reflect the original question.

- - - 1. **Inconsistency:**

Downgrade if significant heterogeneity is detected, meaning that the prediction intervals contain unity and are more than twice the confidence interval of the random-effects meta-analysis.

- - - 1. **Imprecision:**

For cohort studies, if the number of death cases used to calculate the summary effect size is below 940,000, the evidence grade will be downgraded.

For time-series and case-crossover studies, where the number of mortality cases may be much less than in cohort studies, the threshold for determining the evidence grade may be too strict. According to reasoning in the literature (Orellano et al., 2020), if the number of events is sufficient for a given study to detect a significant effect size, the same number is sufficient for meta-analysis. As a reference, for time-series and case-crossover studies, if the number of mortality cases used to calculate the summary effect size is below 100,000 or fewer, the evidence grade will be downgraded.

- - - 1. **Publication bias:**

Downgrade if publication bias is visually detected through a funnel plot inspection or detected through meta-regression tests. However, if the analysis comparing studies from multiple cities with single-city studies does not show a statistical difference, the evidence will not be downgraded.

- - - 1. **Large effect size:**

The degree of interference of confounding factors on the results can be measured by the calculation using the E-value, thereby determining the reliability of the pooled effects (Orellano et al., 2020; VanderWeele and Ding, 2017). If the confounding factors are indeed closely related to both exposure and outcome, unmeasured confounding could explain the observed pooled relative risk. In other words, unmeasured confounding factors could significantly impact the observed results, introducing limitations and biases to the existing findings. If it is determined that unmeasured confounding factors are unlikely to result in a relative risk as high as the E-value, it can be concluded that unmeasured confounding factors are unlikely to explain the observed relative risk, and the existing results are relatively robust and reliable. The E-value can be calculated using the following equation:

$$E-value=\mathrm{RR}_{\mathrm{observed}}+\sqrt{\mathrm{RR}_{\mathrm{observed}}\times(\mathrm{RR}_{\mathrm{observed}}-1)}$$

The criteria for the similarity judgement is: when the observed RR is higher than the lower confidence limit of the E-value, evidence certainty will not be upgraded. Otherwise, the evidence will be upgraded.

- - - 1. **Confounding:**

Not be upgraded when potential confounders may change the RR in two directions; upgrade in the opposite case.

- - - 1. **Concentration-response gradient:**

Upgrade when the estimated exposure-response relationship is statistically significant.

**Appendix 4: Literature excluded in the eligibility process**

There were 3572 studies identified from several databases, together with 1578 out of 37104 cited studies. A total of 2797 duplicates were excluded, and 2110 were excluded by screening the title and abstract. The remaining 243 studies were all included in the systematic review. Among the remaining, 15 were excluded after an in-depth review of the full text for the potentially high ROB risks (Huang et al., 2020; Kan et al., 2003a; Leepe et al., 2019; Li et al., 2018c; Li et al., 2019b; Liang et al., 2018a; Liu et al., 2012; Liu et al., 2015; Luo et al., 2020; Ren, 2007; Wenghaerbai et al., 2018; Yuan et al., 2022; Zhang et al., 2014; Zhang and Guo, 2010; Zhang and Meng, 2011), and 228 remained studies. Among them, there were 9 studies crossed out for the cohort database overlap (Chen et al., 2023b; Dong et al., 2012; Hu et al., 2023; Ji et al., 2020a; Wong et al., 2015; Yang et al., 2021; Yao et al., 2021; Zhang et al., 2011b; Zhang, 2021), while 105 for the consideration of spatiotemporal representativeness to prevent overlap in the study period as well as the study area coverage for time-series studies and case-crossover studies (14 nationwide studies (Ban et al., 2021; Chen et al., 2019a; Chen et al., 2018a; Chen et al., 2013; He et al., 2020; Liang et al., 2017; Liu et al., 2019a; Ma et al., 2017; Meng et al., 2013; Shi et al., 2020; Tian et al., 2022; Tian et al., 2020a; Zhang et al., 2020a; Zhao et al., 2022), 35 provincial studies (Chen et al., 2008; Chen et al., 2017a; Chen et al., 2021e; Fang et al., 2022; Fang et al., 2017; Fang et al., 2019; Gong et al., 2019b; Gu and Chen, 2017; Guo et al., 2010; Kan and Chen, 2003; Kan et al., 2003b, 2004; Kan et al., 2008; Li et al., 2013b; Li et al., 2018d; Li et al., 2015c; Liang et al., 2018b; Lin et al., 2016a; Lin et al., 2017; Luo et al., 2016a; Luo et al., 2016b; Qian et al., 2019; Qian et al., 2013; Su et al., 2015; Tang et al., 2006; Wang et al., 2020a; Wang et al., 2019c; Xie et al., 2015; Yang et al., 2008; Yang et al., 2015; Yu et al., 2006; Zhang et al., 2011a; Zhang et al., 2019; Zhang et al., 2018; Zhang et al., 2006), 56 city-level studies (Cai et al., 2019; Cai et al., 2018; Cao et al., 2018; Chen et al., 2021a; Chen et al., 2020; Chen et al., 2010; CHEN et al., 2019d; Chen et al., 2019e; Cheng et al., 2019; Dong et al., 2018; Fan et al., 2023; Feng et al., 2022; Ge et al., 2015; Guo et al., 2018; Guo et al., 2017; Hong et al., 2020; Huang et al., 2012; Ji et al., 2018; Jiang et al., 2022; Jie et al., 2018; Lei et al., 2019; Li et al., 2018a; Li et al., 2021; Li et al., 2015b; Lin et al., 2016c; Lin et al., 2016d; Liu et al., 2019b; Liu et al., 2019c; Liu et al., 2013; Mokoena et al., 2019; Pan et al., 2019; Qi et al., 2018; Qian et al., 2017; Qian et al., 2007; Shuang et al., 2013; Sui et al., 2021; Wang et al., 2020b; Wu et al., 2019b; Wu et al., 2020; Xu et al., 2019; Xue et al., 2018; Yang et al., 2012a; Yin et al., 2019; Yu et al., 2012; Yu et al., 2017; Zai-sheng et al., 2020; Zeng et al., 2017; Zhang et al., 2017a; Zhang, 2019a; Zhang et al., 2022b; Zhang et al., 2022c; Zhang et al., 2015; Zhou et al., 2021; Zhou et al., 2018; Zhu et al., 2021; Zhu et al., 2019). There were 10 studies focusing on the specific group (e.g., patients) instead of the ordinary population (Chen et al., 2019b; Ge et al., 2022; He et al., 2022; Peng et al., 2017; Song et al., 2019; Wang et al., 2019a; Wang et al., 2023a; Wang et al., 2023b; Xu et al., 2021; Zhao et al., 2023) crossed out. Other exclusions were 7 for effects or 95% CI unavailable (Li and Peng, 2010; Sun et al., 2020; Sun et al., 2019; Tong et al., 2018; Wang et al., 2017; Wang et al., 2019b; Yin et al., 2012), and 2 for no full text (Lee et al., 2014; Yin et al., 2015).

**Appendix 5: Lists of studies included in long-term exposure effect meta-analysis**

1. Long-term exposure effect of PM_2.5_ to all-cause mortality

| **Authors** | **Year** | **Study design** | **Database Name** | **Study Area** | **Start Year** | **End Year** | **Age** | **Gender** | **Adopted Models** | **Pollutants** | **Exposure Assessment Method** | **Resolution** | **Disease Coding** | **Cite** |
| --- | --- | --- | --- | --- | --- | --- | --- | --- | --- | --- | --- | --- | --- | --- |
| Yin et al. | 2017 | Cohort | DSPs | China | 1990 | 2005 | >40 | All | Cox | PM_2.5_ | Multi-source fusing | county level | ICD-9 | (Yin et al., 2017a) |
| Yang et al. | 2018 | Cohort | EHC | Hong Kong | 1998 | 2011 | >65 | All | Cox | PM_2.5_, BC, NO_2_ | LUR | street level | ICD-10 (A00-R99) | (Huang et al., 2023) |
| Yang et al. | 2020 | Cohort | China-PAR | China | 1992 | 2015 | >18 | All | Cox | PM_2.5_ | Machine learning | 1 km | NA | (Yang et al., 2020) |
| Chen et al. | 2021 | Cohort | RDCS | Zhejiang | 2006 | 2018 | >18 | All | Cox | PM_2.5_ and components | Remote prediction model | 1 km | ICD-10 (A00-R99) | (Chen et al., 2021c) |
| Liu et al. | 2022 | Cohort | CFPS | China | 2010 | 2016 | All | All | Cox | PM_2.5_ and components | Multi-source fusing | county level | NA | (Liu et al., 2022a) |
| Huang et al. | 2023 | Cohort | CDC | Northern China | 1998 | 2019 | >18 | All | Cox | PM_2.5_, PM_10,_ SO_2_, NO_2_ | Multi-source fusing | 10 km | ICD-10 (A00-R99) | (Huang et al., 2023) |
| Zhang et al. | 2023 | Cohort | CLHLS | China | 2005 | 2018 | >65 | All | Cox | PM_2._5, O_3_ | TAP | 1 km | NA | (Zhang et al., 2023b) |
| Wang et al. | 2023 | Cohort | MPSR | China | 2009 | 2015 | >18 | All | Cox | PM_10_, PM_2.5_, PM_1_ | CHAP | 1 km | NA | (Wang et al., 2023e) |
| Xia et al. | 2023 | Cohort | PURE-China | China | 2005 | 2009 | >35 | All | Cox | PM_2.5_ | Multi-source fusing | 1 km | ICD-10 | (Xia et al., 2023) |

1. Long-term exposure effect of PM_2.5_ to cardiovascular mortality

| **Authors** | **Year** | **Study design** | **Database Name** | **Study Area** | **Start Year** | **End Year** | **Age** | **Gender** | **Adopted Models** | **Pollutants** | **Exposure Assessment Method** | **Resolution** | **Disease Coding** | **Cite** |
| --- | --- | --- | --- | --- | --- | --- | --- | --- | --- | --- | --- | --- | --- | --- |
| Yin et al. | 2017 | Cohort | DSPs | China | 1990 | 2005 | >40 | All | Cox | PM_2.5_ | Multi-source fusing | county level | ICD-9 (390-414,420-459) | (Yin et al., 2017a) |
| Yang et al. | 2018 | Cohort | EHC | Hong Kong | 1998 | 2011 | >65 | All | Cox | PM_2.5_, BC, NO2 | LUR | street level | ICD-10 (I00-I99) | (Yang et al., 2018) |
| Liang et al. | 2020 | Cohort | China-PAR | China | 1992 | 2008 | >18 | All | Cox | PM_2.5_ | Remote, observation fusing | 1 km | ICD-10 (I00-I99) | (Liang et al., 2020) |
| Chen et al. | 2021 | Cohort | RDCS | Zhejiang | 2006 | 2018 | >18 | All | Cox | PM_2.5_ and components | Remote prediction model | 1 km | ICD-10 (I00-I99) | (Chen et al., 2021c) |
| Liang et al. | 2022 | Cohort | CCDRFS | China | 2010 | 2017 | >18 | All | Cox | PM_2.5_ | Multi-source fusing | 1 km | ICD-10 (I00-I99) | (Liang et al., 2022) |
| Huang et al. | 2023 | Cohort | CDC | Northern China | 1998 | 2019 | >18 | All | Cox | PM_2.5_, PM_10_, SO_2_, NO_2_ | Multi-source fusing | 10 km | ICD-10 (I00-I70) | (Huang et al., 2023) |
| Zhang et al. | 2023 | Cohort | MPSR | Guangzhou | 2009 | 2015 | >18 | All | Cox | PM_2.5_, PM_10_, PM_10-2.5_ | Multi-source fusing | 1 km | ICD-10 (I00-I99) | (Zhang et al., 2023a) |
| Xia et al. | 2023 | Cohort | PURE-China | China | 2005 | 2009 | >35 | All | Cox | PM_2.5_ | Multi-source fusing | 1 km | ICD-10 (I21-I22, I60-I64, I69, I50, I01, I05- I09, I26, I30-I38, I40, I44-I45, I47-I49, I71- I72, I97, Q20-Q28) | (Xia et al., 2023) |

1. Long-term exposure effect of PM_2.5_ to respiratory mortality

| **Authors** | **Year** | **Study design** | **Database Name** | **Study Area** | **Start Year** | **End Year** | **Age** | **Gender** | **Adopted Models** | **Pollutants** | **Exposure Assessment Method** | **Resolution** | **Disease Coding** | **Cite** |
| --- | --- | --- | --- | --- | --- | --- | --- | --- | --- | --- | --- | --- | --- | --- |
| Yin et al. | 2017 | Cohort | DSPs | China | 1990 | 2005 | >40 | Male | Cox | PM_2.5_ | Multi-source fusing | county level | ICD-9 (490-496) | (Yin et al., 2017a) |
| Yang et al. | 2018 | Cohort | EHC | Hong Kong | 1998 | 2011 | >65 | All | Cox | PM_2.5_, BC, NO_2_ | LUR | street level | ICD-10 (J00-J47,J80-J99) | (Yang et al., 2018) |
| Chen et al. | 2021 | Cohort | RDCS | Zhejiang | 2006 | 2018 | >18 | All | Cox | PM_2.5_ and components | Remote prediction model | 1 km | ICD-10 (J00-J99) | (Chen et al., 2021c) |
| Guo et al. | 2022 | Cohort | MJ | Taiwan | 2001 | 2019 | >18 | All | Cox | PM_2.5_ | Remote, observation fusing | NA | ICD-9/10 (J12-J18) | (Guo et al., 2022) |
| Huang et al. | 2023 | Cohort | CDC | Northern China | 1998 | 2019 | >18 | All | Cox | PM_2.5_, PM_10_, SO_2_, NO_2_ | Multi-source fusing | 10 km | ICD-10 (J00-J99) | (Huang et al., 2023) |
| Wang et al. | 2023 | Cohort | MPSR | Guangzhou | 2009 | 2015 | >18 | All | Cox | PM_2.5_ | Multi-source fusing | 1 km | ICD-10 (J40-J44,J47) | (Wang et al., 2023c) |

1. Long-term exposure effect of NO_2_ to all-cause mortality

| **Authors** | **Year** | **Study design** | **Database Name** | **Study Area** | **Start Year** | **End Year** | **Age** | **Gender** | **Adopted Models** | **Pollutants** | **Exposure Assessment Method** | **Resolution** | **Disease Coding** | **Cite** |
| --- | --- | --- | --- | --- | --- | --- | --- | --- | --- | --- | --- | --- | --- | --- |
| Cao et al. | 2011 | Cohort | CNHFS | China | 1991 | 2000 | >40 | All | Cox | TSP, SO_2_, NO_2_ | Station average | city level | ICD-9 | (Cao et al., 2011) |
| Yang et al. | 2018 | Cohort | EHC | Hong Kong | 1998 | 2011 | >65 | All | Cox | PM_2.5_, BC, NO_2_ | LUR | street level | ICD-10 (A00-R99) | (Yang et al., 2018) |
| Zhang et al. | 2022 | Cohort | CFPS | China | 2010 | 2020 | >18 | All | Cox | NO_2_ | CHAP | 25 km | NA | (Zhang et al., 2022a) |
| Ji et al. | 2022 | Cohort | CLHLS | China | 2008 | 2018 | >65 | All | Cox | PM_2.5_, NO_2_ | Multi-source fusing | 1 km | NA | (Ji et al., 2022) |
| Huang et al. | 2023 | Cohort | CDC | Northern China | 1998 | 2019 | >18 | All | Cox | PM_2.5_, PM_10_, SO_2_, NO_2_ | Multi-source fusing | 10 km | ICD-10 (A00-R99) | (Huang et al., 2023) |
| Wang et al. | 2023 | Cohort | CHARLS | China | 2011 | 2018 | >45 | All | Cox | NO_2_ | CHAP | city level | NA | (Wang et al., 2023d) |

1. Long-term exposure effect of NO_2_ to cardiovascular mortality

| **Authors** | **Year** | **Study design** | **Database Name** | **Study Area** | **Start Year** | **End Year** | **Age** | **Gender** | **Adopted Models** | **Pollutants** | **Exposure Assessment Method** | **Resolution** | **Disease Coding** | **Cite** |
| --- | --- | --- | --- | --- | --- | --- | --- | --- | --- | --- | --- | --- | --- | --- |
| Cao et al. | 2011 | Cohort | CNHFS | China | 1991 | 2000 | >40 | All | Cox | TSP, SO_2_, NO_2_ | Station average | city level | ICD-9 | (Cao et al., 2011) |
| Yang et al. | 2018 | Cohort | EHC | Hong Kong | 1998 | 2011 | >65 | All | Cox | PM_2.5_, BC, NO_2_ | LUR | street level | ICD-10 (I00-I99) | (Yang et al., 2018) |
| Huang et al. | 2023 | Cohort | CDC | Northern China | 1998 | 2019 | >18 | All | Cox | PM_2.5_, PM_10_, SO_2_, NO_2_ | Multi-source fusing | 10 km | ICD-10 (I00-I70) | (Huang et al., 2023) |

1. Long-term exposure effect of NO_2_ to respiratory mortality

| **Authors** | **Year** | **Study design** | **Database Name** | **Study Area** | **Start Year** | **End Year** | **Age** | **Gender** | **Adopted Models** | **Pollutants** | **Exposure Assessment Method** | **Resolution** | **Disease Coding** | **Cite** |
| --- | --- | --- | --- | --- | --- | --- | --- | --- | --- | --- | --- | --- | --- | --- |
| Cao et al. | 2011 | Cohort | CNHFS | China | 1991 | 2000 | >40 | All | Cox | TSP, SO_2_, NO_2_ | Station average | city level | ICD-9 | (Cao et al., 2011) |
| Yang et al. | 2018 | Cohort | EHC | Hong Kong | 1998 | 2011 | >65 | All | Cox | PM_2.5_, BC, NO_2_ | LUR | street level | ICD-10 (J00-J47,J80-J99) | (Yang et al., 2018) |
| Huang et al. | 2023 | Cohort | CDC | Northern China | 1998 | 2019 | >18 | All | Cox | PM_2.5_, PM_10_, SO_2_, NO_2_ | Multi-source fusing | 10 km | ICD-10 (J00-J99) | (Huang et al., 2023) |

1. Long-term exposure effect of O_3_ to all-cause mortality

| **Authors** | **Year** | **Study design** | **Database Name** | **Study Area** | **Start Year** | **End Year** | **Age** | **Gender** | **Adopted Models** | **Pollutants** | **Exposure Assessment Method** | **Resolution** | **Disease Coding** | **Cite** |
| --- | --- | --- | --- | --- | --- | --- | --- | --- | --- | --- | --- | --- | --- | --- |
| Zhang et al. | 2023 | Cohort | CLHLS | China | 2005 | 2018 | >65 | All | DAG-based Cox | PM_2.5_, O_3_ | GBD-2019 | 10 km | NA | (Zhang et al., 2023b) |
| Yuan et al. | 2023 | Cohort | CHARLS | China | 2011 | 2018 | >45 | All | Cox | O_3_ | GBD-2019 with observation fusing | city level | NA | (Yuan et al., 2023) |

1. Long-term exposure effect of O_3_ to cardiovascular mortality

| **Authors** | **Year** | **Study design** | **Database Name** | **Study Area** | **Start Year** | **End Year** | **Age** | **Gender** | **Adopted Models** | **Pollutants** | **Exposure Assessment Method** | **Resolution** | **Disease Coding** | **Cite** |
| --- | --- | --- | --- | --- | --- | --- | --- | --- | --- | --- | --- | --- | --- | --- |
| Liu et al. | 2022 | Cohort | CHERRY | China | 2009 | 2017 | >18 | All | Cox | O_3_ | Multi-source fusing | 1 km | ICD-10 (I00-I99) | (Liu et al., 2022b) |
| Niu et al. | 2022 | Cohort | CCDRFS | China | 2010 | 2018 | >18 | All | Cox | O_3_ | Multi-source fusing | 1 km | ICD-10 (I00-I99) | (Niu et al., 2022) |

**Appendix 6: Lists of studies included in short-term exposure effect meta-analysis**

1. Short-term exposure effect of PM_2.5_ to all-cause mortality

| **Authors** | **Year** | **Study design** | **Study Area** | **Area Note** | **Start Year** | **End Year** | **Age** | **Gender** | **Adopted Models** | **Pollutants** | **Exposure Assessment Method** | **Resolution** | **Disease Coding** | **Cite** |
| --- | --- | --- | --- | --- | --- | --- | --- | --- | --- | --- | --- | --- | --- | --- |
| Kan et al. | 2007 | Time-serise | Shanghai |  | 2004 | 2005 | All | All | GAMs | PM_2.5_, PM_2.5-10_, PM_10_ | Station average | city level | ICD-10 (A00-R99) | (Kan et al., 2007) |
| Ma et al. | 2011 | Case-crossover | Shenyang |  | 2006 | 2008 | All | All | The time-stratified case-crossover approach | PM_2.5_ | Station average | city level | ICD-10 (A00-R99) | (Ma et al., 2011) |
| Cao et al. | 2012 | Time-serise | Xi'an |  | 2004 | 2008 | All | All | GLMs | PM_2.5_ and components | Station average | city level | ICD-10 (A00-R99) | (Cao et al., 2012) |
| Li et al. | 2015 | Time-serise | Beijing | Urban | 2005 | 2009 | All | All | Poisson time series models | PM_2.5_ | single station | city level | ICD-10 | (Li et al., 2015a) |
| Zhang et al. | 2016 | Time-serise | Shenzhen |  | 2013 | 2013 | All | All | GAMs | PM_2.5_, PM_10_ | IDW interpolation average | city level | ICD-10 | (Zhang et al., 2016) |
| Chen et al. | 2017 | Time-serise | China | 272 cities | 2013 | 2015 | All | All | The time-stratified case-crossover approach | PM_2.5_ | Station average | city level | ICD-10 (A00-R99) | (Chen et al., 2017b) |
| Zhang et al. | 2017 | Time-serise | Jinan |  | 2011 | 2015 | All | All | GLMs | PM_2.5_, PM_10_, NO_2_, SO_2_ | Station average | city level | ICD-10 (A00-R99) | (Zhang et al., 2017b) |
| Li et al. | 2018 | Case-crossover | Beijing | Urban | 2009 | 2012 | All | All | The time-stratified case-crossover approach | PM_2.5_ | Station average | city level | ICD-10 (A00-R99) | (Li et al., 2018b) |
| Wu et al. | 2018 | Time-serise | Guangzhou |  | 2006 | 2016 | All | All | Poisson regression | PM_2.5_, PM_2.5-10_, PM_10_ | Station average | city level | ICD-10 (A00-R99) | (Wu et al., 2018) |
| Qiu et al. | 2018 | Time-serise | Hong Kong |  | 2011 | 2015 | >65 | All | GAMs | PM_2.5_, PM_coarse_ | Station average | city level | ICD-10 (A00-R99) | (Qiu et al., 2018) |
| Yu et al. | 2019 | Time-serise | Changzhou |  | 2015 | 2016 | All | All | GAMs | PM_2.5_, PM_10_ | Station IDW average | Sub-urban level | ICD-10 (A00-R99) | (Yu et al., 2019) |
| Xu et al. | 2020 | Case-crossover | Beijing |  | 2012 | 2013 | All | All | The time-stratified case-crossover approach | PM_2.5_ | Kriging average | county level | ICD-10 (A00-R99) | (Xu et al., 2020b) |
| Dong et al. | 2020 | Time-serise | China | 36 cities | 2013 | 2017 | All | All | GAMs | PM_2.5_ | I/O exposure level | city level | ICD-10 (A00-R99) | (Dong et al., 2020) |
| Chen et al. | 2021 | Time-serise | Lishui |  | 2015 | 2019 | All | All | GAMs | PM_2.5_, O_3_ | Station average | city level | ICD-10 (A00-R99) | (Chen et al., 2021d) |
| Sun et al. | 2022 | Time-serise | China | 250 counties | 2013 | 2018 | All | All | GAMs | PM_2.5_ | Station average | county level | ICD-10 (A00-R99) | (Sun et al., 2022) |
| Zhou et al. | 2022 | Case-crossover | China | 32 counties | 2011 | 2013 | All | All | The time-stratified case-crossover approach | PM_2.5_ | CMAQ | county level | ICD-10 (A00-R99) | (Zhou et al., 2022) |
| Chen et al. | 2022 | Time-serise | Jiangsu |  | 2015 | 2019 | All | All | GAMs | PM_2.5_, PM_10_ | Station average | city level | ICD-10 (A00-R99) | (Chen et al., 2022c) |
| Guan et al. | 2022 | Time-serise | Shijiazhuang |  | 2015 | 2020 | All | All | GAMs | PM_2.5_ | NA | city level | ICD-10 (A00-R99) | (Guan et al., 2022) |
| Lu et al. | 2023 | Time-serise | Baotou |  | 2015 | 2019 | All | All | GAMs | PM_2.5_, PM_10_, NO_2_, SO_2_, O_3_, CO | Station average | city level | ICD-10 (A00-R99) | (Lu et al., 2023) |
| Liu et al. | 2023 | Case-crossover | China | 6 provinces | 2013 | 2018 | All | All | The time-stratified case-crossover approach | PM_2.5_ | Machine learning | street level | ICD-10 (A00-R99) | (Liu et al., 2023a) |

1. Short-term exposure effect of PM_2.5_ to cardiovascular mortality

| **Authors** | **Year** | **Study design** | **Study Area** | **Area Note** | **Start Year** | **End Year** | **Age** | **Gender** | **Adopted Models** | **Pollutants** | **Exposure Assessment Method** | **Resolution** | **Disease Coding** | **Cite** |
| --- | --- | --- | --- | --- | --- | --- | --- | --- | --- | --- | --- | --- | --- | --- |
| Ma et al. | 2011 | Case-crossover | Shenyang |  | 2006 | 2008 | All | All | The time-stratified case-crossover approach | PM_2.5_ | Station average | city level | ICD-10 (I00-I99) | (Ma et al., 2011) |
| Cao et al. | 2012 | Time-serise | Xi'an |  | 2004 | 2008 | All | All | GLMs | PM_2.5_ and components | Station average | city level | ICD-10 (I00-I99) | (Cao et al., 2012) |
| Dai et al. | 2015 | Time-serise | Shanghai |  | 2006 | 2011 | All | All | GAMs | PM_2.5_, PM_10_, SO_2_, NO_2_, O_3_, CO | Station average | city level | ICD-10 (I20-I25) | (Dai et al., 2015) |
| Chen et al. | 2017 | Time-serise | China | 272 cities | 2013 | 2015 | All | All | The time-stratified case-crossover approach | PM_2.5_ | Station average | city level | ICD-10 (I00-I99) | (Chen et al., 2017b) |
| Zhang et al. | 2017 | Time-serise | Jinan |  | 2011 | 2015 | All | All | GLMs | PM_2.5_, PM_10_, NO_2_, SO_2_ | Station average | city level | ICD-10 (I00-I99) | (Zhang et al., 2017b) |
| Wu et al. | 2018 | Time-serise | Guangzhou |  | 2006 | 2016 | All | All | Poisson regression | PM_2.5_, PM_2.5-10_, PM_10_ | Station average | city level | ICD-10 (I00-I99) | (Wu et al., 2018) |
| Qiu et al. | 2018 | Time-serise | Hong Kong |  | 2011 | 2015 | >65 | All | GAMs | PM_2.5_, PM_coarse_ | Station average | city level | ICD-10 (I00-I99) | (Qiu et al., 2018) |
| Gong et al. | 2019 | Time-serise | Beijing |  | 2005 | 2011 | All | All | GAMs | PM_2.5_ | single station | city level | ICD-10 | (Gong et al., 2019a) |
| Yu et al. | 2019 | Time-serise | Changzhou |  | 2015 | 2016 | All | All | GAMs | PM_2.5_, PM_10_ | Station IDW average | Sub-urban level | ICD-10 (I00-I99) | (Yu et al., 2019) |
| Zhang et al. | 2019 | Time-serise | Yangzhou |  | 2015 | 2017 | All | All | GAMs | PM_2.5_ | Station average | city level | ICD-10 (I00-I99) | (Zhang, 2019b) |
| Xu et al. | 2020 | Case-crossover | Beijing |  | 2012 | 2013 | All | All | The time-stratified case-crossover approach | PM_2.5_ | Kriging average | county level | ICD-10 (I00-I99) | (Xu et al., 2020b) |
| Dong et al. | 2020 | Time-serise | China | 36 cities | 2013 | 2017 | All | All | GAMs | PM_2.5_ | I/O exposure level | city level | ICD-10 (I00-I99) | (Dong et al., 2020) |
| Ji et al. | 2020 | Time-serise | Fuzhou |  | 2016 | 2019 | All | All | GAMs | PM_2.5_ | Station average | city level | ICD-10 (I00-I99) | (Ji et al., 2020b) |
| Xu et al. | 2020 | Time-serise | Hefei |  | 2007 | 2016 | All | All | DLNM | PM_2.5_, PM_10_, NO_2_, SO_2_, O_3_, CO | Station average | city level | ICD-10 (I00-I99) | (Xu et al., 2020a) |
| Tian et al. | 2020 | Time-serise | Shanghai |  | 2012 | 2014 | All | All | GAMs | PM_2.5_ | single station | city level | ICD-10 (I00-I78) | (Tian et al., 2020b) |
| Liu et al. | 2021 | Case-crossover | Hubei |  | 2013 | 2018 | All | All | The time-stratified case-crossover approach | PM_2.5_, PM_10_, NO_2_, SO_2_, O_3_, CO | Station IDW average | Sub-city level | ICD-10 (I21) | (Liu et al., 2021b) |
| Chen et al. | 2021 | Time-serise | Nanjing |  | 2004 | 2019 | All | All | GAMs | PM_2.5_, PM_10_, NO_2_, SO_2_ | Station average | city level | ICD-10 (I00-I99) | (Chen et al., 2021b) |
| Sun et al. | 2022 | Time-serise | China | 250 counties | 2013 | 2018 | All | All | GAMs | PM_2.5_ | Station average | county level | ICD-10 (I00-I99) | (Sun et al., 2022) |
| Zhou et al. | 2022 | Case-crossover | China | 32 counties | 2011 | 2013 | All | All | The time-stratified case-crossover approach | PM_2.5_ | CMAQ | county level | ICD-10 (I00-I99) | (Zhou et al., 2022) |
| Xu et al. | 2022 | Case-crossover | Jiangsu |  | 2015 | 2019 | All | All | The time-stratified case-crossover approach | PM_2.5_, PM_10_, SO_2_, NO_2_, O_3_, CO | CHAP | 10 km | ICD-10 (I60-I64) | (Xu et al., 2022) |
| Chen et al. | 2022 | Time-serise | Jiangsu |  | 2015 | 2019 | All | All | GAMs | PM_2.5_, PM_10_ | Station average | city level | ICD-10 (I00-I99) | (Chen et al., 2022c) |
| Chen et al. | 2022 | Case-crossover | Jiangsu |  | 2015 | 2020 | All | All | The time-stratified case-crossover approach | PM_2.5_, PM_10_, SO_2_, NO_2_, O_3_ | CHAP | 10 km | ICD-10 (I25) | (Chen et al., 2022b) |
| Guan et al. | 2022 | Time-serise | Shijiazhuang |  | 2015 | 2020 | All | All | GAMs | PM_2.5_ | NA | city level | ICD-10 (I00-I99) | (Guan et al., 2022) |
| Mo et al. | 2023 | Case-crossover | China | 32 counties | 2011 | 2013 | All | All | Space-time-stratified case-crossover | PM_2.5_ and components | CTMs | county level | ICD-10 (I21) | (Mo et al., 2023) |
| Liu et al. | 2023 | Case-crossover | China | 6 provinces | 2013 | 2018 | All | All | The time-stratified case-crossover approach | PM_2.5_ | Machine learning | street level | ICD-10 (I00-I99) | (Liu et al., 2023a) |
| Ma et al. | 2023 | Case-crossover | Qingdao |  | 2015 | 2019 | All | All | The time-stratified case-crossover approach | PM_2.5_ and components | TAP | city level | ICD-10 | (Ma et al., 2023) |
| Peng et al. | 2023 | Time-serise | Unknown |  | 2015 | 2021 | All | All | GAMs | PM_2.5_, O_3_ | Station average | city level | ICD-10 (I00-I99) | (Peng, 2023) |
| Pu et al. | 2023 | Time-serise | Wuhan |  | 2013 | 2019 | All | All | GAMs | PM_2.5_, PM_10_, SO_2_, NO_2_, O_3_ | Station average | city level | ICD-10 (I10-I15) | (Pu et al., 2023) |
| Zhang et al. | 2023 | Time-serise | Yangquan |  | 2019 | 2020 | All | All | GAMs | PM_2.5_ | NA | city level | ICD-10 (I00-I99) | (Zhang, 2023) |

1. Short-term exposure effect of PM_2.5_ to respiratory mortality

| **Authors** | **Year** | **Study design** | **Study Area** | **Area Note** | **Start Year** | **End Year** | **Age** | **Gender** | **Adopted Models** | **Pollutants** | **Exposure Assessment Method** | **Resolution** | **Disease Coding** | **Cite** |
| --- | --- | --- | --- | --- | --- | --- | --- | --- | --- | --- | --- | --- | --- | --- |
| Kan et al. | 2007 | Time-serise | Shanghai |  | 2004 | 2005 | All | All | GAMs | PM_2.5_, PM_2.5-10_, PM_10_ | Station average | city level | ICD-10 (J00-J98) | (Kan et al., 2007) |
| Ma et al. | 2011 | Case-crossover | Shenyang |  | 2006 | 2008 | All | All | The time-stratified case-crossover approach | PM_2.5_ | Station average | city level | ICD-10 (J00-J98) | (Ma et al., 2011) |
| Cao et al. | 2012 | Time-serise | Xi'an |  | 2004 | 2008 | All | All | GLMs | PM_2.5_ and components | Station average | city level | ICD-10 (J00-J98) | (Cao et al., 2012) |
| Li et al. | 2013 | Time-serise | Beijing | Urban | 2004 | 2009 | All | All | GAMs | PM_2.5_ | single station | city level | ICD-10 | (Li et al., 2013a) |
| Lin et al. | 2016 | Time-serise | Hong Kong |  | 1998 | 2011 | All | All | GAMs | PM_2.5_ | Station average | city level | ICD-10 (J00-J99) | (Lin et al., 2016b) |
| Chen et al. | 2017 | Time-serise | China | 272 cities | 2013 | 2015 | All | All | The time-stratified case-crossover approach | PM_2.5_ | Station average | city level | ICD-10 (J00-J98) | (Chen et al., 2017b) |
| Li et al. | 2018 | Case-crossover | Beijing | Urban | 2009 | 2012 | All | All | The time-stratified case-crossover approach | PM_2.5_ | Station average | city level | ICD-10 (J00-J99) | (Li et al., 2018b) |
| Wu et al. | 2018 | Time-serise | Guangzhou |  | 2006 | 2016 | All | All | Poisson regression | PM_2.5_, PM_2.5-10_, PM_10_ | Station average | city level | ICD-10 (J00-J99) | (Wu et al., 2018) |
| Qiu et al. | 2018 | Time-serise | Hong Kong |  | 2011 | 2015 | >65 | All | GAMs | PM_2.5_, PM_coarse_ | Station average | city level | ICD-10 (J00-J99) | (Qiu et al., 2018) |
| Yu et al. | 2019 | Time-serise | Changzhou |  | 2015 | 2016 | All | All | GAMs | PM_2.5_, PM_10_ | Station IDW average | Sub-urban level | ICD-10 (J00-J99) | (Yu et al., 2019) |
| Liu et al. | 2019 | Case-crossover | Hubei |  | 2013 | 2018 | All | All | Conditional Logistic regression | PM_2.5_, PM_10_, SO_2_, NO_2_, O_3_, CO | Station IDW average | Sub-city level | ICD-10 (J45-J46) | (Liu et al., 2019d) |
| Xu et al. | 2020 | Case-crossover | Beijing |  | 2012 | 2013 | All | All | The time-stratified case-crossover approach | PM_2.5_ | Kriging average | county level | ICD-10 (J00-J99) | (Xu et al., 2020b) |
| Dong et al. | 2020 | Time-serise | China | 36 cities | 2013 | 2017 | All | All | GAMs | PM_2.5_ | I/O exposure level | city level | ICD-10 (J00-J98) | (Dong et al., 2020) |
| Yan et al. | 2021 | Time-serise | Wuhan |  | 2014 | 2019 | All | All | GAMs | PM_2.5_, PM_10_, SO_2_, NO_2_, O_3_ | Station average | city level | ICD-10 (J40-J44,I27.9) | (Yan et al., 2021) |
| Sun et al. | 2022 | Time-serise | China | 250 counties | 2013 | 2018 | All | All | GAMs | PM_2.5_ | Station average | county level | ICD-10 (J00-J99) | (Sun et al., 2022) |
| Zhou et al. | 2022 | Case-crossover | China | 32 counties | 2011 | 2013 | All | All | The time-stratified case-crossover approach | PM_2.5_ | CMAQ | county level | ICD-10 (J00-J99) | (Zhou et al., 2022) |
| Liu et al. | 2022 | Case-crossover | China |  | 2015 | 2020 | All | All | The time-stratified case-crossover approach | PM_10_, PM_2.5_, PM_1_ | CHAP | 10 km | ICD-10 (J45-J46) | (Liu et al., 2022c) |
| Song et al. | 2022 | Time-serise | Jiangsu |  | 2016 | 2017 | All | All | Conditional Logistic regression | PM_2.5_, PM_1_ | CHAP | 1 km | ICD-10 (J45-J46) | (Song et al., 2022) |
| Guan et al. | 2022 | Time-serise | Shijiazhuang |  | 2015 | 2020 | All | All | GAMs | PM_2.5_ | NA | city level | ICD-10 (J00-J99) | (Guan et al., 2022) |
| Liu et al. | 2023 | Case-crossover | China | 6 provinces | 2013 | 2018 | All | All | The time-stratified case-crossover approach | PM_2.5_ | Machine learning | street level | ICD-10 (J00-J99) | (Liu et al., 2023a) |
| Zhai et al. | 2023 | Time-serise | Sichuan |  | 2018 | 2021 | All | All | Bayesian kernel machine regression | PM_2.5_ and components | TAP | city level | ICD-10 (J00-J99) | (Zhai et al., 2023) |
| Peng et al. | 2023 | Time-serise | Unknown |  | 2015 | 2021 | All | All | GAMs | PM_2.5_, O_3_ | Station average | city level | ICD-10 (J00-J99) | (Peng, 2023) |

1. Short-term exposure effect of NO_2_ to all-cause mortality

| **Authors** | **Year** | **Study design** | **Study Area** | **Area Note** | **Start Year** | **End Year** | **Age** | **Gender** | **Adopted Models** | **Pollutants** | **Exposure Assessment Method** | **Resolution** | **Disease Coding** | **Cite** |
| --- | --- | --- | --- | --- | --- | --- | --- | --- | --- | --- | --- | --- | --- | --- |
| Wong et al. | 2001 | Time-serise | Hong Kong |  | 1995 | 1997 | All | All | Poisson regression | PM_10_, NO_2_, SO_2_ | Station average | city level | ICD-9 (<800) | (Wong et al., 2001) |
| Chen et al. | 2012 | Time-serise | China | 17 cities | 1996 | 2008 | All | All | Poisson regression | NO_2_ | Station average | city level | ICD-10 (A00-R99) | (Chen et al., 2012) |
| Yang et al. | 2013 | Time-serise | Beijing |  | 2009 | 2010 | All | All | GAMs | PM_10_, NO_2_, CO | Station average | city level | ICD-10 (A00-R99) | (Yang et al., 2013) |
| Lu et al. | 2015 | Time-serise | Nanjing |  | 2009 | 2013 | All | All | Poisson regression | PM_10_, NO_2_, SO_2_ | Station average | city level | ICD-10 (A00-R99) | (Lu et al., 2015) |
| Qu et al. | 2018 | Time-serise | Changchun |  | 2014 | 2017 | >65 | All | GAMs | PM_2.5_, PM_10_, SO_2_, NO_2_, O_3_ | Station average | city level | ICD-10 (A00-R99) | (Qu et al., 2018) |
| Chen et al. | 2018 | Time-serise | China | 272 cities | 2013 | 2015 | All | All | Two-stage Bayesian hierarchical model | NO_2_ | Station average | city level | ICD-10 (A00-R99) | (Chen et al., 2018b) |
| Zhong et al. | 2018 | Time-serise | Wuhan | Jiangan | 2002 | 2010 | All | All | GAMs | PM_10_, NO_2_, SO_2_ | Station average | county level | ICD-10 (A00-R99) | (Zhong et al., 2018) |
| Wu et al. | 2019 | Time-serise | Guangzhou |  | 2006 | 2016 | All | All | GAMs | NO_2_, O_3_ | Station average | city level | ICD-10 (A00-R99) | (Wu et al., 2019a) |
| Liu et al. | 2020 | Time-serise | Lanzhou |  | 2004 | 2017 | All | All | GAMs | PM_10_, NO_2_, SO_2_ | Station average | city level | ICD-10 (A00-R99) | (Liu et al., 2020) |
| Hu et al. | 2021 | Time-serise | China | 271 cities | 2013 | 2015 | All | All | GAMs | NO_2_ | I/O exposure level | city level | NA | (Hu et al., 2021) |
| Liu et al. | 2021 | Time-serise | Harbin |  | 2014 | 2018 | All | All | GAMs | PM_10_, SO_2_, NO_2_ | Station average | city level | ICD-10 (A00-R99) | (Liu et al., 2021a) |
| Deng et al. | 2021 | Time-serise | Hefei |  | 2014 | 2017 | All | All | GAMs | SO_2_, NO_2_ | Station average | city level | ICD-10 (A00-R99) | (Deng et al., 2021) |

1. Short-term exposure effect of NO_2_ to cardiovascular mortality

| **Authors** | **Year** | **Study design** | **Study Area** | **Area Note** | **Start Year** | **End Year** | **Age** | **Gender** | **Adopted Models** | **Pollutants** | **Exposure Assessment Method** | **Resolution** | **Disease Coding** | **Cite** |
| --- | --- | --- | --- | --- | --- | --- | --- | --- | --- | --- | --- | --- | --- | --- |
| Wong et al. | 2002 | Ecological | Hong Kong |  | 1995 | 1998 | All | All | Poisson regression | PM_10_, NO_2_, SO_2_, O_3_ | Station average | city level | ICD-9 (410-414) | (Wong et al., 2002) |
| Chen et al. | 2012 | Time-serise | China | 17 cities | 1996 | 2008 | All | All | Poisson regression | NO_2_ | Station average | city level | ICD-10 (I00-I99) | (Chen et al., 2012) |
| Dai et al. | 2015 | Time-serise | Shanghai |  | 2006 | 2011 | All | All | GAMs | PM_2.5_, PM_10_, SO_2_, NO_2_, O_3_, CO | Station average | city level | ICD-10 (I20-I25) | (Dai et al., 2015) |
| Qu et al. | 2018 | Time-serise | Changchun |  | 2014 | 2017 | >65 | All | GAMs | PM_2.5_, PM_10_, SO_2_, NO_2_, O_3_ | Station average | city level | ICD-10 (I00-I99) | (Qu et al., 2018) |
| Chen et al. | 2018 | Time-serise | China | 272 cities | 2013 | 2015 | All | All | Two-stage Bayesian hierarchical model | NO_2_ | Station average | city level | ICD-10 (I00-I99) | (Chen et al., 2018b) |
| Zhong et al. | 2018 | Time-serise | Jiangan |  | 2002 | 2010 | All | All | GAMs | PM_10_, NO_2_, SO_2_ | Station average | county level | ICD-10 (I00-I99) | (Zhong et al., 2018) |
| Li et al. | 2019 | Time-serise | Beijing |  | 2009 | 2010 | All | All | GLMs | PM_10_, NO_2_ | Station average | district level | ICD-10 (I00-I99) | (Li et al., 2019a) |
| Chen et al. | 2019 | Case-crossover | Chengdu |  | 2013 | 2017 | >60 | All | The time-stratified case-crossover approach | PM_2.5_, NO_2_, SO_2_, O_3_, CO | Station average | city level | ICD-10 (I27) | (Chen et al., 2019c) |
| Wu et al. | 2019 | Time-serise | Guangzhou |  | 2006 | 2016 | All | All | GAMs | NO_2_, O_3_ | Station average | city level | ICD-10 (I00-I99) | (Wu et al., 2019a) |
| Duan et al. | 2019 | Time-serise | Shenzhen |  | 2013 | 2017 | All | All | GAMs | NO_2_ | Station average | city level | ICD-10 (I00-I99) | (Duan et al., 2019) |
| Xu et al. | 2020 | Time-serise | Hefei |  | 2007 | 2016 | All | All | DLNM | PM_2.5_, PM_10_, NO_2_, SO_2_, O_3_, CO | Station average | city level | ICD-10 (I00-I99) | (Xu et al., 2020a) |
| Liu et al. | 2021 | Case-crossover | Hubei |  | 2013 | 2018 | All | All | The time-stratified case-crossover approach | PM_2.5_, PM_10_, NO_2_, SO_2_, O_3_, CO | Station IDW average | Sub-city level | ICD-10 (I21) | (Liu et al., 2021b) |
| Chen et al. | 2021 | Time-serise | Nanjing |  | 2004 | 2019 | All | All | GAMs | PM_2.5_, PM_10_, NO_2_, SO_2_ | Station average | city level | ICD-10 (I00-I99) | (Chen et al., 2021b) |
| Chen et al. | 2022 | Time-serise | Enshi |  | 2015 | 2018 | All | All | GAMs | NO_2_ | Station average | city level | ICD-10 (I00-I99) | (Chen et al., 2022d) |
| Xu et al. | 2022 | Case-crossover | Jiangsu |  | 2015 | 2019 | All | All | The time-stratified case-crossover approach | PM_2.5_, PM_10_, SO_2_, NO_2_, O_3_, CO | CHAP | 10 km | ICD-10 (I60-I64) | (Xu et al., 2022) |
| Chen et al. | 2022 | Case-crossover | Jiangsu |  | 2015 | 2020 | All | All | The time-stratified case-crossover approach | PM_2.5_, PM_10_, SO_2_, NO_2_, O_3_ | CHAP | 10 km | ICD-10 (I25) | (Chen et al., 2022b) |
| Pu et al. | 2023 | Time-serise | Wuhan |  | 2013 | 2019 | All | All | GAMs | PM_2.5_, PM_10_, SO_2_, NO_2_, O_3_ | Station average | city level | ICD-10 (I10-I15) | (Pu et al., 2023) |

1. Short-term exposure effect of NO_2_ to respiratory mortality

| **Authors** | **Year** | **Study design** | **Study Area** | **Area Note** | **Start Year** | **End Year** | **Age** | **Gender** | **Adopted Models** | **Pollutants** | **Exposure Assessment Method** | **Resolution** | **Disease Coding** | **Cite** |
| --- | --- | --- | --- | --- | --- | --- | --- | --- | --- | --- | --- | --- | --- | --- |
| Wong et al. | 2002 | Ecological | Hong Kong |  | 1995 | 1998 | All | All | Poisson regression | PM_10_, NO_2_, SO_2_, O_3_ | Station average | city level | ICD-9 (461-519) | (Wong et al., 2002) |
| Chen et al. | 2012 | Time-serise | China | 17 cities | 1996 | 2008 | All | All | Poisson regression | NO_2_ | Station average | city level | ICD-10 (J00-J98) | (Chen et al., 2012) |
| Yang et al. | 2013 | Time-serise | Beijing |  | 2009 | 2010 | All | All | GAMs | PM_10_, NO_2_, CO | Station average | city level | ICD-10 (J00-J98) | (Yang et al., 2013) |
| Qian et al. | 2015 | Case-crossover | Shanghai |  | 2003 | 2012 | All | All | The time-stratified case-crossover approach | PM_10_, NO_2_, SO_2_ | NA | city level | ICD-10 (J40-J47) | (Qian et al., 2015) |
| Chen et al. | 2018 | Time-serise | China | 272 cities | 2013 | 2015 | All | All | Two-stage Bayesian hierarchical model | NO_2_ | Station average | city level | ICD-10 (J00-J98) | (Chen et al., 2018b) |
| Wu et al. | 2019 | Time-serise | Guangzhou |  | 2006 | 2016 | All | All | GAMs | NO_2_, O_3_ | Station average | city level | ICD-10 (J00-J98) | (Wu et al., 2019a) |
| Liu et al. | 2019 | Case-crossover | Hubei |  | 2013 | 2018 | All | All | Conditional Logistic regression | PM_2.5_, PM_10_, SO_2_, NO_2_, O_3_, CO | Station IDW average | Sub-city level | ICD-10 (J45-J46) | (Liu et al., 2019d) |
| Zhang et al. | 2020 | Case-crossover | Wuhan |  | 2003 | 2013 | All | All | The time-stratified case-crossover approach | PM_10_, NO_2_, SO_2_ | Station average | city level | ICD-10 (J45-J46) | (Zhang et al., 2020b) |
| Shao et al. | 2021 | Time-serise | Hefei |  | 2014 | 2018 | All | All | DLNM | PM_10_, NO_2_, SO_2_, O_3_, CO | Station average | city level | ICD-10 (J00-J99) | (Shao et al., 2021) |
| Yan et al. | 2021 | Time-serise | Wuhan |  | 2014 | 2019 | All | All | GAMs | PM_2.5_, PM_10_, SO_2_, NO_2_, O_3_ | Station average | city level | ICD-10 (J40-J44,I27.9) | (Yan et al., 2021) |

1. Short-term exposure effect of O_3_ to all-cause mortality

| **Authors** | **Year** | **Study design** | **Study Area** | **Area Note** | **Start Year** | **End Year** | **Age** | **Gender** | **Adopted Models** | **Pollutants** | **Exposure Assessment Method** | **Resolution** | **Disease Coding** | **Cite** |
| --- | --- | --- | --- | --- | --- | --- | --- | --- | --- | --- | --- | --- | --- | --- |
| Kan et al. | 2010 | Time-serise | Shanghai |  | 2001 | 2004 | All | All | GAMs | PM_10_, NO_2_, SO_2_, O_3_ | Station average | city level | ICD-10 (A00-R99) | (Kan et al., 2010) |
| Qian et al. | 2010 | Time-serise | Wuhan |  | 2000 | 2004 | All | All | GAMs | PM_10_, NO_2_, SO_2_, O_3_ | Station average | city level | ICD-10 (A00-R99) | (Qian et al., 2010) |
| Tao et al. | 2012 | Time-serise | PRD | 4 cities | 2006 | 2008 | All | All | GLMs | NO_2_, O_3_ | Station average | city level | ICD-10 (A00-R99) | (Tao et al., 2012) |
| Yang et al. | 2012 | Time-serise | Suzhou |  | 2006 | 2008 | All | All | GAMs | O_3_ | Station average | city level | ICD-10 (A00-R99) | (Yang et al., 2012b) |
| Yin et al. | 2017 | Time-serise | China | 272 cities | 2013 | 2015 | All | All | Two-stage Bayesian hierarchical model | O_3_ | Station average | city level | ICD-10 (A00-R99) | (Yin et al., 2017b) |
| Li et al. | 2018 | Case-crossover | Beijing | Miyun | 2005 | 2013 | All | All | The time-stratified case-crossover approach | O_3_ | single station | city level | ICD-10 (A00-R99) | (Liu et al., 2018) |
| Wu et al. | 2019 | Time-serise | Guangzhou |  | 2006 | 2016 | All | All | GAMs | NO_2_, O_3_ | Station average | city level | ICD-10 (A00-R99) | (Wu et al., 2019a) |
| Lin et al. | 2022 | Time-serise | Jiangsu |  | 2015 | 2018 | All | All | GAMs | O_3_ | Machine learning | county level | ICD-10 (A00-R99) | (Lin et al., 2022) |
| Chen et al. | 2022 | Time-serise | Kunminng |  | 2017 | 2019 | All | All | GAMs | O_3_ | Station average | city level | ICD-10 (A00-R99) | (Chen et al., 2022a) |
| He et al. | 2022 | Time-serise | Nantong |  | 2017 | 2020 | All | All | GLM | O_3_ | Station average | city level | ICD-10 (A00-R99) | (He, 2022) |
| Chen et al. | 2023 | Time-serise | China | 323 counties | 2013 | 2018 | All | All | unconstrained distributed log linear model | O_3_ | Station average | city level | ICD-10 (A00-R99) | (Chen et al., 2023a) |

1. Short-term exposure effect of O_3_ to cardiovascular mortality

| **Authors** | **Year** | **Study design** | **Study Area** | **Area Note** | **Start Year** | **End Year** | **Age** | **Gender** | **Adopted Models** | **Pollutants** | **Exposure Assessment Method** | **Resolution** | **Disease Coding** | **Cite** |
| --- | --- | --- | --- | --- | --- | --- | --- | --- | --- | --- | --- | --- | --- | --- |
| Wong et al. | 2002 | Ecological | Hong Kong |  | 1995 | 1998 | All | All | Poisson regression | PM_10_, NO_2_, SO_2_, O_3_ | Station average | city level | ICD-9 (410-414) | (Wong et al., 2002) |
| Kan et al. | 2010 | Time-serise | Shanghai |  | 2001 | 2004 | All | All | GAMs | PM_10_, NO_2_, SO_2_, O_3_ | Station average | city level | ICD-10 (I00-I99) | (Kan et al., 2010) |
| Qian et al. | 2010 | Time-serise | Wuhan |  | 2000 | 2004 | All | All | GAMs | PM_10_, NO_2_, SO_2_, O_3_ | Station average | city level | ICD-10 (I00-I99) | (Qian et al., 2010) |
| Tao et al. | 2012 | Time-serise | PRD | 4 cities | 2006 | 2008 | All | All | GLMs | NO_2_, O_3_ | Station average | city level | ICD-10 (I00-I99) | (Tao et al., 2012) |
| Yang et al. | 2012 | Time-serise | Suzhou |  | 2006 | 2008 | All | All | GAMs | O_3_ | Station average | city level | ICD-10 (I00-I99) | (Yang et al., 2012b) |
| Yin et al. | 2017 | Time-serise | China | 272 cities | 2013 | 2015 | All | All | Two-stage Bayesian hierarchical model | O_3_ | Station average | city level | ICD-10 (I00-I99) | (Yin et al., 2017b) |
| Li et al. | 2018 | Case-crossover | Beijing | Miyun | 2005 | 2013 | All | All | The time-stratified case-crossover approach | O_3_ | single station | city level | ICD-10 (I00-I99) | (Liu et al., 2018) |
| Wu et al. | 2019 | Time-serise | Guangzhou |  | 2006 | 2016 | All | All | GAMs | NO_2_, O_3_ | Station average | city level | ICD-10 (I00-I99) | (Wu et al., 2019a) |
| Xu et al. | 2020 | Time-serise | Hefei |  | 2007 | 2016 | All | All | DLNM | PM_2.5_, PM_10_, NO_2_, SO_2_, O_3_, CO | Station average | city level | ICD-10 (I00-I99) | (Xu et al., 2020a) |
| Xu et al. | 2022 | Case-crossover | Jiangsu |  | 2015 | 2019 | All | All | The time-stratified case-crossover approach | PM_2.5_, PM_10_, SO_2_, NO_2_, O_3_, CO | CHAP | 10 km | ICD-10 (I60-I64) | (Xu et al., 2022) |
| Lin et al. | 2022 | Time-serise | Jiangsu |  | 2015 | 2018 | All | All | GAMs | O_3_ | Machine learning | county level | ICD-10 (I00-I99) | (Lin et al., 2022) |
| Chen et al. | 2022 | Case-crossover | Jiangsu |  | 2015 | 2020 | All | All | The time-stratified case-crossover approach | PM_2.5_, PM_10_, SO_2_, NO_2_, O_3_ | CHAP | 10 km | ICD-10 (I25) | (Chen et al., 2022a) |
| Wu et al. | 2022 | Time-serise | Nanchang |  | 2014 | 2020 | All | All | GAMs | O_3_ | Station average | city level | ICD-10 (I05-I52) | (Wu et al., 2022) |
| He et al. | 2022 | Time-serise | Nantong |  | 2017 | 2020 | All | All | GLM | O_3_ | Station average | city level | ICD-10 (I00-I99) | (He, 2022) |
| Chen et al. | 2023 | Time-serise | China | 323 counties | 2013 | 2018 | All | All | unconstrained distributed log linear model | O_3_ | Station average | city level | ICD-10 (I00-I99) | (Chen et al., 2023a) |
| Gao et al. | 2023 | Time-serise | Shenzhen |  | 2013 | 2019 | All | All | GAMs | O_3_ | Station average | city level | ICD-10 (I00-I99) | (Gao et al., 2023) |
| Peng et al. | 2023 | Time-serise | Unknown |  | 2015 | 2021 | All | All | GAMs | PM_2.5_, O_3_ | Station average | city level | ICD-10 (I00-I99) | (Peng, 2023) |
| Pu et al. | 2023 | Time-serise | Wuhan |  | 2013 | 2019 | All | All | GAMs | PM_2.5_, PM_10_, SO_2_, NO_2_, O_3_ | Station average | city level | ICD-10 (I10-I15) | (Pu et al., 2023) |

1. Short-term exposure effect of O_3_ to respiratory mortality

| **Authors** | **Year** | **Study design** | **Study Area** | **Area Note** | **Start Year** | **End Year** | **Age** | **Gender** | **Adopted Models** | **Pollutants** | **Exposure Assessment Method** | **Resolution** | **Disease Coding** | **Cite** |
| --- | --- | --- | --- | --- | --- | --- | --- | --- | --- | --- | --- | --- | --- | --- |
| Wong et al. | 2002 | Ecological | Hong Kong |  | 1995 | 1998 | All | All | Poisson regression | PM_10_, NO_2_, SO_2_, O_3_ | Station average | city level | ICD-9 (461-519) | (Wong et al., 2002) |
| Kan et al. | 2010 | Time-serise | Shanghai |  | 2001 | 2004 | All | All | GAMs | PM_10_, NO_2_, SO_2_, O_3_ | Station average | city level | ICD-10 (J00-J98) | (Kan et al., 2010) |
| Qian et al. | 2010 | Time-serise | Wuhan |  | 2000 | 2004 | All | All | GAMs | PM_10_, NO_2_, SO_2_, O_3_ | Station average | city level | ICD-10 (J00-J98) | (Qian et al., 2010) |
| Tao et al. | 2012 | Time-serise | PRD | 4 cities | 2006 | 2008 | All | All | GLMs | NO_2_, O_3_ | Station average | city level | ICD-10 (J00-J98) | (Tao et al., 2012) |
| Yin et al. | 2017 | Time-serise | China | 272 cities | 2013 | 2015 | All | All | Two-stage Bayesian hierarchical model | O_3_ | Station average | city level | ICD-10 (J00-J98) | (Yin et al., 2017b) |
| Wu et al. | 2019 | Time-serise | Guangzhou |  | 2006 | 2016 | All | All | GAMs | NO_2_, O_3_ | Station average | city level | ICD-10 (J00-J98) | (Wu et al., 2019a) |
| Liu et al. | 2019 | Case-crossover | Hubei |  | 2013 | 2018 | All | All | Conditional Logistic regression | PM_2.5_, PM_10_, SO_2_, NO_2_, O_3_, CO | Station IDW average | Sub-city level | ICD-10 (J45-J46) | (Liu et al., 2019d) |
| Chen et al. | 2021 | Time-serise | Lishui |  | 2015 | 2019 | All | All | GAMs | PM_2.5_, O_3_ | Station average | city level | ICD-10 (J00-J99) | (Chen et al., 2021d) |
| Lin et al. | 2022 | Time-serise | Jiangsu |  | 2015 | 2018 | All | All | GAMs | O_3_ | Machine learning | county level | ICD-10 (J00-J99) | (Lin et al., 2022) |
| Wu et al. | 2022 | Time-serise | Nanchang |  | 2014 | 2020 | All | All | GAMs | O_3_ | Station average | city level | ICD-10 (J00-J99) | (Wu et al., 2022) |
| He et al. | 2022 | Time-serise | Nantong |  | 2017 | 2020 | All | All | GLM | O_3_ | Station average | city level | ICD-10 (J00-J99) | (He, 2022) |
| Chen et al. | 2023 | Time-serise | China | 323 counties | 2013 | 2018 | All | All | unconstrained distributed log linear model | O_3_ | Station average | city level | ICD-10 (J00-J99) | (Chen et al., 2023a) |
| Peng et al. | 2023 | Time-serise | Unknown |  | 2015 | 2021 | All | All | GAMs | PM_2.5_, O_3_ | Station average | city level | ICD-10 (J00-J99) | (Peng, 2023) |

**Appendix 7: Summary of pollutant level distribution among the included literature**

As shown in the table, each result of all exposure-outcome pairs is determined by high or low levels of air pollutants, rather than within a narrow range. The average pollutant levels between studies fluctuate by at least one to several tens of micrograms per cubic meter, with some studies reporting levels exceeding 130 μg/m³, and others as low as 30 μg/m³. This effectively confirms that the range of pollutant levels is sufficiently broad to ensure the robustness of the final effect integration.

| Pollutant | Exposure period | Endpoint | Pollution level (μg/m^3^) | | |
| --- | --- | --- | --- | --- | --- |
|  |  |  | Median level | Min. level | Max. level |
| NO_2_ | Long-term | All-cause mortality | 45.89 | 19.10 | 104.0 |
|  |  | Cardiovascular mortality | 50.00 | 41.78 | 104.0 |
|  |  | Respiratory mortality | 50.00 | 41.78 | 104.0 |
|  | Short-term | All-cause mortality | 58.32 | 31.00 | 43.00 |
|  |  | Cardiovascular mortality | 40.25 | 21.40 | 62.00 |
|  |  | Respiratory mortality | 47.80 | 31.00 | 60.70 |
| O_3_ | Long-term | All-cause mortality | 108.0 | 100.0 | 116.0 |
|  |  | Cardiovascular mortality | 78.88 | 68.05 | 89.70 |
|  |  | Respiratory mortality | - | - | - |
|  | Short-term | All-cause mortality | 84.30 | 57.70 | 130.0 |
|  |  | Cardiovascular mortality | 84.21 | 33.93 | 130.0 |
|  |  | Respiratory mortality | 92.00 | 33.93 | 130.0 |
| PM_2.5_ | Long-term | All-cause mortality | 47.10 | 41.40 | 65.50 |
|  |  | Cardiovascular mortality | 45.65 | 37.34 | 59.40 |
|  |  | Respiratory mortality | 41.80 | 26.30 | 58.36 |
|  | Short-term | All-cause mortality | 56.80 | 29.10 | 182.2 |
|  |  | Cardiovascular mortality | 55.28 | 21.69 | 182.2 |
|  |  | Respiratory mortality | 56.40 | 29.10 | 182.2 |

**Appendix 8: Details of the meta-analysis of the long- and short-term exposure effect of PM_2.5_, NO_2_ and O_3_ on cause-specific mortality**

Short-term exposure effect of PM_2.5_, NO_2_ and O_3_ on cause-specific mortality


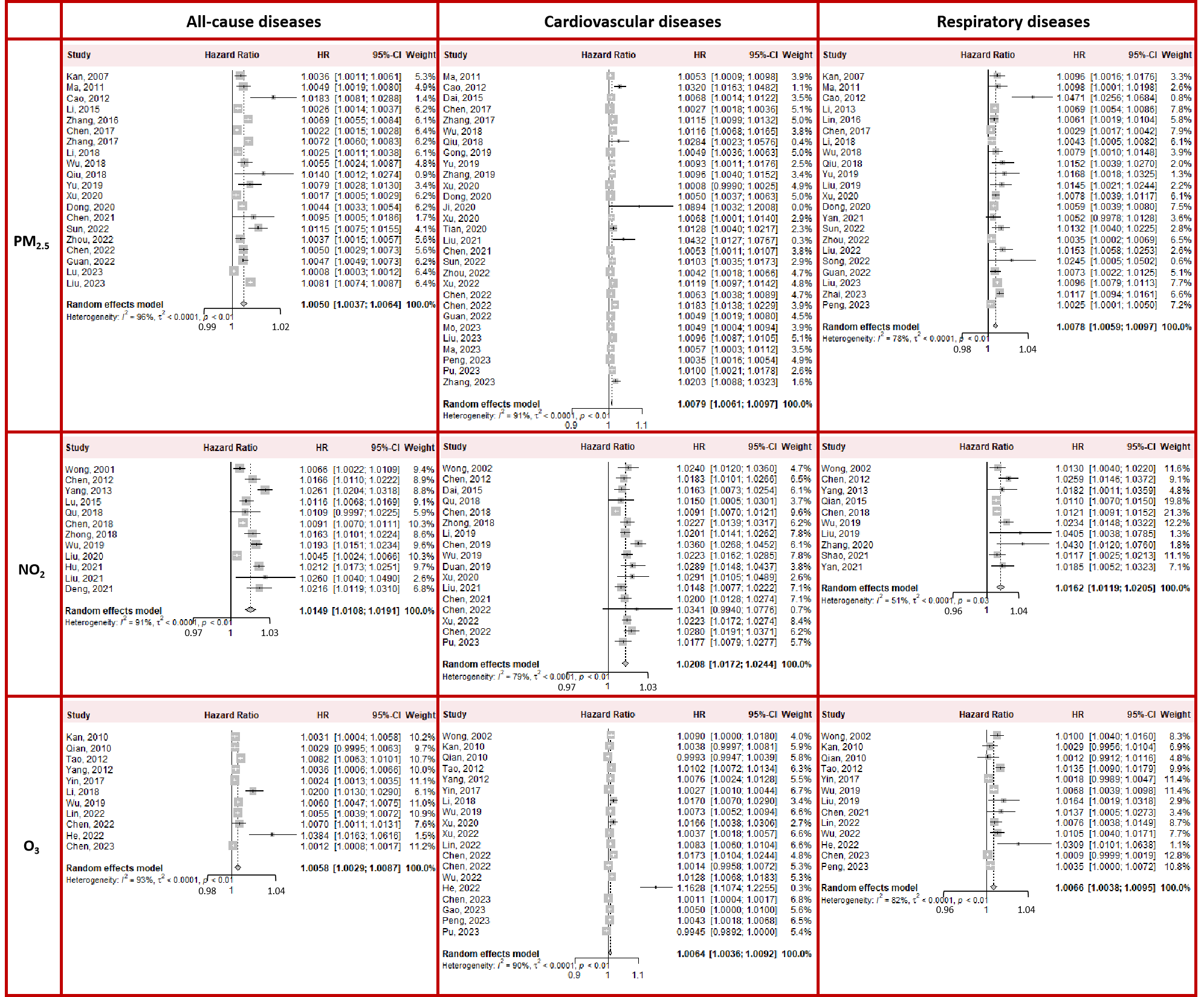


Long-term exposure effect of PM_2.5_, NO_2_ and O_3_ on cause-specific mortality


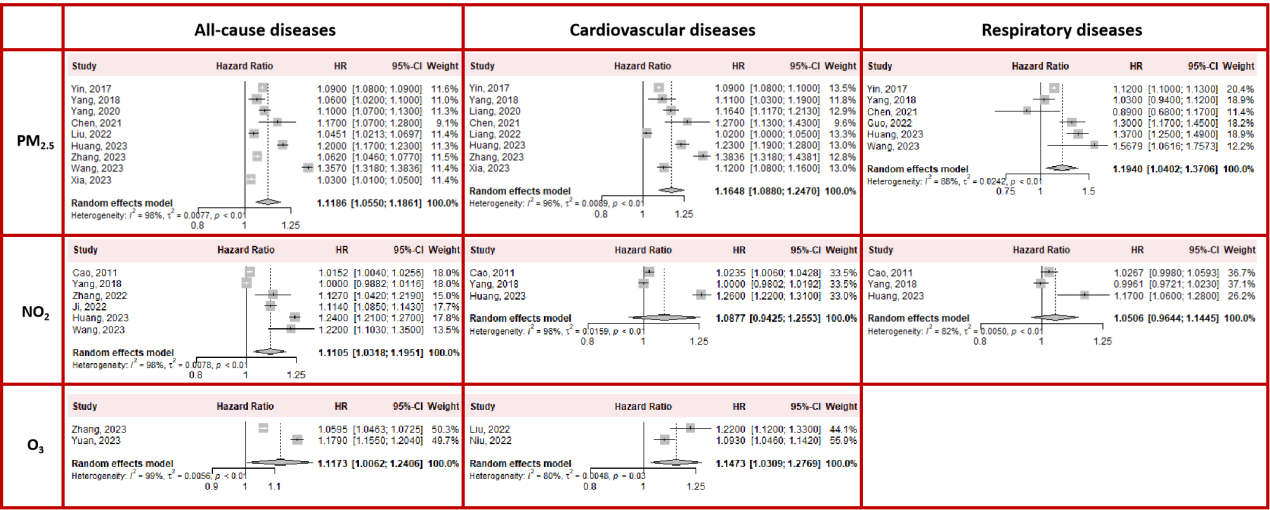


**Appendix 9: Short- and long-term exposure effects of PM_2.5_, NO_2_, and O_3_ on cause-specific mortality in age and gender sub-groups**

The meta-analysis differences in exposure effect among age and gender sub-groups (integrated RR value with 95% CI) are shown in Table 2. There exist exposure effect differences among age sub-groups between elderly (≥65) and young (<65). For long-term effects, except for the exposure effect of NO_2_ on respiratory mortality, these age-related differences in exposure effects are not statistically significant (p-for-interaction>0.05). For short-term effects, the elderly consistently shows higher exposure effects from PM_2.5_ and NO_2_. Except for the non-significant age stratification in NO_2_ exposure effect on all-cause mortality, in all other cases, the exposure effect differences attributed to age are statistically significant (p<0.05). As for the differences in exposure effects among gender sub-groups, no statistically significant differences were found between males and females.

| **Pollutant** | **Period** | **Outcomes** | **Age** | | | **Gender** | | |
| --- | --- | --- | --- | --- | --- | --- | --- | --- |
|  |  |  | **Elderly** | **Young** | **p-value** | **Male** | **Female** | **p-value** |
| PM_2.5_ | L | All-cause mortality | 1.13 (1.02-1.24) | 1.14 (1.04-1.25) | 0.88 | 1.14 (1.08-1.21) | 1.12 (1.03-1.22) | 0.67 |
|  |  | Cardiovascular mortality | 1.23 (1.08-1.41) | 1.24 (1.10-1.40) | 0.95 | 1.21 (1.13-1.29) | 1.22 (1.11-1.35) | 0.83 |
|  |  | Respiratory mortality | 1.37 (0.99-1.89) | 1.41 (1.29-1.53) | 0.87 | 1.22 (1.03-1.44) | 1.35 (0.92-1.98) | 0.63 |
|  | S | All-cause mortality | 1.0068 (1.0044-1.0092) | 1.0002 (0.9983-1.0022) | 0.00 | 1.0044 (1.0026-1.0063) | 1.0065 (1.0043-1.0088) | 0.16 |
|  |  | Cardiovascular mortality | 1.0064 (1.0042-1.0086) | 1.0022 (1.0012-1.0031) | 0.00 | 1.0067 (1.0034-1.0100) | 1.0072 (1.0040-1.0103) | 0.84 |
|  |  | Respiratory mortality | 1.0059 (1.0042-1.0077) | 1.0029 (1.0015-1.0043) | 0.01 | 1.0058 (1.0037-1.0080) | 1.0056 (1.0030-1.0083) | 0.92 |
| NO_2_ | L | All-cause mortality | 1.13 (1.03-1.25) | 1.20 (1.08-1.32) | 0.47 | 1.20 (1.12-1.28) | 1.13 (1.06-1.22) | 0.26 |
|  |  | Cardiovascular mortality | 1.12 (0.89-1.41) | 1.29 (1.24-1.34) | 0.24 | 1.15 (0.90-1.48) | 1.11 (0.96-1.30) | 0.82 |
|  |  | Respiratory mortality | 1.02 (0.93-1.11) | 1.21 (1.10-1.34) | 0.01 | 1.12 (0.99-1.28) | 1.22 (1.07-1.40) | 0.37 |
|  | S | All-cause mortality | 1.0153 (1.0103-1.0204) | 1.0100 (1.0042-1.0157) | 0.17 | 1.0132 (1.0084-1.0182) | 1.0165 (1.0119-1.0211) | 0.35 |
|  |  | Cardiovascular mortality | 1.0211 (1.0149-1.0274) | 1.0061 (1.0024-1.0098) | 0.00 | 1.0161 (1.0092-1.0231) | 1.0215 (1.0123-1.0308) | 0.36 |
|  |  | Respiratory mortality | 1.0130 (1.0102-1.0159) | 1.0060 (1.0008-1.0113) | 0.02 | 1.0113 (1.0080-1.0146) | 1.0138 (1.0082-1.0195) | 0.44 |
| O_3_ | L | All-cause mortality | 1.12 (1.00-1.24) | 1.18 (1.10-1.26) | 0.43 | 1.11 (0.99-1.24) | 1.12 (1.01-1.24) | 0.94 |
|  |  | Cardiovascular mortality | 1.13 (1.05-1.22) | 1.14 (0.97-1.35) | 0.91 | 1.11 (1.05-1.17) | 1.18 (1.00-1.39) | 0.49 |
|  |  | Respiratory mortality | - | - | - | - | - | - |
|  | S | All-cause mortality | 1.0033 (1.0019-1.0047) | 1.0018 (1.0000-1.0037) | 0.22 | 1.0025 (1.0007-1.0043) | 1.0037 (1.0016-1.0058) | 0.40 |
|  |  | Cardiovascular mortality | 1.0019 (0.9999-1.0040) | 1.0003 (0.9993-1.0014) | 0.17 | 1.0043 (0.9985-1.0102) | 1.0016 (0.9997-1.0035) | 0.38 |
|  |  | Respiratory mortality | 1.0003 (0.9995-1.0012) | 1.0038 (0.9983-1.0093) | 0.22 | 1.0014 (1.0002-1.0026) | 1.0034 (0.9909-1.0160) | 0.76 |

**Appendix 10: Details of the ROB assessment**

Out of the 243 articles requiring evaluation, a total of 15 were deemed to have high or higher bias risk (9 with high risk and 6 with very high risk), 102 were assessed to have concerns-level bias risk, and 126 were considered to have low bias risk. Regarding bias due to confounding, 12 were identified as having high risk, with an additional 3 with concerns-level bias risk, primarily due to insufficient consideration of confounding factors. Regarding bias arising from measurement of the exposure, 11 articles were flagged with concerns-level bias risk, mainly because of inadequate explanation of exposure assessment methods. All studies were found to have low bias risk in bias in selection of participants into the study and bias due to post-exposure interventions. Regarding bias due to missing data, 6 were deemed to have high risk, and 40 had concerns-level bias risk, mainly due to extensive missing data, uncertain methods of data imputation, and lack of relevant explanations or considerations. Regarding bias in measurement of the outcome, 18 articles were flagged with concerns-level bias risk, primarily due to the absence of specific ICD codes for health outcomes. Regarding bias in selection of the reported result, 43 articles were flagged with concerns-level bias risk, mainly because observational studies typically lack a planned analysis process, and the reported numerical results may be selected from multiple data analyses, with no a priori knowledge of which analysis the authors would prioritize reporting.

Overall, the included studies showed low and moderate (with concerns) ROB in the assessment according to the ROBINS-E framework. Among them, about 2/3 of the literature was categorized as having concerns, while only 1/3 were considered to have a relatively lower risk. Risks mainly stem from missing data, uncertainty in determining health outcomes, and selective reporting, which do not threaten the accuracy of the results. We also assessed the direction of bias occurrence. Regarding missing data, the random nature of data loss makes it impossible to determine whether exposure effects are biased toward high or low concentrations. Concerning observation bias in health outcomes, since most outcomes lacking ICD coding are declared all-cause, non-accidental, or natural mortality, the direction of bias in effects cannot be determined. In selective reporting, there is usually a tendency to report more significant effects, indicating a bias towards larger effect sizes. However, despite this, no concerns threatening the accuracy of the results were found; hence, they can be considered relatively reliable.

1. Details of the ROB assessment for cohort studies

| **Authors** | **Bias due to confounding** | **Bias arising from measurement of the exposure** | **Bias in selection of participants into the study** | **Bias due to post-exposure interventions** | **Bias due to missing data** | **Bias in measurement of the outcome** | **Bias in selection of the reported result** | **Summary** | **Cite** |
| --- | --- | --- | --- | --- | --- | --- | --- | --- | --- |
| Huang et al. | Low risk | Low risk | Low risk | Low risk | Low risk | Low risk | Low risk | Low risk | (Huang et al., 2023) |
| Zhang et al. | Low risk | Low risk | Low risk | Low risk | Low risk | Concerns | Low risk | Concerns | (Zhang et al., 2022a) |
| Wang et al. | Low risk | Low risk | Low risk | Low risk | Low risk | Concerns | Low risk | Concerns | (Wang et al., 2023d) |
| Ji et al. | Low risk | Low risk | Low risk | Low risk | Low risk | Concerns | Low risk | Concerns | (Ji et al., 2022) |
| Cao et al. | Low risk | Low risk | Low risk | Low risk | Low risk | Low risk | Concerns | Concerns | (Cao et al., 2011) |
| Yang et al. | Low risk | Low risk | Low risk | Low risk | Low risk | Low risk | Concerns | Concerns | (Yang et al., 2018) |
| Zhang et al. | Low risk | Low risk | Low risk | Low risk | Low risk | Concerns | Low risk | Concerns | (Zhang et al., 2023b) |
| Yuan et al. | Low risk | Low risk | Low risk | Low risk | Low risk | Concerns | Low risk | Concerns | (Yuan et al., 2023) |
| Liu et al. | Low risk | Low risk | Low risk | Low risk | Low risk | Low risk | Low risk | Low risk | (Liu et al., 2022b) |
| Niu et al. | Low risk | Low risk | Low risk | Low risk | Low risk | Low risk | Concerns | Concerns | (Niu et al., 2022) |
| Liu et al. | Low risk | Low risk | Low risk | Low risk | Low risk | Concerns | Low risk | Concerns | (Liu et al., 2022a) |
| Yang et al. | Low risk | Low risk | Low risk | Low risk | Low risk | Concerns | Low risk | Concerns | (Yang et al., 2020) |
| Yin et al. | Low risk | Low risk | Low risk | Low risk | Low risk | Low risk | Concerns | Concerns | (Yin et al., 2017a) |
| Wang et al. | Low risk | Low risk | Low risk | Low risk | Low risk | Concerns | Low risk | Concerns | (Wang et al., 2023e) |
| Xia et al. | Low risk | Low risk | Low risk | Low risk | Low risk | Low risk | Concerns | Concerns | (Xia et al., 2023) |
| Chen et al. | Low risk | Low risk | Low risk | Low risk | Low risk | Low risk | Low risk | Low risk | (Chen et al., 2021c) |
| Liang et al. | Low risk | Low risk | Low risk | Low risk | Low risk | Low risk | Concerns | Concerns | (Liang et al., 2022) |
| Liang et al. | Low risk | Low risk | Low risk | Low risk | Low risk | Low risk | Low risk | Low risk | (Liang et al., 2020) |
| Zhang et al. | Low risk | Low risk | Low risk | Low risk | Low risk | Low risk | Concerns | Concerns | (Zhang et al., 2023a) |
| Guo et al. | Low risk | Low risk | Low risk | Low risk | Low risk | Low risk | Concerns | Concerns | (Guo et al., 2022) |
| Wang et al. | Low risk | Low risk | Low risk | Low risk | Low risk | Low risk | Concerns | Concerns | (Wang et al., 2023c) |

1. Details of the ROB assessment for time-series and case-crossover studies

| **Authors** | **Bias due to confounding** | **Bias arising from measurement**  **of the exposure** | **Bias in selection of participants**  **into the study** | **Bias due to post-exposure**  **interventions** | **Bias due to missing data** | **Bias in measurement**  **of the outcome** | **Bias in selection of the**  **reported result** | **Summary** | **Cite** |
| --- | --- | --- | --- | --- | --- | --- | --- | --- | --- |
| Yang et al. | Low risk | Low risk | Low risk | Low risk | Concerns | Low risk | Low risk | Concerns | (Yang et al., 2013) |
| Qu et al. | Low risk | Low risk | Low risk | Low risk | Low risk | Low risk | Concerns | Concerns | (Qu et al., 2018) |
| Chen et al. | Low risk | Low risk | Low risk | Low risk | Low risk | Low risk | Low risk | Low risk | (Chen et al., 2012) |
| Hu et al. | Low risk | Low risk | Low risk | Low risk | Low risk | Concerns | Low risk | Concerns | (Hu et al., 2021) |
| Chen et al. | Low risk | Low risk | Low risk | Low risk | Low risk | Low risk | Concerns | Concerns | (Chen et al., 2018b) |
| Wu et al. | Low risk | Low risk | Low risk | Low risk | Low risk | Low risk | Low risk | Low risk | (Wu et al., 2019a) |
| Liu et al. | Low risk | Low risk | Low risk | Low risk | Concerns | Low risk | Low risk | Concerns | (Liu et al., 2021a) |
| Deng et al. | Low risk | Low risk | Low risk | Low risk | Low risk | Low risk | Low risk | Low risk | (Deng et al., 2021) |
| Wong et al. | Low risk | Low risk | Low risk | Low risk | Low risk | Low risk | Low risk | Low risk | (Wong et al., 2001) |
| Liu et al. | Low risk | Low risk | Low risk | Low risk | Low risk | Low risk | Low risk | Low risk | (Liu et al., 2020) |
| Lu et al. | Low risk | Low risk | Low risk | Low risk | Concerns | Low risk | Low risk | Concerns | (Lu et al., 2015) |
| Zhong et al. | Low risk | Low risk | Low risk | Low risk | Low risk | Low risk | Concerns | Concerns | (Zhong et al., 2018) |
| Li et al. | Low risk | Low risk | Low risk | Low risk | Concerns | Low risk | Low risk | Concerns | (Li et al., 2019a) |
| Chen et al. | Low risk | Low risk | Low risk | Low risk | Concerns | Low risk | Low risk | Concerns | (Chen et al., 2019c) |
| Chen et al. | Low risk | Low risk | Low risk | Low risk | Concerns | Low risk | Low risk | Concerns | (Chen et al., 2022d) |
| Xu et al. | Low risk | Low risk | Low risk | Low risk | Concerns | Low risk | Low risk | Concerns | (Xu et al., 2020a) |
| Wong et al. | Low risk | Low risk | Low risk | Low risk | Concerns | Low risk | Low risk | Concerns | (Wong et al., 2002) |
| Liu et al. | Low risk | Low risk | Low risk | Low risk | Concerns | Low risk | Low risk | Concerns | (Liu et al., 2021b) |
| Xu et al. | Low risk | Low risk | Low risk | Low risk | Low risk | Low risk | Concerns | Concerns | (Xu et al., 2022) |
| Chen et al. | Low risk | Low risk | Low risk | Low risk | Concerns | Low risk | Low risk | Concerns | (Chen et al., 2022b) |
| Chen et al. | Low risk | Low risk | Low risk | Low risk | Concerns | Low risk | Low risk | Concerns | (Chen et al., 2021b) |
| Dai et al. | Low risk | Low risk | Low risk | Low risk | Concerns | Low risk | Low risk | Concerns | (Dai et al., 2015) |
| Duan et al. | Low risk | Low risk | Low risk | Low risk | Low risk | Low risk | Concerns | Concerns | (Duan et al., 2019) |
| Pu et al. | Low risk | Low risk | Low risk | Low risk | Low risk | Low risk | Concerns | Concerns | (Pu et al., 2023) |
| Shao et al. | Low risk | Low risk | Low risk | Low risk | Concerns | Low risk | Low risk | Concerns | (Shao et al., 2021) |
| Liu et al. | Low risk | Low risk | Low risk | Low risk | Concerns | Low risk | Low risk | Concerns | (Liu et al., 2019d) |
| Qian et al. | Low risk | Concerns | Low risk | Low risk | Low risk | Low risk | Low risk | Concerns | (Qian et al., 2015) |
| Yan et al. | Low risk | Low risk | Low risk | Low risk | Low risk | Low risk | Concerns | Concerns | (Yan et al., 2021) |
| Zhang et al. | Low risk | Low risk | Low risk | Low risk | Concerns | Low risk | Low risk | Concerns | (Zhang et al., 2020b) |
| Li et al. | Low risk | Low risk | Low risk | Low risk | Low risk | Low risk | Low risk | Low risk | (Liu et al., 2018) |
| Yin et al. | Low risk | Low risk | Low risk | Low risk | Low risk | Low risk | Low risk | Low risk | (Yin et al., 2017b) |
| Chen et al. | Low risk | Low risk | Low risk | Low risk | Concerns | Low risk | Low risk | Concerns | (Chen et al., 2023a) |
| Lin et al. | Low risk | Low risk | Low risk | Low risk | Concerns | Low risk | Low risk | Concerns | (Lin et al., 2022) |
| Chen et al. | Low risk | Low risk | Low risk | Low risk | Concerns | Low risk | Low risk | Concerns | (Chen et al., 2022a) |
| He et al. | Low risk | Low risk | Low risk | Low risk | Concerns | Low risk | Low risk | Concerns | (He, 2022) |
| Tao et al. | Low risk | Low risk | Low risk | Low risk | Low risk | Low risk | Low risk | Low risk | (Tao et al., 2012) |
| Kan et al. | Low risk | Low risk | Low risk | Low risk | Low risk | Low risk | Low risk | Low risk | (Kan et al., 2010) |
| Yang et al. | Low risk | Low risk | Low risk | Low risk | Low risk | Low risk | Low risk | Low risk | (Yang et al., 2012b) |
| Qian et al. | Low risk | Low risk | Low risk | Low risk | Concerns | Low risk | Low risk | Concerns | (Qian et al., 2010) |
| Wu et al. | Low risk | Low risk | Low risk | Low risk | Concerns | Low risk | Low risk | Concerns | (Wu et al., 2022) |
| Gao et al. | Low risk | Low risk | Low risk | Low risk | Low risk | Low risk | Low risk | Low risk | (Gao et al., 2023) |
| Peng et al. | Low risk | Low risk | Low risk | Low risk | Concerns | Low risk | Low risk | Concerns | (Peng, 2023) |
| Chen et al. | Low risk | Low risk | Low risk | Low risk | Low risk | Low risk | Low risk | Low risk | (Chen et al., 2021d) |
| Lu et al. | Low risk | Low risk | Low risk | Low risk | Concerns | Low risk | Low risk | Concerns | (Lu et al., 2023) |
| Xu et al. | Low risk | Low risk | Low risk | Low risk | Concerns | Low risk | Low risk | Concerns | (Xu et al., 2020b) |
| Li et al. | Low risk | Low risk | Low risk | Low risk | Low risk | Low risk | Low risk | Low risk | (Li et al., 2015a) |
| Li et al. | Low risk | Low risk | Low risk | Low risk | Low risk | Low risk | Low risk | Low risk | (Li et al., 2018b) |
| Yu et al. | Low risk | Low risk | Low risk | Low risk | Low risk | Low risk | Concerns | Concerns | (Yu et al., 2019) |
| Sun et al. | Low risk | Low risk | Low risk | Low risk | Concerns | Low risk | Low risk | Concerns | (Sun et al., 2022) |
| Chen et al. | Low risk | Low risk | Low risk | Low risk | Concerns | Low risk | Low risk | Concerns | (Chen et al., 2017b) |
| Zhou et al. | Low risk | Low risk | Low risk | Low risk | Low risk | Low risk | Low risk | Low risk | (Zhou et al., 2022) |
| Dong et al. | Low risk | Low risk | Low risk | Low risk | Low risk | Low risk | Low risk | Low risk | (Dong et al., 2020) |
| Liu et al. | Low risk | Low risk | Low risk | Low risk | Low risk | Low risk | Low risk | Low risk | (Liu et al., 2023b) |
| Wu et al. | Low risk | Low risk | Low risk | Low risk | Low risk | Low risk | Low risk | Low risk | (Wu et al., 2018) |
| Qiu et al. | Low risk | Low risk | Low risk | Low risk | Low risk | Low risk | Concerns | Concerns | (Qiu et al., 2018) |
| Chen et al. | Low risk | Low risk | Low risk | Low risk | Low risk | Low risk | Low risk | Low risk | (Chen et al., 2022c) |
| Zhang et al. | Low risk | Low risk | Low risk | Low risk | Concerns | Low risk | Low risk | Concerns | (Zhang et al., 2017b) |
| Kan et al. | Low risk | Low risk | Low risk | Low risk | Low risk | Low risk | Low risk | Low risk | (Kan et al., 2007) |
| Ma et al. | Low risk | Low risk | Low risk | Low risk | Low risk | Low risk | Low risk | Low risk | (Ma et al., 2011) |
| Zhang et al. | Low risk | Low risk | Low risk | Low risk | Low risk | Low risk | Low risk | Low risk | (Zhang et al., 2016) |
| Guan et al. | Low risk | Concerns | Low risk | Low risk | Concerns | Low risk | Low risk | Concerns | (Guan et al., 2022) |
| Cao et al. | Low risk | Low risk | Low risk | Low risk | Low risk | Low risk | Low risk | Low risk | (Cao et al., 2012) |
| Gong et al. | Low risk | Low risk | Low risk | Low risk | Low risk | Low risk | Low risk | Low risk | (Gong et al., 2019a) |
| Mo et al. | Low risk | Low risk | Low risk | Low risk | Low risk | Low risk | Concerns | Concerns | (Mo et al., 2023) |
| Ji et al. | Low risk | Low risk | Low risk | Low risk | Concerns | Low risk | Low risk | Concerns | (Ji et al., 2020b) |
| Ma et al. | Low risk | Low risk | Low risk | Low risk | Concerns | Low risk | Low risk | Concerns | (Ma et al., 2023) |
| Tian et al. | Low risk | Low risk | Low risk | Low risk | Low risk | Low risk | Concerns | Concerns | (Tian et al., 2020b) |
| Zhang et al. | Low risk | Concerns | Low risk | Low risk | Low risk | Low risk | Low risk | Concerns | (Zhang, 2023) |
| Zhang et al. | Low risk | Low risk | Low risk | Low risk | Concerns | Low risk | Low risk | Concerns | (Zhang, 2019b) |
| Li et al. | Low risk | Low risk | Low risk | Low risk | Low risk | Low risk | Low risk | Low risk | (Li et al., 2013a) |
| Liu et al. | Low risk | Low risk | Low risk | Low risk | Concerns | Low risk | Low risk | Concerns | (Liu et al., 2022c) |
| Lin et al. | Low risk | Low risk | Low risk | Low risk | Low risk | Low risk | Low risk | Low risk | (Lin et al., 2016b) |
| Song et al. | Low risk | Low risk | Low risk | Low risk | Concerns | Low risk | Low risk | Concerns | (Song et al., 2022) |
| Zhai et al. | Low risk | Low risk | Low risk | Low risk | Low risk | Low risk | Low risk | Low risk | (Zhai et al., 2023) |

**Appendix 11: Results of the symmetry assessment of the funnel plot**

In Egger's test, only the short-term exposure of PM_2.5_ to all-cause and cardiovascular mortality effects exhibited significant asymmetry in the funnel plot distribution at a 0.05 significance level (p=0.14 and 0.09), while the funnel distribution of other effects showed symmetry with confidence (p=<0.01-0.04). However, in Begg's test, except for the short-term exposure of PM_2.5_ to cardiovascular and respiratory mortality effects, which did not show significant asymmetry in the funnel plot distribution (p=<0.005 and 0.02), the remaining effects demonstrated significant asymmetry (p=0.15-0.93). Both test results reflected a certain degree of publication bias among the included literature.

| **Pollutant** | **Period** | **Health Endpoint** | **Begg's test: p-value** | **Egger's test: p-value** |
| --- | --- | --- | --- | --- |
| PM_2.5_ | L | All-cause deaths | - | - |
|  |  | Cardiovascular mortality | - | - |
|  |  | Respiratory diseases | - | - |
|  | S | All-cause deaths | 0.1534 | 0.1364 |
|  |  | Cardiovascular mortality | 0.0039 | 0.0957 |
|  |  | Respiratory diseases | 0.0165 | 0.0127 |
| NO_2_ | L | All-cause deaths | - | - |
|  |  | Cardiovascular mortality | - | - |
|  |  | Respiratory diseases | - | - |
|  | S | All-cause deaths | 0.5833 | 0.0388 |
|  |  | Cardiovascular mortality | 0.9343 | 0.0015 |
|  |  | Respiratory diseases | 0.1797 | 0.0084 |
| O_3_ | L | All-cause deaths | - | - |
|  |  | Cardiovascular mortality | - | - |
|  |  | Respiratory diseases | - | - |
|  | S | All-cause deaths | 0.3115 | 0.0061 |
|  |  | Cardiovascular mortality | 0.1515 | 0.0022 |
|  |  | Respiratory diseases | 0.5418 | 0.0024 |

**Appendix 12: Results of the meta-regression analysis**

The substantial heterogeneity in the literature suggests the presence of study characteristics that influence the final quantitative results. We conducted meta-regression analyses to assess the impact of different factors on the heterogeneity of the meta-analysis. The publication year, study population, pollution simulation resolution, and exposure level were the top contributing factors to heterogeneity. The contribution of publication year may be attributed to methodological improvements and disciplinary advancements over time, indicating the necessity of including recent literature and excluding older literature. The heterogeneity between adult cohorts and middle-aged or elderly cohorts in the study population is quite significant. There are differences in the representativeness of middle-aged or elderly compared to the whole adult population. Further accumulation of relevant literature on adult populations is needed. Study design, study region, sampling size, and improvements in exposure assessment methods also contributed to some extent of heterogeneity, although generally not significantly. Overall, the sources of heterogeneity are diverse and complex, and each factor examined in the meta-regression may impact the integration results.

There are fewer than five studies on the long-term exposure effects of NO_2_ and O_3_, so the meta-regression was not conducted. In some groups, some factors did not achieve two or more layers, so the regression was also not performed for them.

| **Pollutant** | **Period** | **Outcomes** | **Publication Year** | | **Study design** | | **Area type** | | **Population Type** | | **Population Size** | | **Death Size** | | **Assessment Methods** | | **Resolution** | | **Pollutant Level** | |
| --- | --- | --- | --- | --- | --- | --- | --- | --- | --- | --- | --- | --- | --- | --- | --- | --- | --- | --- | --- | --- |
|  |  |  | **ΔI^2^** | **R^2^** | **ΔI^2^** | **R^2^** | **ΔI^2^** | **R^2^** | **ΔI^2^** | **R^2^** | **ΔI^2^** | **R^2^** | **ΔI^2^** | **R^2^** | **ΔI^2^** | **R^2^** | **ΔI^2^** | **R^2^** | **ΔI^2^** | **R^2^** |
| PM_2.5_ | L | All-cause mortality | 0.99 | 0.00 | - | - | 0.99 | 0.00 | 0.99 | 0.20 | 0.99 | 0.00 | 0.98 | 0.00 | 0.99 | 0.03 | 0.98 | 0.00 | 0.99 | 0.00 |
|  |  | Cardiovascular mortality | 0.97 | 0.00 | - | - | 0.97 | 0.00 | 0.87 | 0.77 | 0.98 | 0.00 | 0.97 | 0.00 | - | - | 0.97 | 0.05 | 0.98 | 0.00 |
|  |  | Respiratory mortality | 0.88 | 0.27 | - | - | 0.92 | 0.00 | 0.88 | 0.45 | 0.89 | 0.04 | 0.00 | 1.00 | - | - | 0.00 | 1.00 | 0.91 | 0.00 |
|  | S | All-cause mortality | 0.94 | 0.00 | 0.95 | 0.00 | 0.95 | 0.00 | 0.95 | 0.03 | - | - | 0.95 | 0.00 | 0.96 | 0.00 | 0.94 | 0.01 | 0.94 | 0.00 |
|  |  | Cardiovascular mortality | 0.91 | 0.01 | 0.92 | 0.00 | 0.92 | 0.00 | 0.92 | 0.00 | - | - | 0.88 | 0.00 | 0.91 | 0.00 | 0.93 | 0.00 | 0.92 | 0.00 |
|  |  | Respiratory mortality | 0.91 | 0.37 | 0.78 | 0.14 | 0.79 | 0.00 | 0.81 | 0.00 | - | - | 0.75 | 0.00 | 0.80 | 0.00 | 0.82 | 0.00 | 0.78 | 0.03 |
| NO_2_ | L | All-cause mortality | 0.97 | 0.56 | - | - | 0.98 | 0.00 | 0.93 | 0.83 | 0.98 | 0.33 | 0.89 | 0.47 | 0.99 | 0.00 | 0.81 | 0.52 | 0.98 | 0.16 |
|  |  | Cardiovascular mortality | - | - | - | - | - | - | - | - | - | - | - | - | - | - | - | - | - | - |
|  |  | Respiratory mortality | - | - | - | - | - | - | - | - | - | - | - | - | - | - | - | - | - | - |
|  | S | All-cause mortality | 0.86 | 0.12 | - | - | 0.91 | 0.00 | - | - | - | - | 0.87 | 0.14 | 0.92 | 0.00 | 0.91 | 0.00 | 0.87 | 0.00 |
|  |  | Cardiovascular mortality | 0.72 | 0.00 | 0.72 | 0.00 | 0.13 | 0.28 | - | - | - | - | 0.51 | 0.00 | - | - | 0.73 | 0.00 | 0.70 | 0.01 |
|  |  | Respiratory mortality | 0.02 | 0.99 | 0.17 | 0.85 | 0.40 | 0.52 | - | - | - | - | 0.36 | 0.04 | - | - | 0.57 | 0.10 | 0.09 | 0.86 |
| O_3_ | L | All-cause mortality | - | - | - | - | - | - | - | - | - | - | - | - | - | - | - | - | - | - |
|  |  | Cardiovascular mortality | - | - | - | - | - | - | - | - | - | - | - | - | - | - | - | - | - | - |
|  |  | Respiratory mortality | - | - | - | - | - | - | - | - | - | - | - | - | - | - | - | - | - | - |
|  | S | All-cause mortality | 0.95 | 0.00 | 0.97 | 0.00 | 0.98 | 0.00 | - | - | - | - | 0.96 | 0.00 | 0.96 | 0.00 | 0.96 | 0.00 | 0.97 | 0.00 |
|  |  | Cardiovascular mortality | 0.94 | 0.00 | 0.93 | 0.00 | 0.94 | 0.00 | - | - | - | - | 0.96 | 0.00 | - | - | 0.94 | 0.00 | 0.93 | 0.03 |
|  |  | Respiratory mortality | 0.70 | 0.31 | 0.69 | 0.33 | 0.82 | 0.00 | - | - | - | - | 0.89 | 0.00 | - | - | 0.77 | 0.00 | 0.78 | 0.15 |

**Appendix 13: Sensitivity Analysis**

- - - 1. **Results of the leave-one-out validation**

In the leave-one-out validation, the mean integrated effects (with corresponding fluctuation intervals) for long-term exposure to PM_2.5_ on all-cause, cardiovascular, and respiratory mortality are 1.12 (1.09-1.13), 1.16 (1.13-1.19), and 1.17 (1.12-1.21), respectively. Excluding individual studies had a non-significant impact on the integrated effects at the levels of 0.001, 0.0001, and 0.05, respectively. For NO_2_ exposure, only the long-term effect on all-cause mortality was non-significantly affected in the leave-one-out validation, while the impact on cardiovascular and respiratory mortality effects was significant (p>0.05). Despite the insignificant impacts at the level of 0.05 on the long-term integrated effects of O_3_, there are still relatively wider fluctuation intervals than PM_2.5_ due to the literature limitation and high heterogeneity. The leave-one-out validation results were all insignificant for the three pollutants' short-term exposure effects. Overall, the integrated effects of meta-analysis are proven to be robust.

| **Pollutant** | **Period** | **Health Endpoint** | **Pooled Estimate** | **Min effect after omitting** | **Max effect after omitting** | **p-value** |
| --- | --- | --- | --- | --- | --- | --- |
| PM_2.5_ | L | All-cause deaths | 1.1186 | 1.0889 | 1.1306 | <0.001 |
|  |  | Cardiovascular mortality | 1.1648 | 1.1324 | 1.1882 | <0.0001 |
|  |  | Respiratory diseases | 1.1722 | 1.1251 | 1.2127 | <0.05 |
|  | S | All-cause deaths | 1.005 | 1.0047 | 1.0053 | <0.0001 |
|  |  | Cardiovascular mortality | 1.0079 | 1.0079 | 1.0081 | <0.0001 |
|  |  | Respiratory diseases | 1.0078 | 1.0072 | 1.0081 | <0.0001 |
| NO_2_ | L | All-cause deaths | 1.1105 | 1.0813 | 1.1357 | <0.05 |
|  |  | Cardiovascular mortality | 1.0877 | 1.012 | 1.135 | >0.05 |
|  |  | Respiratory diseases | 1.0506 | 1.0102 | 1.0873 | >0.05 |
|  | S | All-cause deaths | 1.0149 | 1.0142 | 1.016 | <0.0001 |
|  |  | Cardiovascular mortality | 1.0208 | 1.0206 | 1.0217 | <0.0001 |
|  |  | Respiratory diseases | 1.0162 | 1.0129 | 1.0176 | <0.0001 |
| O_3_ | L | All-cause deaths | 1.1173 | 1.0595 | 1.179 | <0.05 |
|  |  | Cardiovascular mortality | 1.1473 | 1.0930 | 1.22 | <0.05 |
|  |  | Respiratory diseases | - | - | - | - |
|  | S | All-cause deaths | 1.0058 | 1.0045 | 1.0068 | <0.0001 |
|  |  | Cardiovascular mortality | 1.0064 | 1.0056 | 1.0069 | <0.0001 |
|  |  | Respiratory diseases | 1.0066 | 1.0056 | 1.0074 | <0.0001 |

- - - 1. **Results of the comparison with different confounder selection**

Sensitivity analysis indicates that the results obtained from models adjusted for relatively consistent confounding factors do not differ significantly compared with those derived from models with variations between studies. It indicates that the differences in details between studies do not lead to instability in the integrated results of exposure effects.

| **Pollutant** | **Outcomes** | **Studies** | **RR** | **95%CI_lower** | **95%CI_upper** | **p-value compared**  **with original result** |
| --- | --- | --- | --- | --- | --- | --- |
| PM_2.5_ | All-cause mortality | 9 | 1.12 | 1.06 | 1.19 | 1 |
|  | Cardiovascular mortality | 8 | 1.16 | 1.09 | 1.25 | 1 |
|  | Respiratory mortality | 6 | 1.17 | 1.01 | 1.37 | 0.967 |
| NO_2_ | All-cause mortality | 6 | 1.11 | 1.03 | 1.20 | 1 |
|  | Cardiovascular mortality | 3 | 1.09 | 0.94 | 1.26 | 1 |
|  | Respiratory mortality | 3 | 1.05 | 0.96 | 1.14 | 1 |
| O_3_ | All-cause mortality | 2 | 1.12 | 1.01 | 1.24 | 0.809 |
|  | Cardiovascular mortality | 2 | 1.15 | 1.03 | 1.28 | 1 |

- - - 1. **Results of the sub-group analysis of studies that overlapped with the COVID-19 pandemic or not**

In total, only 8 studies overlapped with the period of the COVID-19 outbreak, with 6 studies extending into 2020 and only 2 extending into 2021. Except for the effect of O_3_ exposure on all-cause mortality (p-for-interaction=0.0035), all other groups of long- and short-term effects did not show significant differences (p-for-interaction=0.12-0.78). The reasons are multifaceted. Firstly, only one study overlapping with the COVID-19 outbreak period was included. Secondly, this study is solely conducted in a single city, with a notably higher pollutant concentration (130 μg/m^3^) than the average concentration in other studies (81 μg/m^3^). The significant disparity between this study and others cannot solely be attributed to the timing of the COVID-19 pandemic. Additionally, it should not be ignored that the study periods of the aforementioned literature all include different lengths of time before the COVID-19 outbreak, and such large samples may weaken or even flatten the impact of COVID-19 on changes in mortality risk. Moreover, during the early stages of the COVID-19 outbreak in China, direct infection among the Chinese population was minimal, and main exposure routes shifted from outdoor to indoor. In brief, despite significant differences observed in one aspect of our study during statistical analysis, we maintain that the existing evidence does not conclusively demonstrate a significant impact of the COVID-19 outbreak on the mortality risk associated with major air pollutants. The quantitative and qualitative conclusions drawn from our study remain robust and reliable.

| **Period** | **Pollutant** | **Outcomes** | **Containing COVID-19 or not** | **Studies** |  | **p-value** |
| --- | --- | --- | --- | --- | --- | --- |
| L | NO_2_ | All-cause mortality | N | 5 | 1.11(1.02-1.21) |  |
|  |  |  | Y | 1 | 1.13(1.04-1.22) | 0.78 |
| S | NO_2_ | Cardiovascular mortality | N | 16 | 1.0203(1.0167-1.0240) |  |
|  |  |  | Y | 1 | 1.0280(1.0191-1.0371) | 0.12 |
|  | O_3_ | All-cause mortality | N | 10 | 1.0051(1.0028-1.0075) |  |
|  |  |  | Y | 1 | 1.0384(1.0163-1.0616) | 0.0035 |
|  |  | Cardiovascular mortality | N | 14 | 1.0051(1.0025-1.0078) |  |
|  |  |  | Y | 5 | 1.0337(0.9836-1.0863) | 0.26 |
|  |  | Respiratory mortality | N | 10 | 1.0064(1.0031-1.0097) |  |
|  |  |  | Y | 3 | 1.0093(0.9998-1.0189) | 0.57 |
|  | PM_2.5_ | All-cause mortality | N | 19 | 1.0051(1.0036-1.0065) |  |
|  |  |  | Y | 1 | 1.0047(1.0049-1.0073) | 0.71 |
|  |  | Cardiovascular mortality | N | 25 | 1.0074(1.0058-1.0091) |  |
|  |  |  | Y | 4 | 1.0108(1.0025-1.0193) | 0.43 |
|  |  | Respiratory mortality | N | 18 | 1.0074(1.0055-1.0092) |  |
|  |  |  | Y | 4 | 1.0083(1.0031-1.0137) | 0.73 |

- - - 1. **Results of the meta-analysis taking multi-pollutant models into consideration**

In the aforementioned studies on NO_2_ and O_3_ exposure effects, all long-term effect studies established multi-pollutant models, while the proportion of short-term effect studies that established multi-pollutant models averaged 67%. In contrast, fewer than 1/3 of the studies on PM_2.5_ established multi-pollutant models.

The results of the sensitivity analysis show that replacing the single-pollutant models used in all RR studies included in the meta-analysis with multi-pollutant models (if available) did not significantly impact the final integrated results statistically (p-value>0.05). The only notable difference was that the previously significant negative effects of NO_2_ and O_3_ exposure on all-cause mortality became non-significant after adjusting for multi-pollutant models (95%CI included RR=1). However, correspondingly, the confidence intervals became wider than those in the single-pollutant models. Similarly, the short-term exposure effects of NO_2_ and O_3_ showed relatively larger changes after applying multi-pollutant model adjustments compared to the robustness of PM_2.5_. These changes may indicate that the mechanisms by which NO_2_ and O_3_ exert adverse exposure effects on the human body are more complex and may involve interactions between multiple pollutants. This result suggests that more evidence is needed to form a more definitive understanding of the exposure effects of pollutants like NO_2_ and O_3_.

Furthermore, studies on the exposure effects of PM_2.5_ components typically only report the total mass concentration and the independent effects of various exposure components, without generally adjusting for gaseous pollutants. Among the 59 studies involving PM_2.5_ exposure effects, only 7 provided health effects related to the complex components of PM_2.5_. Due to the limited number of such studies and the scopes of this paper, we did not further analyze these results.


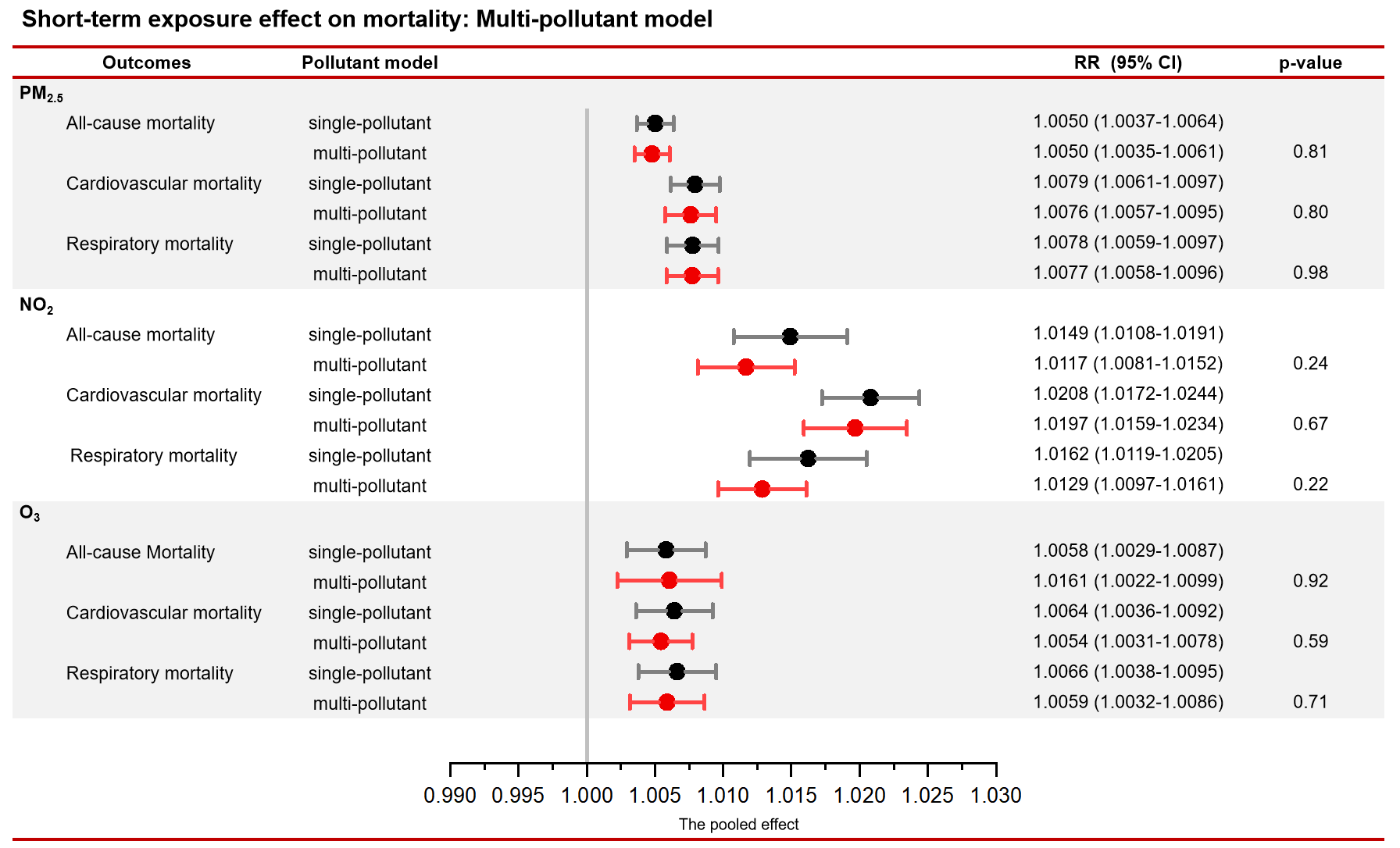


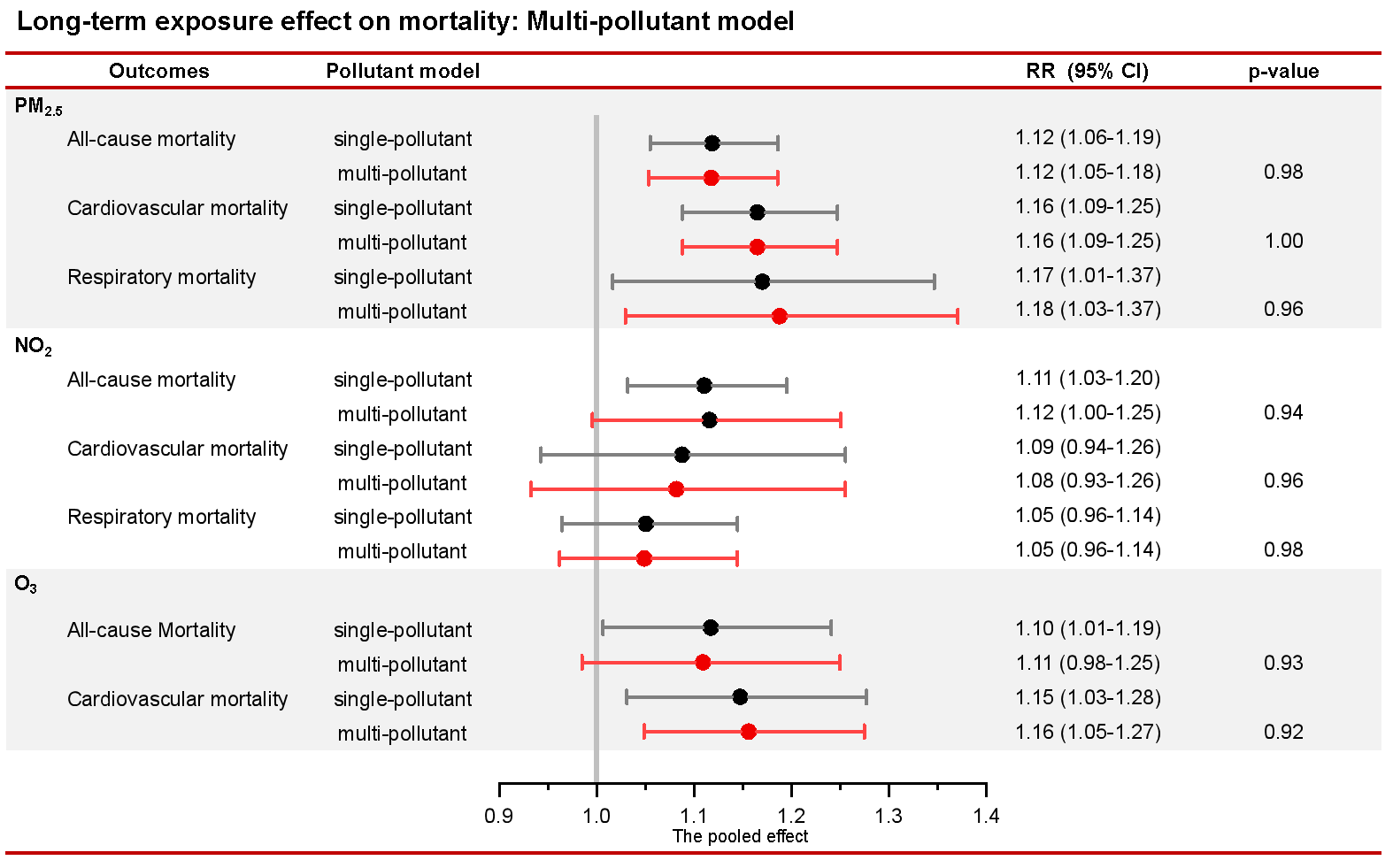


**Appendix 14: Results of the certainty assessment**

Overall, the included literature exhibited a low risk of bias, with all literature addressing the original research question. Adjusting the confounding factors would not potentially change the effect estimates in both directions. The long-term exposure effects of PM_2.5_ and the short-term exposure effects of three pollutants were assessed to have high certainty of evidence. However, the certainty for the long-term exposure effects of NO_2_ to all-cause mortality and O_3_ to cardiovascular mortality was rated as moderate; the long-term exposure effects of O_3_ to all-cause mortality was rated as low; and the long-term exposure effects of NO_2_ to cardiovascular and respiratory mortality was rated as very low. There are several reasons. The existing cohort studies are too limited to judge publication bias. Thus, we failed to exclude potential interference with the results. For most studies on NO_2_ and O_3_, the sampling sizes are relatively small, encompassing only hundreds of thousands of people, below the threshold set by the GRADE criteria for cohort studies. Additionally, the long-term O_3_ exposure effect exhibited an association with the unadjusted confounders, indicating the existence of the potential interactions that might significantly influence the quantitative result and thus lead to uncertainty regarding the exposure-effect association. Furthermore, the integrated effects of NO_2_ on cardiovascular and respiratory mortality are insignificant. The above limitations in existing studies contribute to the lower certainty of evidence for the exposure effects of NO_2_ and O_3_.

| **Exposure-endpoint group** | **Downgrade** |  |  |  |  | **Upgrade** |  |  | **Certainty of evidence** |
| --- | --- | --- | --- | --- | --- | --- | --- | --- | --- |
|  | **Limitations in studies** | **Indirectness** | **Inconsistency** | **Imprecision** | **Publication bias** | **Large effect size** | **Confounding** | **Concentration-response gradient** |  |
| Long-term_PM_2.5__All-cause mortality | (0) No differences between studies with low/moderate versus high RoB | (0) The research question in the studies reflects the original question. | (0) 80% prediction interval did not include unity. | (0) Number of mortality cases higher than 940,000. | (-1) Publication bias was not assessed. | (+1) Unmeasured confounding would not suffice to explain away the effect estimate. | (0)  Several potential confounders that would shift the RR in both directions. | (+1) Significant positive association detected in the main analysis. | ⊗⊗⊗⊗ High |
| Long-term_PM_2.5__Cardiovascular mortality | (0) No differences between studies with low/moderate versus high RoB | (0) The research question in the studies reflects the original question. | (0) 80% prediction interval did not include unity. | (0) Number of mortality cases higher than 940,000. | (-1) Publication bias was not assessed. | (+1) Unmeasured confounding would not suffice to explain away the effect estimate. | (0)  Several potential confounders that would shift the RR in both directions. | (+1) Significant positive association detected in the main analysis. | ⊗⊗⊗⊗ High |
| Long-term_PM_2.5__Respiratory mortality | (0) No differences between studies with low/moderate versus high RoB | (0) The research question in the studies reflects the original question. | (0) 80% prediction interval did not include unity. | (0) Number of mortality cases higher than 940,000. | (-1) Publication bias was not assessed. | (+1) Unmeasured confounding would not suffice to explain away the effect estimate. | (0)  Several potential confounders that would shift the RR in both directions. | (+1) Significant positive association detected in the main analysis. | ⊗⊗⊗⊗ High |
| Long-term_NO_2__All-cause mortality | (0) No differences between studies with low/moderate versus high RoB | (0) The research question in the studies reflects the original question. | (0) 80% prediction interval did not include unity. | (-1) Number of mortality cases lower than 940,000. | (-1) Publication bias was not assessed. | (+1) Unmeasured confounding would not suffice to explain away the effect estimate. | (0)  Several potential confounders that would shift the RR in both directions. | (+1) Significant positive association detected in the main analysis. | ⊗⊗⊗〇 Moderate |
| Long-term_NO_2__Cardiovascular mortality | (0) No differences between studies with low/moderate versus high RoB | (0) The research question in the studies reflects the original question. | (0) 80% prediction interval included unity, but is not twice the confidence interval. | (-1) Number of mortality cases lower than 940,000. | (-1) Publication bias was not assessed. | (0) Unmeasured confounding could influence the effect estimate. | (0)  Several potential confounders that would shift the RR in both directions. | (0) No significant association detected in the main analysis. | ⊗〇〇〇 Very low |
| Long-term_NO_2__Respiratory mortality | (0) No differences between studies with low/moderate versus high RoB | (0) The research question in the studies reflects the original question. | (0) 80% prediction interval included unity, but is not twice the confidence interval. | (-1) Number of mortality cases lower than 940,000. | (-1) Publication bias was not assessed. | (0) Unmeasured confounding could influence the effect estimate. | (0)  Several potential confounders that would shift the RR in both directions. | (0) No significant association detected in the main analysis. | ⊗〇〇〇 Very low |
| Long-term_O_3__All-cause mortality | (0) No differences between studies with low/moderate versus high RoB | (0) The research question in the studies reflects the original question. | (0) 80% prediction interval did not include unity. | (-1) Number of mortality cases lower than 940,000. | (-1) Publication bias was not assessed. | (0) Unmeasured confounding could influence the effect estimate. | (0)  Several potential confounders that would shift the RR in both directions. | (+1) Significant positive association detected in the main analysis. | ⊗⊗〇〇 Low |
| Long-term_O_3__Cardiovascular mortality | (0) No differences between studies with low/moderate versus high RoB | (0) The research question in the studies reflects the original question. | (0) 80% prediction interval did not include unity. | (-1) Number of mortality cases lower than 940,000. | (-1) Publication bias was not assessed. | (+1) Unmeasured confounding would not suffice to explain away the effect estimate. | (0)  Several potential confounders that would shift the RR in both directions. | (+1) Significant positive association detected in the main analysis. | ⊗⊗⊗〇 Moderate |
| Short-term_PM_2.5__All-cause mortality | (0) No differences between studies with low/moderate versus high RoB | (0) The research question in the studies reflects the original question. | (0) 80% prediction interval did not include unity. | (0) Number of mortality cases higher than 100,000. | (0) Publication bias was not detected. | (+1) Unmeasured confounding would not suffice to explain away the effect estimate. | (0)  Several potential confounders that would shift the RR in both directions. | (+1) Significant positive association detected in the main analysis. | ⊗⊗⊗⊗ High |
| Short-term_PM_2.5__Cardiovascular mortality | (0) No differences between studies with low/moderate versus high RoB | (0) The research question in the studies reflects the original question. | (0) 80% prediction interval included unity, but is not twice the confidence interval. | (0) Number of mortality cases higher than 100,000. | (0) Publication bias detected, but no difference between multicity and single city studies was observed. | (+1) Unmeasured confounding would not suffice to explain away the effect estimate. | (0)  Several potential confounders that would shift the RR in both directions. | (+1) Significant positive association detected in the main analysis. | ⊗⊗⊗⊗ High |
| Short-term_PM_2.5__Respiratory mortality | (0) No differences between studies with low/moderate versus high RoB | (0) The research question in the studies reflects the original question. | (0) 80% prediction interval did not include unity. | (0) Number of mortality cases higher than 100,000. | (0) Publication bias detected, but no difference between multicity and single city studies was observed. | (+1) Unmeasured confounding would not suffice to explain away the effect estimate. | (0)  Several potential confounders that would shift the RR in both directions. | (+1) Significant positive association detected in the main analysis. | ⊗⊗⊗⊗ High |
| Short-term_NO_2__All-cause mortality | (0) No differences between studies with low/moderate versus high RoB | (0) The research question in the studies reflects the original question. | (0) 80% prediction interval did not include unity. | (0) Number of mortality cases higher than 100,000. | (0) Publication bias detected, but no difference between multicity and single city studies was observed. | (+1) Unmeasured confounding would not suffice to explain away the effect estimate. | (0)  Several potential confounders that would shift the RR in both directions. | (+1) Significant positive association detected in the main analysis. | ⊗⊗⊗⊗ High |
| Short-term_NO_2__Cardiovascular mortality | (0) No differences between studies with low/moderate versus high RoB | (0) The research question in the studies reflects the original question. | (0) 80% prediction interval did not include unity. | (0) Number of mortality cases higher than 100,000. | (0) Publication bias detected, but no difference between multicity and single city studies was observed. | (+1) Unmeasured confounding would not suffice to explain away the effect estimate. | (0)  Several potential confounders that would shift the RR in both directions. | (+1) Significant positive association detected in the main analysis. | ⊗⊗⊗⊗ High |
| Short-term_NO_2__Respiratory mortality | (0) No differences between studies with low/moderate versus high RoB | (0) The research question in the studies reflects the original question. | (0) 80% prediction interval did not include unity. | (0) Number of mortality cases higher than 100,000. | (0) Publication bias detected, but no difference between multicity and single city studies was observed. | (+1) Unmeasured confounding would not suffice to explain away the effect estimate. | (0)  Several potential confounders that would shift the RR in both directions. | (+1) Significant positive association detected in the main analysis. | ⊗⊗⊗⊗ High |
| Short-term_O_3__All-cause mortality | (0) No differences between studies with low/moderate versus high RoB | (0) The research question in the studies reflects the original question. | (0) 80% prediction interval did not include unity. | (0) Number of mortality cases higher than 100,000. | (0) Publication bias detected, but no difference between multicity and single city studies was observed. | (+1) Unmeasured confounding would not suffice to explain away the effect estimate. | (0)  Several potential confounders that would shift the RR in both directions. | (+1) Significant positive association detected in the main analysis. | ⊗⊗⊗⊗ High |
| Short-term_O_3__Cardiovascular mortality | (0) No differences between studies with low/moderate versus high RoB | (0) The research question in the studies reflects the original question. | (0) 80% prediction interval included unity, but is not twice the confidence interval. | (0) Number of mortality cases higher than 100,000. | (0) Publication bias detected, but no difference between multicity and single city studies was observed. | (0) Unmeasured confounding could influence the effect estimate. | (0)  Several potential confounders that would shift the RR in both directions. | (+1) Significant positive association detected in the main analysis. | ⊗⊗⊗⊗ High |
| Short-term_O_3__Respiratory mortality | (0) No differences between studies with low/moderate versus high RoB | (0) The research question in the studies reflects the original question. | (0) 80% prediction interval included unity, but is not twice the confidence interval. | (0) Number of mortality cases higher than 100,000. | (0) Publication bias detected, but no difference between multicity and single city studies was observed. | (0) Unmeasured confounding could influence the effect estimate. | (0)  Several potential confounders that would shift the RR in both directions. | (+1) Significant positive association detected in the main analysis. | ⊗⊗⊗⊗ High |

References:

2020. Approach to assessing the certainty of evidence from systematic reviews informing WHO global air quality guidelines. WHO Global Air Quality Guidelines Working Group on Certainty of Evidence Assessment.

Ban, J., Wang, Q., Ma, R., Zhang, Y., Shi, W., Zhang, Y., Chen, C., Sun, Q., Wang, Y., Guo, X., Li, T., 2021. Associations between short-term exposure to PM2.5 and stroke incidence and mortality in China: A case-crossover study and estimation of the burden. Environmental Pollution 268, 115743.

Cai, J., Peng, C., Yu, S., Pei, Y., Liu, N., Wu, Y., Fu, Y., Cheng, J., 2019. Association between PM2. 5 exposure and all-cause, non-accidental, accidental, different respiratory diseases, sex and age mortality in Shenzhen, China. International Journal of Environmental Research and Public Health 16, 401.

Cai, J., Yu, S., Pei, Y., Peng, C., Liao, Y., Liu, N., Ji, J., Cheng, J., 2018. Association between airborne fine particulate matter and residents’ cardiovascular diseases, ischemic heart disease and cerebral vascular disease mortality in areas with lighter air pollution in China. International Journal of Environmental Research and Public Health 15, 1918.

Cao, J., Xu, H., Xu, Q., Chen, B., Kan, H., 2012. Fine particulate matter constituents and cardiopulmonary mortality in a heavily polluted Chinese city. Environmental health perspectives 120, 373-378.

Cao, J., Yang, C., Li, J., Chen, R., Chen, B., Gu, D., Kan, H., 2011. Association between long-term exposure to outdoor air pollution and mortality in China: A cohort study. Journal of Hazardous Materials 186, 1594-1600.

Cao, R., Cui, L., Jiang, C., Jing, Y., Zhou, L., Zhang, L., Liu, S., 2018. Association between ambient ozone and mortality of respiratory diseases in Jinan,China: a time series analysis. Journal of Shandong University (Health Sciences) 56, 91-97.

Chen, C., Li, T., Sun, Q., Shi, W., He, M.Z., Wang, J., Liu, J., Zhang, M., Jiang, Q., Wang, M., Shi, X., 2023a. Short-term exposure to ozone and cause-specific mortality risks and thresholds in China: Evidence from nationally representative data, 2013-2018. Environment International 171, 107666.

Chen, C., Li, T., Wang, L., Qi, J., Shi, W., He, M.Z., Sun, Q., Wang, J., Zhu, H., Shi, X., 2019a. Short-term exposure to fine particles and risk of cause-specific mortality—China, 2013-2018. China CDC Weekly 1, 8.

Chen, C., Zhu, P., Lan, L., Zhou, L., Liu, R., Sun, Q., Ban, J., Wang, W., Xu, D., Li, T., 2018a. Short-term exposures to PM2.5 and cause-specific mortality of cardiovascular health in China. ENVIRONMENTAL RESEARCH 161, 188-194.

Chen, G., Song, G., Jiang, L., Zhang, Y., Zhao, N., Chen, B., Kan, H., 2008. Short-term effects of ambient gaseous pollutants and particulate matter on daily mortality in Shanghai, China. J Occup Health 50, 41-47.

Chen, G., Wang, A., Li, S., Zhao, X., Wang, Y., Li, H., Meng, X., Knibbs, L.D., Bell, M.L., Abramson, M.J., 2019b. Long-term exposure to air pollution and survival after ischemic stroke: the China national stroke registry cohort. STROKE 50, 563-570.

Chen, H., Li, X., Su, X., Han, R., Dong, L., 2022a. Time-series analysis on health effects of atmospheric ozone exposure on non-accidental deaths in Kunming during 2017—2019. Journal of Environmental and Occupational Medicine 39, 883-889.

Chen, J., Shi, C., Li, Y., Ni, H., Zeng, J., Lu, R., Zhang, L., 2021a. Effects of short-term exposure to ambient airborne pollutants on COPD-related mortality among the elderly residents of Chengdu city in Southwest China. Environmental health and preventive medicine 26, 1-10.

Chen, J., Zeng, J., Shi, C., Liu, R., Lu, R., Mao, S., Zhang, L., 2019c. Associations between short-term exposure to gaseous pollutants and pulmonary heart disease-related mortality among elderly people in Chengdu, China. ENVIRONMENTAL HEALTH 18, 1-10.

Chen, J., Zhong, W., Lin, X., Lin, L., 2020. A time-series analysis on association between O3 concentration and the mortality of chronic respiratory diseases in Sanming City. Journal of Public Health and Preventive Medicine 30, 57-60.

Chen, K., Zhou, L., Chen, X., Bi, J., Kinney, P.L., 2017a. Acute effect of ozone exposure on daily mortality in seven cities of Jiangsu Province, China: No clear evidence for threshold. ENVIRONMENTAL RESEARCH 155, 235-241.

Chen, Q., Chen, Q., Wang, Q., Xu, R., Liu, T., Liu, Y., Ding, Z., Sun, H., 2022b. Particulate matter and ozone might trigger deaths from chronic ischemic heart disease. Ecotoxicology and Environmental Safety 242, 113931.

Chen, Q., Wang, Q., Xu, B., Xu, Y., Ding, Z., Sun, H., 2021b. Air pollution and cardiovascular mortality in Nanjing, China: Evidence highlighting the roles of cumulative exposure and mortality displacement. Chemosphere 265, 129035.

Chen, Q., Wang, Q., Xu, B., Xu, Y., Ding, Z., Zhou, J., Sun, H., 2022c. Cumulative effects of ambient particulate matter pollution on deaths: A multicity analysis of mortality displacement. Chemosphere 286, 131615.

Chen, R., Pan, G., Kan, H., Tan, J., Song, W., Wu, Z., Xu, X., Xu, Q., Jiang, C., Chen, B., 2010. Ambient air pollution and daily mortality in Anshan, China: A time-stratified case-crossover analysis. Science of The Total Environment 408, 6086-6091.

Chen, R., Samoli, E., Wong, C.-M., Huang, W., Wang, Z., Chen, B., Kan, H., Group, C.C., 2012. Associations between short-term exposure to nitrogen dioxide and mortality in 17 Chinese cities: the China Air Pollution and Health Effects Study (CAPES). Environment International 45, 32-38.

Chen, R., Yin, P., Meng, X., Liu, C., Wang, L., Xu, X., Ross, J.A., Tse, L.A., Zhao, Z., Kan, H., 2017b. Fine particulate air pollution and daily mortality. A nationwide analysis in 272 Chinese cities. American Journal of Respiratory and Critical Care Medicine 196, 73-81.

Chen, R., Yin, P., Meng, X., Wang, L., Liu, C., Niu, Y., Lin, Z., Liu, Y., Liu, J., Qi, J., 2018b. Associations between ambient nitrogen dioxide and daily cause-specific mortality: evidence from 272 Chinese cities. Epidemiology 29, 482-489.

Chen, R., Zhang, Y., Yang, C., Zhao, Z., Xu, X., Kan, H., 2013. Acute effect of ambient air pollution on stroke mortality in the China air pollution and health effects study. STROKE 44, 954-960.

Chen, S., Lin, X., Du, Z., Zhang, Y., Zheng, L., Ju, X., Guo, T., Wang, X., Chen, L., Jiang, J., Hu, W., Zhang, W., Hao, Y., 2023b. Potential causal links between long-term ambient particulate matter exposure and cerebrovascular mortality: Insights from a large cohort in southern China. Environmental Pollution 328, 121336.

CHEN, X., WANG, L.-c., GUO, Q.-l., HU, H., LU, Y., ZHU, X.-h., LI, H.-m., 2019d. Association between PM2. 5 pollution in ambient air and cardio-cerebrovascular mortality in Suzhou in 2016: a time series analysis. Chinese Journal of Disease Control & Prevention, 661-666.

Chen, Y., Chen, R., Chen, Y., Dong, X., Zhu, J., Liu, C., van Donkelaar, A., Martin, R.V., Li, H., Kan, H., Jiang, Q., Fu, C., 2021c. The prospective effects of long-term exposure to ambient PM2.5 and constituents on mortality in rural East China. Chemosphere 280, 130740.

Chen, Y., Jiao, Z., Chen, P., Fan, L., Zhou, X., Pu, Y., Du, W., Yin, L., 2021d. Short-term effect of fine particulate matter and ozone on non-accidental mortality and respiratory mortality in Lishui district, China. BMC PUBLIC HEALTH 21, 1-11.

Chen, Z., Chen, X., Dong, H., Wang, K., 2019e. Effect of fine particulate matter on daily mortality in the aged people in Changzhou City: a time-series study. Modern Preventive Medicine 46, 1181-1185.

Chen, Z., Cui, X., Wang, B., Hu, Y., Dai, L., Cao, X., Wang, C., Shi, T., 2022d. A time series study on the effect of low air pollution level of NO2 on the death of cardiovascular and cerebrovascular diseases in residents. Journal of Public Health and Preventive Medicine, 27-31.

Chen, Z., Fu, Q., Mao, G., Wu, L., Xu, P., Xu, D., Wang, Z., Pan, X., Chen, Y., Lou, X., 2021e. Increasing mortality caused by chronic obstructive pulmonary disease (COPD) in relation with exposure to ambient fine particulate matters: an analysis in Southeastern China. Environmental Science and Pollution Research 28, 53605-53613.

Cheng, H., Zhu, F., Lei, R., Shen, C., Liu, J., Yang, M., Ding, R., Cao, J., 2019. Associations of ambient PM2.5 and O3 with cardiovascular mortality: a time-series study in Hefei, China. International Journal of Biometeorology 63, 1437-1447.

Dai, J., Chen, R., Meng, X., Yang, C., Zhao, Z., Kan, H., 2015. Ambient air pollution, temperature and out-of-hospital coronary deaths in Shanghai, China. Environmental Pollution 203, 116-121.

Deng, J., Hu, X., Xiao, C., Pan, F., 2021. The association between gaseous pollutants and non-accidental mortality: a time series study. Environmental Geochemistry and Health, 1-11.

Dong, G.-H., Zhang, P., Sun, B., Zhang, L., Chen, X., Ma, N., Yu, F., Guo, H., Huang, H., Lee, Y.L., 2012. Long-term exposure to ambient air pollution and respiratory disease mortality in Shenyang, China: a 12-year population-based retrospective cohort study. Respiration 84, 360-368.

Dong, H., Yu, Y., Yao, S., Lu, Y., Chen, Z., Li, G., Yao, Y., Yao, X., Wang, S.-L., Zhang, Z., 2018. Acute effects of air pollution on ischaemic stroke onset and deaths: a time-series study in Changzhou, China. BMJ OPEN 8.

Dong, Z., Wang, H., Yin, P., Wang, L., Chen, R., Fan, W., Xu, Y., Zhou, M., 2020. Time-weighted average of fine particulate matter exposure and cause-specific mortality in China: a nationwide analysis. The Lancet Planetary Health 4, e343-e351.

Duan, Y., Liao, Y., Li, H., Yan, S., Zhao, Z., Yu, S., Fu, Y., Wang, Z., Yin, P., Cheng, J., 2019. Effect of changes in season and temperature on cardiovascular mortality associated with nitrogen dioxide air pollution in Shenzhen, China. Science of The Total Environment 697, 134051.

Fan, Z.Y., Xia, M.Y., Ping, L.H., Wen, C.B., Liu, F.F., Ran, J.H., 2023. Short-term exposure to ozone and nonaccidental mortality in Northeast China. Biomedical and Environmental Sciences 36, 99-102.

Fang, B., Qian, N., Chen, L., Qiao, J., Jin, S., Cai, R., Wang, C., 2022. Risk assessment of excess mortality from respiratory diseases among the population caused by short-term exposure to PM2.5 in Shanghai from 2013 to 2017. Disease Surveillance 37, 1112-1117.

Fang, X., Fang, B., Wang, C., Xia, T., Bottai, M., Fang, F., Cao, Y., 2017. Relationship between fine particulate matter, weather condition and daily non-accidental mortality in Shanghai, China: A Bayesian approach. PLOS ONE 12, e0187933.

Fang, X., Fang, B., Wang, C., Xia, T., Bottai, M., Fang, F., Cao, Y., 2019. Comparison of frequentist and Bayesian generalized additive models for assessing the association between daily exposure to fine particles and respiratory mortality: A simulation study. International Journal of Environmental Research and Public Health 16, 746.

Feng, Q., Chen, Y., Su, S., Zhang, X., Lin, X., 2022. Acute effect of fine particulate matter and respiratory mortality in Changsha, China: a time-series analysis. BMC Pulmonary Medicine 22, 1-11.

Gao, P., Wu, Y., He, L., Wang, L., Fu, Y., Chen, J., Zhang, F., Krafft, T., Martens, P., 2023. Adverse short-term effects of ozone on cardiovascular mortalities modified by season and temperature: a time-series study. FRONTIERS IN PUBLIC HEALTH 11, 1182337.

Ge, E., Gao, J., Wei, X., Ren, Z., Wei, J., Liu, X., Wang, X., Zhong, J., Lu, J., Tian, X., 2022. Effect modification of greenness on PM2. 5 associated all-cause mortality in a multidrug-resistant tuberculosis cohort. Thorax 77, 1202-1209.

Ge, X., Wang, L., Lu, Y., Yao, Y., Hu, Y., Liu, F., Yang, H., 2015. Relationship between ambient particulate matter and daily mortality of respiratory disease: A time-series analysis. J. Environ. Health 32, 494-497.

Gong, T., Sun, Z., Zhang, X., Wang, S., 2019a. Natural and social factor as modifiers of the effects of PM2. 5 on daily cardiovascular mortality in Beijing, China. China Environ Sci 39, 1289-1298.

Gong, T., Sun, Z., Zhang, X., Zhang, Y., Wang, S., Han, L., Zhao, D., Ding, D., Zheng, C., 2019b. Associations of black carbon and PM2.5 with daily cardiovascular mortality in Beijing, China. Atmospheric Environment 214, 116876.

Gu, Y., Chen, R., 2017. A case-crossover study on particulate matter air pollution and residents' cardio-cerebrovascular mortality in Minhang District, Shanghai. Journal of Environmental and Occupational Medicine 34, 220-223.

Guan, M., Sun, C., Tang, D., Kang, H., Chen, F., 2022. A Time-Series Analysis on the Association Between Fine Particulate Matter and Daily Mortality—Shijiazhuang City, Hebei Province, China, 2015–2020. China CDC Weekly 4, 226.

Guo, B., Chen, F., Deng, Y., Zhang, H., Qiao, X., Qiao, Z., Ji, K., Zeng, J., Luo, B., Zhang, W., 2018. Using rush hour and daytime exposure indicators to estimate the short-term mortality effects of air pollution: A case study in the Sichuan Basin, China. Environmental Pollution 242, 1291-1298.

Guo, C., Yu, T., Lin, C., Chang, L.-Y., Bo, Y., Wong, M.C., Tam, T., Lau, A.K., Lao, X.Q., 2022. Habitual exercise, air pollution, and pneumonia mortality: a longitudinal cohort study of approximately 0.4 million adults. American Journal of Epidemiology 191, 1732-1741.

Guo, Y., Barnett, A.G., Zhang, Y., Tong, S., Yu, W., Pan, X., 2010. The short-term effect of air pollution on cardiovascular mortality in Tianjin, China: comparison of time series and case-crossover analyses. Sci Total Environ 409, 300-306.

Guo, Y., Ma, Y., Zhang, Y., Huang, S., Wu, Y., Yu, S., Zou, F., Cheng, J., 2017. Time series analysis of ambient air pollution effects on daily mortality. Environmental Science and Pollution Research 24, 20261-20272.

He, C., Liu, C., Chen, R., Meng, X., Wang, W., Ji, J., Kang, L., Liang, J., Li, X., Liu, Y., Yu, X., Zhu, J., Wang, Y., Kan, H., 2022. Fine particulate matter air pollution and under-5 children mortality in China: A national time-stratified case-crossover study. Environment International 159, 107022.

He, M.Z., Kinney, P.L., Li, T., Chen, C., Sun, Q., Ban, J., Wang, J., Liu, S., Goldsmith, J., Kioumourtzoglou, M.-A., 2020. Short-and intermediate-term exposure to NO2 and mortality: a multi-county analysis in China. Environmental Pollution 261, 114165.

He, Z.H., Shasha, 2022. Time series analysis of effects of ozone exposure on human mortality in Nantong,China,2017 to 2020. Journal of Environmental Hygiene 12, 358-362.

Hong, W., Jun-hong, G., Le, F., Zhi-yong, L., Jin-ren, L., Xiao-lin, F., Cun-zhi, L., Qing, L., Qing-bo, K., Guo-dong, Z., 2020. Effects of short-term exposure to atmospheric PM2.5 on deaths due to cardiovascular and cerebrovascular diseases in Xi'an. Journal of Environmental and Occupational Medicine 37, 975-980.

Hu, J., Yu, L., Yang, Z., Qiu, J., Li, J., Shen, P., Lin, H., Shui, L., Tang, M., Jin, M., 2023. Long-Term Exposure to PM2. 5 and Mortality: A Cohort Study in China. Toxics 11, 727.

Hu, Y., Liu, C., Chen, R., Kan, H., Zhou, M., Zhao, B., 2021. Associations between total mortality and personal exposure to outdoor-originated NO2 in 271 Chinese cities. Atmospheric Environment 246, 118170.

Huang, S., Zhong, W., Lin, L., Lin, X., Yin, Y., 2020. Relationship between the elderly death counts and atmospheric fine particulates in Fuzhou City by time series study. Journal of Strait Preventive Medicine 26, 11-13.

Huang, W., Cao, J., Tao, Y., Dai, L., Lu, S.-E., Hou, B., Wang, Z., Zhu, T., 2012. Seasonal variation of chemical species associated with short-term mortality effects of PM_2.5_ in Xi’an, a central city in China. American Journal of Epidemiology 175, 556-566.

Huang, W., Zhou, Y., Chen, X., Zeng, X., Knibbs, L.D., Zhang, Y., Jalaludin, B., Dharmage, S.C., Morawska, L., Guo, Y., 2023. Individual and joint associations of long-term exposure to air pollutants and cardiopulmonary mortality: a 22-year cohort study in Northern China. The Lancet Regional Health–Western Pacific.

Ji, A., Zhang, C., Wu, Q., Cui, L., Quan, Z., Li, C., 2018. Effects of PM25 on death of respiratory diseases in population in Yanbian Korean Autonomous Prefecture : a case-crossover study. Occupation and Health 34, 2815-2818.

Ji, J.S., Liu, L., Zhang, J., Kan, H., Zhao, B., Burkart, K.G., Zeng, Y., 2022. NO2 and PM2.5 air pollution co-exposure and temperature effect modification on pre-mature mortality in advanced age: a longitudinal cohort study in China. ENVIRONMENTAL HEALTH 21, 97.

Ji, J.S., Zhu, A., Lv, Y., Shi, X., 2020a. Interaction between residential greenness and air pollution mortality: analysis of the Chinese Longitudinal Healthy Longevity Survey. The Lancet Planetary Health 4, e107-e115.

Ji, S., Zhou, Q., Jiang, Y., He, C., Chen, Y., Wu, C., Liu, B., 2020b. The interactive effects between particulate matter and heat waves on circulatory mortality in Fuzhou, China. International Journal of Environmental Research and Public Health 17, 5979.

Jiang, S., Li, W., Bu, L., Yang, Y., Feng, W., Lv, J., 2022. Time series analysis of the relationship between atmospheric PM25 and the death of residents from respiratory diseases in Guangzhou. South China Journal of Preventive Medicine 48, 721-724.

Jie, G., Mingyue, M., Chunling, X., Zhang, C., Jianping, C., Hong, L., Yiming, D., Min, L., 2018. Association of air pollution and mortality of acute lower respiratory tract infections in Shenyang, China: A time series analysis study. Iranian journal of public health 47, 1261.

Kan, H.-D., Chen, B.-H., Jia, J., 2003a. A case-crossover study of ambient air pollution and daily mortality in Shanghai. Zhonghua liu Xing Bing xue za zhi= Zhonghua Liuxingbingxue Zazhi 24, 863-867.

Kan, H., Chen, B., 2003. A case-crossover analysis of air pollution and daily mortality in Shanghai. Journal of occupational health 45, 119-124.

Kan, H., Chen, B., Zhao, N., London, S.J., Song, G., Chen, G., Zhang, Y., Jiang, L., 2010. Part 1. A time-series study of ambient air pollution and daily mortality in Shanghai, China. Research report (Health Effects Institute), 17-78.

Kan, H., Jia, J., Chen, B., 2003b. Acute stroke mortality and air pollution: new evidence from Shanghai, China. Journal of occupational health 45, 321-323.

Kan, H., Jia, J., Chen, B., 2004. A time series study on the relationship between stroke deaths and air pollution among residents in a certain district of Shanghai. Journal of Hygiene Research, 36-38.

Kan, H., London, S.J., Chen, G., Zhang, Y., Song, G., Zhao, N., Jiang, L., Chen, B., 2007. Differentiating the effects of fine and coarse particles on daily mortality in Shanghai, China. Environment International 33, 376-384.

Kan, H., London, S.J., Chen, G., Zhang, Y., Song, G., Zhao, N., Jiang, L., Chen, B., 2008. Season, sex, age, and education as modifiers of the effects of outdoor air pollution on daily mortality in Shanghai, China: The Public Health and Air Pollution in Asia (PAPA) Study. Environ Health Perspect 116, 1183-1188.

Lee, B.J., Kim, B., Lee, K., 2014. Air pollution exposure and cardiovascular disease. Toxicol Res 30, 71-75.

Leepe, K.A., Li, M., Fang, X., Hiyoshi, A., Cao, Y., 2019. Acute effect of daily fine particulate matter pollution on cerebrovascular mortality in Shanghai, China: a population-based time series study. Environmental Science and Pollution Research 26, 25491-25499.

Lei, R., Zhu, F., Cheng, H., Liu, J., Shen, C., Zhang, C., Xu, Y., Xiao, C., Li, X., Zhang, J., Ding, R., Cao, J., 2019. Short-term effect of PM2.5/O3 on non-accidental and respiratory deaths in highly polluted area of China. Atmospheric Pollution Research 10, 1412-1419.

Li, D., Wang, J.-b., Zhang, Z.-y., Shen, P., Zheng, P.-w., Jin, M.-j., Lu, H.-c., Lin, H.-b., Chen, K., 2018a. Association between short-term exposure to ambient air pollution and daily mortality: a time-series study in Eastern China. Environmental Science and Pollution Research 25, 16135-16143.

Li, M., Dong, H., Wang, B., Zhao, W., Sakhvidi, M.J.Z., Li, L., Lin, G., Yang, J., 2021. Association between ambient ozone pollution and mortality from a spectrum of causes in Guangzhou, China. Science of The Total Environment 754, 142110.

Li, N., Peng, X., 2010. A time-series study between air pollutant and the respiratory daily mortality in Guangzhou. Acta Medicinae Universitatis Scientiae et Technologiae Huazhong 39, 863-867.

Li, P., Xin, J., Wang, Y., Li, G., Pan, X., Wang, S., Cheng, M., Wen, T., Wang, G., Liu, Z., 2015a. Association between particulate matter and its chemical constituents of urban air pollution and daily mortality or morbidity in Beijing City. Environmental Science and Pollution Research 22, 358-368.

Li, P., Xin, J., Wang, Y., Wang, S., Li, G., Pan, X., Liu, Z., Wang, L., 2013a. The acute effects of fine particles on respiratory mortality and morbidity in Beijing, 2004–2009. Environmental Science and Pollution Research 20, 6433-6444.

Li, P., Xin, J., Wang, Y., Wang, S., Shang, K., Liu, Z., Li, G., Pan, X., Wei, L., Wang, M., 2013b. Time-series analysis of mortality effects from airborne particulate matter size fractions in Beijing. Atmospheric Environment 81, 253-262.

Li, T., Yan, M., Ma, W., Ban, J., Liu, T., Lin, H., Liu, Z., 2015b. Short-term effects of multiple ozone metrics on daily mortality in a megacity of China. Environmental Science and Pollution Research 22, 8738-8746.

Li, T., Yan, M., Sun, Q., Anderson, G.B., 2018b. Mortality risks from a spectrum of causes associated with wide-ranging exposure to fine particulate matter: A case-crossover study in Beijing, China. Environment International 111, 52-59.

Li, T., Zhang, Y., Wang, J., Xu, D., Yin, Z., Chen, H., Lv, Y., Luo, J., Zeng, Y., Liu, Y., Kinney, P.L., Shi, X., 2018c. All-cause mortality risk associated with long-term exposure to ambient PM_2.5_ in China: a cohort study. The Lancet Public Health 3, e470-e477.

Li, W., Cao, Y., Li, R., Ma, X., Chen, J., Wu, Z., Xu, Q., 2018d. The spatial variation in the effects of air pollution on cardiovascular mortality in Beijing, China. Journal of Exposure Science & Environmental Epidemiology 28, 297-304.

Li, W., Pei, L., Li, A., Luo, K., Cao, Y., Li, R., Xu, Q., 2019a. Spatial variation in the effects of air pollution on cardiovascular mortality in Beijing, China. Environmental Science and Pollution Research 26, 2501-2511.

Li, Y., Ma, Z., Zheng, C., Shang, Y., 2015c. Ambient temperature enhanced acute cardiovascular-respiratory mortality effects of PM2.5 in Beijing, China. International Journal of Biometeorology 59, 1761-1770.

Li, Y., Zheng, C., Ma, Z., Quan, W., 2019b. Acute and cumulative effects of haze fine particles on mortality and the seasonal characteristics in Beijing, China, 2005–2013: A time-stratified case-crossover study. International Journal of Environmental Research and Public Health 16, 2383.

Liang, F., Liu, F., Huang, K., Yang, X., Li, J., Xiao, Q., Chen, J., Liu, X., Cao, J., Shen, C., Yu, L., Lu, F., Wu, X., Wu, X., Li, Y., Hu, D., Huang, J., Liu, Y., Lu, X., Gu, D., 2020. Long-Term Exposure to Fine Particulate Matter and Cardiovascular Disease in China. Journal of the American College of Cardiology 75, 707-717.

Liang, F., Xiao, Q., Gu, D., Xu, M., Tian, L., Guo, Q., Wu, Z., Pan, X., Liu, Y., 2018a. Satellite-based short-and long-term exposure to PM2. 5 and adult mortality in urban Beijing, China. Environmental Pollution 242, 492-499.

Liang, H., Qiu, H., Tian, L., 2018b. Short-term effects of fine particulate matter on acute myocardial infraction mortality and years of life lost: A time series study in Hong Kong. Sci Total Environ 615, 558-563.

Liang, R., Chen, R., Yin, P., van Donkelaar, A., Martin, R.V., Burnett, R., Cohen, A.J., Brauer, M., Liu, C., Wang, W., Lei, J., Wang, L., Wang, L., Zhang, M., Kan, H., Zhou, M., 2022. Associations of long-term exposure to fine particulate matter and its constituents with cardiovascular mortality: A prospective cohort study in China. Environment International 162, 107156.

Liang, R., Yin, P., Wang, L., Li, Y., Liu, J., Liu, Y., You, J., Qi, J., Zhou, M., 2017. Acute effect of fine particulate matters on daily cardiovascular disease mortality in seven cities of China. Zhonghua liu Xing Bing xue za zhi= Zhonghua Liuxingbingxue Zazhi 38, 283-289.

Lin, C., Ma, Y., Liu, R., Shao, Y., Ma, Z., Zhou, L., Jing, Y., Bell, M.L., Chen, K., 2022. Associations between short-term ambient ozone exposure and cause-specific mortality in rural and urban areas of Jiangsu, China. ENVIRONMENTAL RESEARCH 211, 113098.

Lin, H., Liu, T., Xiao, J., Zeng, W., Li, X., Guo, L., Zhang, Y., Xu, Y., Tao, J., Xian, H., 2016a. Mortality burden of ambient fine particulate air pollution in six Chinese cities: results from the Pearl River Delta study. Environment International 96, 91-97.

Lin, H., Ma, W., Qiu, H., Vaughn, M.G., Nelson, E.J., Qian, Z., Tian, L., 2016b. Is standard deviation of daily PM2.5 concentration associated with respiratory mortality? Environmental Pollution 216, 208-214.

Lin, H., Ratnapradipa, K., Wang, X., Zhang, Y., Xu, Y., Yao, Z., Dong, G., Liu, T., Clark, J., Dick, R., 2017. Hourly peak concentration measuring the PM2.5-mortality association: Results from six cities in the Pearl River Delta study. Atmospheric Environment 161, 27-33.

Lin, H., Tao, J., Du, Y., Liu, T., Qian, Z., Tian, L., Di, Q., Rutherford, S., Guo, L., Zeng, W., Xiao, J., Li, X., He, Z., Xu, Y., Ma, W., 2016c. Particle size and chemical constituents of ambient particulate pollution associated with cardiovascular mortality in Guangzhou, China. Environmental Pollution 208, 758-766.

Lin, H., Tao, J., Du, Y., Liu, T., Qian, Z., Tian, L., Di, Q., Zeng, W., Xiao, J., Guo, L., Li, X., Xu, Y., Ma, W., 2016d. Differentiating the effects of characteristics of PM pollution on mortality from ischemic and hemorrhagic strokes. International Journal of Hygiene and Environmental Health 219, 204-211.

Liu, C., Chen, R., Sera, F., Vicedo-Cabrera, A.M., Guo, Y., Tong, S., Coelho, M.S., Saldiva, P.H., Lavigne, E., Matus, P., 2019a. Ambient particulate air pollution and daily mortality in 652 cities. New England Journal of Medicine 381, 705-715.

Liu, G., Sun, B., Yu, L., Chen, J., Han, B., Liu, B., Chen, J., 2019b. Short-term exposure to ambient air pollution and daily atherosclerotic heart disease mortality in a cool climate. Environmental Science and Pollution Research 26, 23603-23614.

Liu, H., Meng, Z.H., Shang, Y., Lv, Z.F., Jin, X.X., Fu, M.L., He, K.B., 2018. Shipping emission forecasts and cost-benefit analysis of China ports and key regions' control. Environ Pollut 236, 49-59.

Liu, J., Ruan, Y., Wu, Q., Ma, Y., He, X., Li, L., Li, S., Niu, J., Luo, B., 2020. Has the mortality risk declined after the improvement of air quality in an ex-heavily polluted Chinese city-Lanzhou? Chemosphere 242, 125196.

Liu, L., Luo, S., Zhang, Y., Yang, Z., Zhou, P., Mo, S., Zhang, Y., 2022a. Longitudinal impacts of PM2.5 constituents on adult mortality in China. Environmental Science & Technology 56, 7224-7233.

Liu, M., Xue, X., Zhou, B., Zhang, Y., Sun, B., Chen, J., Li, X., 2019c. Population susceptibility differences and effects of air pollution on cardiovascular mortality: epidemiological evidence from a time-series study. Environmental Science and Pollution Research 26, 15943-15952.

Liu, S., Zhang, Y., Ma, R., Liu, X., Liang, J., Lin, H., Shen, P., Zhang, J., Lu, P., Tang, X., Li, T., Gao, P., 2022b. Long-term exposure to ozone and cardiovascular mortality in a large Chinese cohort. Environment International 165, 107280.

Liu, T., Gong, W., Zhou, C., Bai, G., Meng, R., Huang, B., Zhang, H., Xu, Y., Hu, R., Hou, Z., 2023a. Mortality burden based on the associations of ambient PM2. 5 with cause-specific mortality in China: Evidence from a death-spectrum wide association study (DWAS). Ecotoxicology and Environmental Safety 259, 115045.

Liu, T., Gong, W., Zhou, C., Bai, G., Meng, R., Huang, B., Zhang, H., Xu, Y., Hu, R., Hou, Z., Xiao, Y., Li, J., Xu, X., Jin, D., Qin, M., Zhao, Q., Xu, Y., Hu, J., Xiao, J., He, G., Rong, Z., Zeng, F., Yang, P., Liu, D., Yuan, L., Cao, G., Chen, Z., Yu, S., Yang, S., Huang, C., Du, Y., Yu, M., Lin, L., Liang, X., Ma, W., 2023b. Mortality burden based on the associations of ambient PM2.5 with cause-specific mortality in China: Evidence from a death-spectrum wide association study (DWAS). Ecotoxicology and Environmental Safety 259, 115045.

Liu, T., Li, T.T., Zhang, Y.H., Xu, Y.J., Lao, X.Q., Rutherford, S., Chu, C., Luo, Y., Zhu, Q., Xu, X.J., Xie, H.Y., Liu, Z.R., Ma, W.J., 2013. The short-term effect of ambient ozone on mortality is modified by temperature in Guangzhou, China. Atmospheric Environment 76, 59-67.

Liu, W., Wei, J., Cai, M., Qian, Z., Long, Z., Wang, L., Vaughn, M.G., Aaron, H.E., Tong, X., Li, Y., Yin, P., Lin, H., Zhou, M., 2022c. Particulate matter pollution and asthma mortality in China: A nationwide time-stratified case-crossover study from 2015 to 2020. Chemosphere 308, 136316.

Liu, X.-B., Wen, X.-M., Sun, X.-H., Hong, Q.-Q., Wang, Q., Kang, Z., Xia, S.-J., Yang, C., Zhu, S., 2021a. The short-term effects of ambient air pollutants are associated with daily mortality in northeast China from 2014 to 2018: a time series analysis. Journal of Occupational and Environmental Medicine 63, 173-180.

Liu, Y.-C., Gong, J., Yang, N.-N., 2012. Association between air pollution and mortality of respiratory diseases in Wuhan: a case-crossover study. Journal of Environment and Health 29, 241-244.

Liu, Y., Chen, X., Huang, S., Tian, L., Lu, Y.a., Mei, Y., Ren, M., Li, N., Liu, L., Xiang, H., 2015. Association between air pollutants and cardiovascular disease mortality in Wuhan, China. International Journal of Environmental Research and Public Health 12, 3506-3516.

Liu, Y., Pan, J., Fan, C., Xu, R., Wang, Y., Xu, C., Xie, S., Zhang, H., Cui, X., Peng, Z., 2021b. Short-term exposure to ambient air pollution and mortality from myocardial infarction. Journal of the American College of Cardiology 77, 271-281.

Liu, Y., Pan, J., Zhang, H., Shi, C., Li, G., Peng, Z., Ma, J., Zhou, Y., Zhang, L., 2019d. Short-term exposure to ambient air pollution and asthma mortality. American Journal of Respiratory and Critical Care Medicine 200, 24-32.

Lu, F., Zhou, L., Xu, Y., Zheng, T., Guo, Y., Wellenius, G.A., Bassig, B.A., Chen, X., Wang, H., Zheng, X., 2015. Short-term effects of air pollution on daily mortality and years of life lost in Nanjing, China. Science of The Total Environment 536, 123-129.

Lu, Y.-K., Liu, X.-L., Liu, Y.-H., Chen, N., Gao, H.-Y., Jin, Y.-H., Yan, Y.-X., 2023. The effects of short-term exposure to air pollution on mortality in Baotou, China, during 2015–2019. Environmental Geochemistry and Health 45, 3387-3404.

Luo, K., Li, R., Li, W., Wang, Z., Ma, X., Zhang, R., Fang, X., Wu, Z., Cao, Y., Xu, Q., 2016a. Acute effects of nitrogen dioxide on cardiovascular mortality in Beijing: an exploration of spatial heterogeneity and the district-specific predictors. Scientific Reports 6, 38328.

Luo, K., Li, W., Zhang, R., Li, R., Xu, Q., Cao, Y., 2016b. Ambient fine particulate matter exposure and risk of cardiovascular mortality: adjustment of the meteorological factors. International Journal of Environmental Research and Public Health 13, 1082.

Luo, L., Dai, Y., Zhang, F., Chen, M., Chen, F., Qing, F., 2020. Time series analysis of ambient air pollution effects on dynamic stroke mortality. The International Journal of Health Planning and Management 35, 79-103.

Ma, W.L., Li, W.L., Zhang, Z.F., Liu, L.Y., Song, W.W., Huo, C.Y., Yuan, Y.X., Li, Y.F., 2017. Occurrence and source apportionment of atmospheric halogenated flame retardants in Lhasa City in the Tibetan Plateau, China. Sci Total Environ 607-608, 1109-1116.

Ma, X., Duan, H., Zhang, H., Liu, X., Sun, X., Wei, J., Zhao, M., Xi, B., 2023. Short-term effects of PM1, PM2.5, and PM2.5 constituents on myocardial infarction mortality in qingdao, China: A time-stratified case-crossover analysis. Atmospheric Environment 294, 119478.

Ma, Y., Chen, R., Pan, G., Xu, X., Song, W., Chen, B., Kan, H., 2011. Fine particulate air pollution and daily mortality in Shenyang, China. Science of The Total Environment 409, 2473-2477.

Meng, X., Wang, C., Cao, D., Wong, C.-M., Kan, H., 2013. Short-term effect of ambient air pollution on COPD mortality in four Chinese cities. Atmospheric Environment 77, 149-154.

Mo, S., Hu, J., Yu, C., Bao, J., Shi, Z., Zhou, P., Yang, Z., Luo, S., Yin, Z., Zhang, Y., 2023. Short-term effects of fine particulate matter constituents on myocardial infarction death. Journal of Environmental Sciences 133, 60-69.

Mokoena, K.K., Ethan, C.J., Yu, Y., Shale, K., Liu, F., 2019. Ambient air pollution and respiratory mortality in Xi’an, China: a time-series analysis. Respiratory research 20, 1-9.

Niu, Y., Zhou, Y., Chen, R., Yin, P., Meng, X., Wang, W., Liu, C., Ji, J.S., Qiu, Y., Kan, H., Zhou, M., 2022. Long-term exposure to ozone and cardiovascular mortality in China: a nationwide cohort study. The Lancet Planetary Health 6, e496-e503.

Orellano, P., Reynoso, J., Quaranta, N., Bardach, A., Ciapponi, A., 2020. Short-term exposure to particulate matter (PM10 and PM2.5), nitrogen dioxide (NO2), and ozone (O3) and all-cause and cause-specific mortality: Systematic review and meta-analysis. Environment International 142, 105876.

Pan, Y., Qu, Y., Liu, J., He, Y., 2019. Time Series Analysis on PM2.5 Pollution Effect on Mortality of Cardiovascular and Cerebrovascular Diseases in Elderly People in Changchun. Journal of Environmental Hygiene 9, 8-13.

Peng, X.W., Yanding; Zhang, Xinmin; Tan, Haiping; Guo, Shu, 2023. Associations of ambient PM2.5 and O3 with human mortality: A time-series study in a city of central China. Journal of Environmental and Occupational Medicine 40, 331-341.

Peng, Z., Liu, C., Xu, B., Kan, H., Wang, W., 2017. Long-term exposure to ambient air pollution and mortality in a Chinese tuberculosis cohort. Science of The Total Environment 580, 1483-1488.

Pu, A., Guo, Y., Wu, C., Ma, R., Li, R., Li, Y., Xiang, H., Yan, Y., 2023. Short-term association between air pollution and hypertension mortality in Wuhan residents. Air Quality, Atmosphere & Health, 1-12.

Qi, F., Sha, S., Jin-fu, Z., Cai-ming, Z., 2018. Association between PM 2.5 concentration and daily resident mortality in urban area of Changsha. Journal of Environmental and Occupational Medicine 35, 131-136.

Qian, C., Yun-jie, Y., Qing-qing, W., Bin, X., Xiao-ying, M., Zhen, D., Lian, Z., Xiao-dong, C., 2017. Correlation between air pollutants and cardio-cerebrovascular mortality in Nanjing. Journal of Environmental and Occupational Medicine 34, 1041-1045.

Qian, Y., Han, M., Jin, W., Cai, R., Fang, B., Yu, H., Qian, N., Wang, C., 2015. Association between ambient air pollution and COPD mortality in Shanghai. Journal of Environmental and Occupational Medicine 32, 1093-1097.

Qian, Y., Yu, H., Cai, B., Fang, B., Wang, C., 2019. Association between incidence of fatal intracerebral hemorrhagic stroke and fine particulate air pollution. Environmental health and preventive medicine 24, 1-6.

Qian, Y., Zhu, M., Cai, B., Yang, Q., Kan, H., Song, G., Jin, W., Han, M., Wang, C., 2013. Epidemiological evidence on association between ambient air pollution and stroke mortality. J Epidemiol Community Health.

Qian, Z., He, Q., Lin, H.-M., Kong, L., Liao, D., Yang, N., Bentley, C.M., Xu, S., 2007. Short-term effects of gaseous pollutants on cause-specific mortality in Wuhan, China. Journal of the Air & Waste Management Association 57, 785-793.

Qian, Z., He, Q., Lin, H.-M., Kong, L., Zhou, D., Liang, S., Zhu, Z., Liao, D., Liu, W., Bentley, C.M., 2010. Part 2. Association of daily mortality with ambient air pollution, and effect modification by extremely high temperature in Wuhan, China. Research report (Health Effects Institute), 91-217.

Qiu, H., Pun, V.C., Tian, L., 2018. Short-term effects of fine and coarse particles on deaths in Hong Kong elderly population: An analysis of mortality displacement. Environmental Pollution 241, 148-154.

Qu, Y., Pan, Y., Niu, H., He, Y., Li, M., Li, L., Liu, J., Li, B., 2018. Short-term effects of fine particulate matter on non-accidental and circulatory diseases mortality: a time series study among the elder in Changchun. PLOS ONE 13, e0209793.

Ren, Y., 2007. The case-crossover studies of air particulate matter pollution and cardiovascular disease death. CHINA ENVIRONMENTAL SCIENCE-CHINESE EDITION- 27, 657.

Shao, M., Yu, L., Xiao, C., Deng, J., Yang, H., Xu, W., Chen, Y., Liu, X., Ni, J., Pan, F., 2021. Short-term effects of ambient temperature and pollutants on the mortality of respiratory diseases: A time-series analysis in Hefei, China. Ecotoxicology and Environmental Safety 215, 112160.

Shi, W., Sun, Q., Du, P., Tang, S., Chen, C., Sun, Z., Wang, J., Li, T., Shi, X., 2020. Modification effects of temperature on the ozone–mortality relationship: a nationwide multicounty study in China. Environmental Science & Technology 54, 2859-2868.

Shuang, W.Z., Jie, C.R., Rao, W.J., Dong, K.H., Gang, W., Fang, W.X., 2013. Short-term effects of air pollution on mortality in a heavily polluted Chinese city.

Song, J., Ding, Z., Zheng, H., Xu, Z., Cheng, J., Pan, R., Yi, W., Wei, J., Su, H., 2022. Short-term PM1 and PM2.5 exposure and asthma mortality in Jiangsu Province, China: What’s the role of neighborhood characteristics? Ecotoxicology and Environmental Safety 241, 113765.

Song, W.-M., Liu, Y., Liu, J.-Y., Tao, N.-N., Li, Y.-F., Liu, Y., Wang, L.-X., Li, H.-C., 2019. The burden of air pollution and weather condition on daily respiratory deaths among older adults in China, Jinan from 2011 to 2017. Medicine 98.

Su, C., Hampel, R., Franck, U., Wiedensohler, A., Cyrys, J., Pan, X., Wichmann, H.-E., Peters, A., Schneider, A., Breitner, S., 2015. Assessing responses of cardiovascular mortality to particulate matter air pollution for pre-, during-and post-2008 Olympics periods. ENVIRONMENTAL RESEARCH 142, 112-122.

Sui, X., Zhang, J., Zhang, Q., Sun, S., Lei, R., Zhang, C., Cheng, H., Ding, L., Ding, R., Xiao, C., 2021. The short-term effect of PM2.5/O3 on daily mortality from 2013 to 2018 in Hefei, China. Environmental Geochemistry and Health 43, 153-169.

Sun, S., Sarkar, C., Kumari, S., James, P., Cao, W., Lee, R.S.-y., Tian, L., Webster, C., 2020. Air pollution associated respiratory mortality risk alleviated by residential greenness in the Chinese Elderly Health Service Cohort. ENVIRONMENTAL RESEARCH 183, 109139.

Sun, S., Tian, L., Cao, W., Lai, P.-C., Wong, P.P.Y., Lee, R.S.-y., Mason, T.G., Krämer, A., Wong, C.-M., 2019. Urban climate modified short-term association of air pollution with pneumonia mortality in Hong Kong. Science of The Total Environment 646, 618-624.

Sun, Y., Zhang, Y., Chen, C., Sun, Q., Wang, Y., Du, H., Wang, J., Zhong, Y., Shi, W., Li, T., 2022. Impact of Heavy PM2.5 Pollution Events on Mortality in 250 Chinese Counties. Environmental Science & Technology 56, 8299-8307.

Tang, J., Chen, K., Dong, Y., Zhao, N., Li, H., 2006. A time-series study on the association of air pollution and mortality in Minhang District, Shanghai. Journal of Environmental and Occupational Medicine 23, 485-487.

Tao, Y., Huang, W., Huang, X., Zhong, L., Lu, S.-E., Li, Y., Dai, L., Zhang, Y., Zhu, T., 2012. Estimated acute effects of ambient ozone and nitrogen dioxide on mortality in the Pearl River Delta of southern China. Environmental Health Perspectives 120, 393-398.

Tian, F., Qi, J., Qian, Z., Li, H., Wang, L., Wang, C., Geiger, S.D., McMillin, S.E., Yin, P., Lin, H., Zhou, M., 2022. Differentiating the effects of air pollution on daily mortality counts and years of life lost in six Chinese megacities. Science of The Total Environment 827, 154037.

Tian, F., Qi, J., Wang, L., Yin, P., Qian, Z., Ruan, Z., Liu, J., Liu, Y., McMillin, S.E., Wang, C., Lin, H., Zhou, M., 2020a. Differentiating the effects of ambient fine and coarse particles on mortality from cardiopulmonary diseases: A nationwide multicity study. Environment International 145, 106096.

Tian, Q., Li, M., Montgomery, S., Fang, B., Wang, C., Xia, T., Cao, Y., 2020b. Short-term associations of fine particulate matter and synoptic weather types with cardiovascular mortality: an ecological time-series study in Shanghai, China. International Journal of Environmental Research and Public Health 17, 1111.

Tong, Y., Luo, K., Li, R., Pei, L., Li, A., Yang, M., Xu, Q., 2018. Association between multi-pollutant mixtures pollution and daily cardiovascular mortality: An exploration of exposure-response relationship. Atmospheric Environment 186, 136-143.

VanderWeele, T.J., Ding, P., 2017. Sensitivity analysis in observational research: introducing the E-value. Annals of internal medicine 167, 268-274.

Wang, C., Hao, L., Liu, C., Chen, R., Wang, W., Chen, Y., Yang, Y., Meng, X., Fu, Q., Ying, Z., Kan, H., 2020a. Associations between fine particulate matter constituents and daily cardiovascular mortality in Shanghai, China. Ecotoxicology and Environmental Safety 191, 110154.

Wang, J., Cao, H., Sun, D., Qi, Z., Guo, C., Peng, W., Sun, Y., Xie, Y., Liu, X., Li, B., Luo, Y., Pan, Y., Li, Y., Zhang, L., 2019a. Associations between ambient air pollution and mortality from all causes, pneumonia, and congenital heart diseases among children aged under 5 years in Beijing, China: A population-based time series study. ENVIRONMENTAL RESEARCH 176, 108531.

Wang, J., Yin, Q., Tong, S., Ren, Z., Hu, M., Zhang, H., 2017. Prolonged continuous exposure to high fine particulate matter associated with cardiovascular and respiratory disease mortality in Beijing, China. Atmospheric Environment 168, 1-7.

Wang, N., Mengersen, K., Tong, S., Kimlin, M., Zhou, M., Wang, L., Yin, P., Xu, Z., Cheng, J., Zhang, Y., Hu, W., 2019b. Short-term association between ambient air pollution and lung cancer mortality. ENVIRONMENTAL RESEARCH 179.

Wang, S., Wu, G., Du, Z., Wu, W., Ju, X., Yimaer, W., Chen, S., Zhang, Y., Li, J., Zhang, W., 2023a. The causal links between long-term exposure to major PM2. 5 components and the burden of tuberculosis in China. Science of The Total Environment 870, 161745.

Wang, X.-Q., Zhang, K.-D., Yu, W.-J., Zhao, J.-W., Huang, K., Hu, C.-Y., Zhang, X.-J., Kan, X.-H., 2023b. Associations of exposures to air pollution and greenness with mortality in a newly treated tuberculosis cohort. Environmental Science and Pollution Research 30, 34229-34242.

Wang, Y., Du, Z., Zhang, Y., Chen, S., Lin, S., Hopke, P.K., Rich, D.Q., Zhang, K., Romeiko, X.X., Deng, X., Qu, Y., Liu, Y., Lin, Z., Zhu, S., Zhang, W., Hao, Y., 2023c. Long-term exposure to particulate matter and COPD mortality: Insights from causal inference methods based on a large population cohort in southern China. Science of The Total Environment 863, 160808.

Wang, Y., Luo, S., Wei, J., Yang, Z., Hu, K., Yao, Y., Zhang, Y., 2023d. Ambient NO2 exposure hinders long-term survival of Chinese middle-aged and older adults. Science of The Total Environment 855, 158784.

Wang, Y., Shi, Z., Shen, F., Sun, J., Huang, L., Zhang, H., Chen, C., Li, T., Hu, J., 2019c. Associations of daily mortality with short-term exposure to PM2. 5 and its constituents in Shanghai, China. Chemosphere 233, 879-887.

Wang, Y., Wei, J., Zhang, Y., Guo, T., Chen, S., Wu, W., Chen, S., Li, Z., Qu, Y., Xiao, J., Deng, X., Liu, Y., Du, Z., Zhang, W., Hao, Y., 2023e. Estimating causal links of long-term exposure to particulate matters with all-cause mortality in South China. Environment International 171, 107726.

Wang, Z., Shen, Z., Wu, Y., Xiang, X., Ding, X., 2020b. Impact of atmospheric pollutant PM2.5 on deaths from respiratory diseases in the main urban areas of Chongqing from 2014 to 2018: a time-series study. Chongqing Medicine 49, 3688-3692.

Wenghaerbai, A., Sun, G., Yibulayin, X., 2018. Case-crossover study of the effect of atmospheric pollutants on respiratory disease mortality in Urumqi. Occupation and Health 34, 1243-1246+1250.

Wong, C.-M., Ma, S., Hedley, A.J., Lam, T.-H., 2001. Effect of air pollution on daily mortality in Hong Kong. Environmental Health Perspectives 109, 335-340.

Wong, C.M., Lai, H.K., Tsang, H., Thach, T.Q., Thomas, G.N., Lam, K.B.H., Chan, K.P., Yang, L., Lau, A.K., Ayres, J.G., 2015. Satellite-based estimates of long-term exposure to fine particles and association with mortality in elderly Hong Kong residents. Environmental Health Perspectives 123, 1167-1172.

Wong, T.W., Tam, W., Yu, T., Wong, A., 2002. Associations between daily mortalities from respiratory and cardiovascular diseases and air pollution in Hong Kong, China. Occupational and environmental medicine 59, 30-35.

Wu, H., Lu, K., Fu, J., 2022. A time-series study for effects of ozone on respiratory mortality and cardiovascular mortality in Nanchang, Jiangxi Province, China. FRONTIERS IN PUBLIC HEALTH 10, 864537.

Wu, R., Song, X., Chen, D., Zhong, L., Huang, X., Bai, Y., Hu, W., Ye, S., Xu, H., Feng, B., Wang, T., Zhu, Y., Fang, J., Liu, S., Chen, J., Wang, X., Zhang, Y., Huang, W., 2019a. Health benefit of air quality improvement in Guangzhou, China: Results from a long time-series analysis (2006–2016). Environment International 126, 552-559.

Wu, R., Zhong, L., Huang, X., Xu, H., Liu, S., Feng, B., Wang, T., Song, X., Bai, Y., Wu, F., Wang, X., Huang, W., 2018. Temporal variations in ambient particulate matter reduction associated short-term mortality risks in Guangzhou, China: A time-series analysis (2006–2016). Science of The Total Environment 645, 491-498.

Wu, T., Ma, Y., Wu, X., Bai, M., Peng, Y., Cai, W., Wang, Y., Zhao, J., Zhang, Z., 2019b. Association between particulate matter air pollution and cardiovascular disease mortality in Lanzhou, China. Environmental Science and Pollution Research 26, 15262-15272.

Wu, Y., Wang, Z., Li, Q., He, J., Zhang, C., Shen, Z., 2020. Relationship between PM2.5 and non-accidental deaths in main urban districts of Chongqing from 2014 to 2018. Journal of Environmental and Occupational Medicine 37, 735-740.

Xia, Y., Liu, Z., Hu, B., Rangarajan, S., Ah Tse, L., Li, Y., Wang, J., Hu, L., Wang, Y., Xiang, Q., Lin, Y., Han, G., Yusuf, S., Li, W., 2023. Associations of outdoor fine particulate air pollution and cardiovascular disease: Results from the Prospective Urban and Rural Epidemiology Study in China (PURE-China). Environment International 174, 107829.

Xie, W., Li, G., Zhao, D., Xie, X., Wei, Z., Wang, W., Wang, M., Li, G., Liu, W., Sun, J., Jia, Z., Zhang, Q., Liu, J., 2015. Relationship between fine particulate air pollution and ischaemic heart disease morbidity and mortality. Heart 101, 257-263.

Xu, D., Zhang, Y., Sun, Q., Wang, X., Li, T., 2021. Long-term PM2.5 exposure and survival among cardiovascular disease patients in Beijing, China. Environmental Science and Pollution Research 28, 47367-47374.

Xu, J., Geng, W., Geng, X., Cui, L., Ding, T., Xiao, C., Zhang, J., Tang, J., Zhai, J., 2020a. Study on the association between ambient air pollution and daily cardiovascular death in Hefei, China. Environmental Science and Pollution Research 27, 547-561.

Xu, L.-J., Shen, S.-Q., Li, L., Chen, T.-T., Zhan, Z.-Y., Ou, C.-Q., 2019. A tensor product quasi-Poisson model for estimating health effects of multiple ambient pollutants on mortality. ENVIRONMENTAL HEALTH 18, 1-10.

Xu, M., Sbihi, H., Pan, X., Brauer, M., 2020b. Modifiers of the effect of short-term variation in PM2.5 on mortality in Beijing, China. ENVIRONMENTAL RESEARCH 183, 109066.

Xu, R., Wang, Q., Wei, J., Lu, W., Wang, R., Liu, T., Wang, Y., Fan, Z., Li, Y., Xu, L., 2022. Association of short‐term exposure to ambient air pollution with mortality from ischemic and hemorrhagic stroke. European Journal of Neurology 29, 1994-2005.

Xue, X., Chen, J., Sun, B., Zhou, B., Li, X., 2018. Temporal trends in respiratory mortality and short-term effects of air pollutants in Shenyang, China. Environmental Science and Pollution Research 25, 11468-11479.

Yan, Y., She, L., Guo, Y., Zhao, Y., Zhang, P., Xiang, B., Zeng, J., Yang, M., Wang, L., 2021. Association between ambient air pollution and mortality from chronic obstructive pulmonary disease in Wuhan, China: a population-based time-series study. Environmental Science and Pollution Research 28, 33698-33706.

Yang, C., Peng, X., Huang, W., Chen, R., Xu, Z., Chen, B., Kan, H., 2012a. A time-stratified case-crossover study of fine particulate matter air pollution and mortality in Guangzhou, China. International archives of occupational and environmental health 85, 579-585.

Yang, C., Yang, H., Guo, S., Wang, Z., Xu, X., Duan, X., Kan, H., 2012b. Alternative ozone metrics and daily mortality in Suzhou: The China Air Pollution and Health Effects Study (CAPES). Science of The Total Environment 426, 83-89.

Yang, M., Pan, X., Fan, T., 2008. Time-series analysis of air pollution and cardiovascular mortality in Beijing, China. Epidemiology 19, S170-S171.

Yang, X., Liang, F., Li, J., Chen, J., Liu, F., Huang, K., Cao, J., Chen, S., Xiao, Q., Liu, X., Shen, C., Yu, L., Lu, F., Wu, X., Wu, X., Li, Y., Zhao, L., Hu, D., Huang, J., Lu, X., Liu, Y., Gu, D., 2020. Associations of long-term exposure to ambient PM2.5 with mortality in Chinese adults: A pooled analysis of cohorts in the China-PAR project. Environment International 138, 105589.

Yang, X., Zhang, L., Chen, X., Liu, F., Shan, A., Liang, F., Li, X., Wu, H., Yan, M., Ma, Z., Dong, G., Liu, Y., Chen, J., Wang, T., Zhao, B., Liu, Y., Gu, D., Tang, N., 2021. Long-term exposure to ambient PM2.5 and stroke mortality among urban residents in northern China. Ecotoxicology and Environmental Safety 213, 112063.

Yang, Y., Cao, Y., Li, W., Li, R., Wang, M., Wu, Z., Xu, Q., 2015. Multi-site time series analysis of acute effects of multiple air pollutants on respiratory mortality: A population-based study in Beijing, China. Science of The Total Environment 508, 178-187.

Yang, Y., Li, R., Li, W., Wang, M., Cao, Y., Wu, Z., Xu, Q., 2013. The association between ambient air pollution and daily mortality in Beijing after the 2008 olympics: a time series study. PLOS ONE 8, e76759.

Yang, Y., Tang, R., Qiu, H., Lai, P.-C., Wong, P., Thach, T.-Q., Allen, R., Brauer, M., Tian, L., Barratt, B., 2018. Long term exposure to air pollution and mortality in an elderly cohort in Hong Kong. Environment International 117, 99-106.

Yao, Y., Liu, L., Guo, G., Zeng, Y., Ji, J.S., 2021. Interaction of Sirtuin 1 (SIRT1) candidate longevity gene and particulate matter (PM2. 5) on all-cause mortality: a longitudinal cohort study in China. ENVIRONMENTAL HEALTH 20, 1-12.

Yin, P., Brauer, M., Cohen, A., Burnett, R.T., Liu, J., Liu, Y., Liang, R., Wang, W., Qi, J., Wang, L., 2017a. Long-term fine particulate matter exposure and nonaccidental and cause-specific mortality in a large national cohort of Chinese men. Environmental Health Perspectives 125, 117002.

Yin, P., Brauer, M., Cohen, A., Burnett, R.T., Liu, J., Liu, Y., Zhou, M., 2015. Ambient fine particulate matter exposure and cardiovascular mortality in China: a prospective cohort study. The Lancet 386, S6.

Yin, P., Chen, R., Wang, L., Meng, X., Liu, C., Niu, Y., Lin, Z., Liu, Y., Liu, J., Qi, J., 2017b. Ambient ozone pollution and daily mortality: a nationwide study in 272 Chinese cities. Environmental Health Perspectives 125, 117006.

Yin, W.-J., Peng, X.-W., Song, S.-Z., 2012. Air pollution and the cerebro cardio-vascular diseases mortality of population in Guangzhou: a time-series analysis. Journal of Environment and Health 29, 521-525.

Yin, Y., Zhong, W., Lin, L., Lin, X., Jiang, D., Huang, S., Huang, Y., Zhu, Y., Chen, J., Ye, Y., 2019. Association between atmospheric particulates and acute cardiovascular and cerebrovascular events mortality in Sanming: a time-series analysis. Chronic Pathematol J 20, 1606-1610.

Yu, B., Lu, H., Wang, Y., 2006. A time-series study on the association of daily mortality and air pollution in Luwan district, Shanghai. Shanghai Journal of Preventive Medicine, 264-266.

Yu, I.T.S., hui Zhang, Y., San Tam, W.W., Yan, Q.H., jun Xu, Y., jun Xun, X., Wu, W., Ma, W.J., Tian, L.W., Tse, L.A., 2012. Effect of ambient air pollution on daily mortality rates in Guangzhou, China. Atmospheric Environment 46, 528-535.

Yu, Y., Dong, H., Yao, S., Ji, M., Yao, X., Zhang, Z., 2017. Protective effects of ambient ozone on incidence and outcomes of ischemic stroke in Changzhou, China: a time-series study. International Journal of Environmental Research and Public Health 14, 1610.

Yu, Y., Yao, S., Dong, H., Wang, L., Wang, C., Ji, X., Ji, M., Yao, X., Zhang, Z., 2019. Association between short-term exposure to particulate matter air pollution and cause-specific mortality in Changzhou, China. ENVIRONMENTAL RESEARCH 170, 7-15.

Yuan, J., Li, M., Fu, J., Chen, J., Ren, Q., Xiao, C., 2022. Time Series of PM2.5 and Death of Residents from Respiratory Disease and Malignant Tumors in a Certain Area. Journal of Jinggangshan University(Natural Science) 43, 86-92.

Yuan, Y., Wang, K., Sun, H.Z., Zhan, Y., Yang, Z., Hu, K., Zhang, Y., 2023. Excess mortality associated with high ozone exposure: A national cohort study in China. Environmental Science and Ecotechnology 15, 100241.

Zai-sheng, L., Shao-kai, L., Kai, W., Cui-ying, L., Xiao-hai, Z., 2020. Time-series study on effects of low-concentration PM 2.5 on mortality in elderly people in Fuzhou from 2015 to 2018. Journal of Environmental and Occupational Medicine 37, 157-161.

Zeng, J., Liu, R., Chen, J., Jin, L., Jiang, X., Zhou, L., Zhang, L., 2017. Correlation between PM2. 5 concentration and mortality of respiratory diseases in Chengdu: A time series analysis. Jiangsu J. Prev. Med 28, 144-147.

Zhai, S., Zeng, J., Zhang, Y., Huang, J., Li, X., Wang, W., Zhang, T., Deng, Y., Yin, F., Ma, Y., 2023. Combined health effects of PM2.5 components on respiratory mortality in short-term exposure using BKMR: A case study in Sichuan, China. Science of The Total Environment 897, 165365.

Zhang, C., Ding, R., Xiao, C., Xu, Y., Cheng, H., Zhu, F., Lei, R., Di, D., Zhao, Q., Cao, J., 2017a. Association between air pollution and cardiovascular mortality in Hefei, China: A time-series analysis. Environmental Pollution 229, 790-797.

Zhang, F., Li, L., Krafft, T., Lv, J., Wang, W., Pei, D., 2011a. Study on the association between ambient air pollution and daily cardiovascular and respiratory mortality in an urban district of Beijing. Int J Environ Res Public Health 8, 2109-2123.

Zhang, F., Li, L., Krafft, T., Wang, L., Zhang, J., Lin, L., Li, Q., Shi, Y., Wang, W., 2014. Time-Series Study on Air Pollution and Mortality. POLISH JOURNAL OF ENVIRONMENTAL STUDIES 23.

Zhang, F., Liu, X., Zhou, L., Yu, Y., Wang, L., Lu, J., Wang, W., Krafft, T., 2016. Spatiotemporal patterns of particulate matter (PM) and associations between PM and mortality in Shenzhen, China. BMC PUBLIC HEALTH 16, 1-11.

Zhang, J., Chen, Q., Wang, Q., Ding, Z., Sun, H., Xu, Y., 2019. The acute health effects of ozone and PM2.5 on daily cardiovascular disease mortality: A multi-center time series study in China. Ecotoxicology and Environmental Safety 174, 218-223.

Zhang, J., Guo, Y., 2010. Time-series analysis on relationship between air pollution and daily mortality in Chaoyang district, Beijing. Journal of Environment and Health 27, 797-799.

Zhang, J., Liu, Y., Cui, L.-l., Liu, S.-q., Yin, X.-x., Li, H.-c., 2017b. Ambient air pollution, smog episodes and mortality in Jinan, China. Scientific Reports 7, 11209.

Zhang, J., Meng, H., 2011. Relationship between air pollution and daily respiratory system disease mortality in Chaoyang district, Beijing: a time-series analysis. Journal of Environment and Health 28, 788-791.

Zhang, K.J., Wu; Yao, Qingbin; Han, Xiaoliang; Li, Xiaoqin; E, Meng, 2019a. Time-series analysis of association between ozone and daily mortality in Yangzhou. Journal of Modern Medicine & Health 35, 3265-3266+3271.

Zhang, K.J., Wu; Yao, Qingbin; Han, Xiaoliang; Li, Xiaoqin; E, Meng, 2019b. Time series analysis of the relationship between air fine particulate matter and resident deaths in Yangzhou City. Journal of Nantong University (Medical Edition) 39, 299-301.

Zhang, P., Dong, G., Sun, B., Zhang, L., Chen, X., Ma, N., Yu, F., Guo, H., Huang, H., Lee, Y.L., 2011b. Long-term exposure to ambient air pollution and mortality due to cardiovascular disease and cerebrovascular disease in Shenyang, China. PLOS ONE 6, e20827.

Zhang, R., Liu, G., Jiang, Y., Li, G., Pan, Y., Wang, Y., Wei, Z., Wang, J., Wang, Y., 2018. Acute effects of particulate air pollution on ischemic stroke and hemorrhagic stroke mortality. Frontiers in neurology 9, 827.

Zhang, W.L., Lijian, 2023. Time series analysis on the effect between atmospheric PM2.5 pollution and deaths from cardiovascular diseases among residents in Yangquan City, 2019-2020. Practical Preventive Medicine 30, 908-912.

Zhang, Y., 2021. All-cause mortality risk and attributable deaths associated with long-term exposure to ambient PM2. 5 in Chinese adults. Environmental Science & Technology 55, 6116-6127.

Zhang, Y., Huang, W., London, S.J., Song, G., Chen, G., Jiang, L., Zhao, N., Chen, B., Kan, H., 2006. Ozone and daily mortality in Shanghai, China. Environmental Health Perspectives 114, 1227-1232.

Zhang, Y., Li, Z., Wei, J., Zhan, Y., Liu, L., Yang, Z., Zhang, Y., Liu, R., Ma, Z., 2022a. Long-term exposure to ambient NO_2_ and adult mortality: A nationwide cohort study in China. Journal of Advanced Research 41, 13-22.

Zhang, Y., Ma, Y., Shen, J., Li, H., Wang, H., Cheng, B., Ma, L., 2022b. Effect of ambient O3 on mortality due to circulatory and respiratory diseases in a high latitude city of northeast China. Environmental Science and Pollution Research 29, 67776-67786.

Zhang, Y., Tian, Q., Feng, X., Hu, W., Ma, P., Xin, J., Wang, S., Zheng, C., 2022c. Modification effects of ambient temperature on ozone-mortality relationships in Chengdu, China. Environmental Science and Pollution Research 29, 73011-73019.

Zhang, Y., Wang, S., Zhang, X., Ni, C., Zhang, J., Zheng, C., 2020a. Temperature modulation of the adverse consequences on human mortality due to exposure to fine particulates: A study of multiple cities in China. ENVIRONMENTAL RESEARCH 185, 109353.

Zhang, Y., Wang, Y., Du, Z., Chen, S., Qu, Y., Hao, C., Ju, X., Lin, Z., Wu, W., Xiao, J., 2023a. Potential causal links between long-term ambient particulate matter exposure and cardiovascular mortality: New evidence from a large community-based cohort in South China. Ecotoxicology and Environmental Safety 254, 114730.

Zhang, Y., Wu, K., Zhu, C., Feng, R., Li, C., Ma, L., 2015. Association between ambient air pollution and stroke mortality in Wuhan, China: a time-series analysis. Zhonghua yu Fang yi xue za zhi [Chinese Journal of Preventive Medicine] 49, 605-610.

Zhang, Y., Xiang, Q., Yu, C., Yang, Z., 2020b. Asthma mortality is triggered by short-term exposures to ambient air pollutants: Evidence from a Chinese urban population. Atmospheric Environment 223, 117271.

Zhang, Y., Yin, Z., Li, S., Zhang, J.J., Sun, H.Z., Liu, K., Shirai, K., Hu, K., Qiu, C., Liu, X., 2023b. Ambient PM2.5, ozone and mortality in Chinese older adults: A nationwide cohort analysis (2005–2018). Journal of Hazardous Materials 454, 131539.

Zhao, J.-W., Wang, X.-Q., Li, Z.-H., Mao, Y.-C., Zhang, S., Huang, K., Hu, C.-Y., Zhang, X.-J., Kan, X.-H., 2023. Effect of gaseous pollutant and greenness exposure on mortality during treatment of newly treated tuberculosis patients: a provincial population-based cohort study. Environmental Science and Pollution Research 30, 98195-98210.

Zhao, Y., An, X., Sun, Z., Li, Y., Hou, Q., 2022. Identification of health effects of complex air pollution in China. International Journal of Environmental Research and Public Health 19, 12652.

Zhong, P., Huang, S., Zhang, X., Wu, S., Zhu, Y., Li, Y., Ma, L., 2018. Individual-level modifiers of the acute effects of air pollution on mortality in Wuhan, China. Global health research and policy 3, 1-9.

Zhou, H., Geng, H., Dong, C., Bai, T., 2021. The short-term harvesting effects of ambient particulate matter on mortality in Taiyuan elderly residents: A time-series analysis with a generalized additive distributed lag model. Ecotoxicology and Environmental Safety 207, 111235.

Zhou, P., Hu, J., Yu, C., Bao, J., Luo, S., Shi, Z., Yuan, Y., Mo, S., Yin, Z., Zhang, Y., 2022. Short-term exposure to fine particulate matter constituents and mortality: case-crossover evidence from 32 counties in China. Science China Life Sciences 65, 2527-2538.

Zhou, Q., Lin, X., Lu, Y., 2018. Influence of air pollution on residents' death in Fuzhou urban area by time series analysis, 2015-2017. Strait Journal of Preventive Medicine 24, 15-17+20.

Zhu, F., Chen, L., Qian, Z., Liao, Y., Zhang, Z., McMillin, S.E., Wang, X., Lin, H., 2021. Acute effects of particulate matter with different sizes on respiratory mortality in Shenzhen, China. Environmental Science and Pollution Research 28, 37195-37203.

Zhu, Y., Zhong, W., Lin, L., Huang, S., Jiang, D., Lin, X., Hu, Q., Chen, J., Li, L., 2019. Time-series analysis on relationship between particulate matter concentration and ischemic heart disease mortality,Sanming city. Preventive Medicine Tribune 25, 727-730.
